# Examining the efficacy of a telehealth intervention targeting addictive eating in Australian adults (the TRACE Programme): a randomised controlled trial protocol

**DOI:** 10.1136/bmjopen-2022-064151

**Published:** 2023-06-06

**Authors:** Janelle A Skinner, Megan Whatnall, Mark Leary, Rebecca A Collins, Kirrilly M Pursey, Antonio Verdejo-García, Phillipa J Hay, Amanda L Baker, Leanne Hides, Susan J Paxton, Lisa G Wood, Kim Colyvas, Clare E Collins, Tracy L Burrows

**Affiliations:** 1 School of Health Sciences, The University of Newcastle, Callaghan, New South Wales, Australia; 2 Food and Nutrition Research Program, Hunter Medical Research Institute, New Lambton Heights, New South Wales, Australia; 3 School of Psychological Sciences and the Turner Institute for Brain and Mental Health, Monash University, Clayton, Victoria, Australia; 4 Translational Health Research Institute and School of Medicine, Western Sydney University, Penrith South, New South Wales, Australia; 5 School of Medicine and Public Health, The University of Newcastle, Callaghan, New South Wales, Australia; 6 School of Psychology, University of Queensland, Brisbane, Queensland, Australia; 7 School of Psychology and Public Health, La Trobe University, Melbourne, Victoria, Australia; 8 School of Biomedical Sciences and Pharmacy, The University of Newcastle, Callaghan, New South Wales, Australia; 9 Viruses, Infections / Immunity, Vaccines and Asthma Research Program, Hunter Medical Research Institute, New Lambton, New South Wales, Australia; 10 School of Mathematical and Physical Sciences, The University of Newcastle, Callaghan, New South Wales, Australia

**Keywords:** nutrition & dietetics, public health, eating disorders

## Abstract

**Introduction:**

Approximately 15%–20% of the adult population self-report symptoms of addictive eating. There are currently limited options for management. Motivational interviewing-based interventions, containing personalised coping skills training, have been found to be effective for behaviour change in addictive disorders (eg, alcohol). This project builds upon foundations of an addictive eating feasibility study previously conducted and co-design process involving consumers. The primary aim of this study is to examine the efficacy of a telehealth intervention targeting addictive eating symptoms in Australian adults compared with passive intervention and control groups.

**Methods and analysis:**

This three-arm randomised controlled trial will recruit participants 18–85 years, endorsing ≥3 symptoms on the Yale Food Addiction Scale (YFAS) 2.0, with body mass index >18.5 kg/m^2^. Addictive eating symptoms are assessed at baseline (pre-intervention), 3 months (post-intervention) and 6 months. Other outcomes include dietary intake and quality, depression, anxiety, stress, quality of life, physical activity and sleep hygiene. Using a multicomponent clinician-led approach, the active intervention consists of five telehealth sessions (15–45 min each) delivered by a dietitian over 3 months. The intervention uses personalised feedback, skill-building exercises, reflective activities and goal setting. Participants are provided with a workbook and website access. The passive intervention group receives the intervention via a self-guided approach with access to the workbook and website (no telehealth). The control group receives personalised written dietary feedback at baseline and participants advised to follow their usual dietary pattern for 6 months. The control group will be offered the passive intervention after 6 months. The primary endpoint is YFAS symptom scores at 3 months. A cost–consequence analysis will determine intervention costs alongside mean change outcomes.

**Ethics and dissemination:**

Human Research Ethics Committee of University of Newcastle, Australia provided approval (H-2021-0100). Findings will be disseminated via publication in peer-reviewed journals, conference presentations, community presentations and student theses.

**Trial registration number:**

Australia New Zealand Clinical Trials Registry (ACTRN12621001079831).

**STRENGTHS AND LIMITATIONS OF THIS STUDY**

- Targeting addictive risk factors through personalised tailoring of coping strategies and use of motivational interviewing for management of symptoms of addictive eating.
- Co-design approach taken, with both consumers and multidisciplinary health professionals, to inform programme development.
- Detailed assessment of eating behaviours, mental health and lifestyle factors with personalised feedback provided to participants during the telehealth intervention.
- Fidelity outcomes will be assessed and cost–consequence analysis conducted regarding implementation.
- Limitations include participants being excluded with severe mental illnesses or complex health conditions.

## Introduction

Research in addictive eating has increased rapidly in recent years. Addictive eating, theorised as being on the severe end of a spectrum of overeating,^1^ is a phenotype of eating behaviour marked by the chronic excessive and dysregulated consumption of food.^2 3^ Addictive eating, not categorised as a distinct disorder in the Diagnostic and Statistical Manual of Mental Disorders (DSM)^4^ or the International Classification of Disease^5^ systems, is most commonly assessed using the Yale Food Addiction Scale (YFAS).^6^ The YFAS adapts the DSM criteria for ‘substance-related and addictive disorders’ to specific foods.^4^ Developed in 2009^2^ and revised in 2016 (YFAS 2.0)^6^ according to the DSM-5 criteria, this psychometric tool assesses the presence of 11 symptoms of addictive eating. Symptoms include craving, loss of control, tolerance and withdrawal associated with eating behaviours, the repeated unsuccessful attempts to reduce the consumption of specific foods and maintenance of these behaviours despite adverse physical/emotional/social/interpersonal consequences.^6^ The YFAS 2.0 provides two scoring options: a continuous symptom score, reflecting the number of endorsed addiction-like symptoms; and a dichotomous diagnosis of ‘food addiction’.^6^ Using this self-report survey, approximately 15%–20% of the adult population endorse ≥three YFAS symptoms for addictive eating.^7–9^

Higher prevalence rates of addictive eating have been reported in individuals with higher body mass indexes (BMIs) classified as overweight or obese compared with lower BMIs,^8 10^ although addictive eating is not exclusive to those with higher weight status.^11^ It has been suggested that addictive eating in those with underweight may be related to dietary restriction practices (eg, consuming more than intended that breaches self-imposed dietary rules, intense craving resulting from extreme dieting practices).^11 12^ Irrespective of weight status, results from recent research indicate that individuals with addictive eating have significantly lower diet quality and higher intake of highly processed foods.^9 13 14^ Poor diet is a significant contributor to early death globally,^15^ and addressing addictive eating may contribute to the prevention or management of adverse health outcomes.

Addictive eating is a complex issue often overlapping with other health conditions and likely transdiagnostic.^16 17^ There is evidence that addictive eating commonly co-occurs with mental health comorbidities, particularly depression and anxiety, as well as overlapping with eating disorders, specifically binge eating disorder (BED).^7 14^ Approximately 50% of individuals with BED meet criteria for ‘food addiction’ according to the YFAS.^7^ The present state of the literature demonstrates there is considerable overlap between BED and addictive eating.^18 19^ Commonalities include the loss of control over consumption, continued overuse despite negative consequences and repeated failed attempts to reduce consumption.^19^ At this time, it is unclear if addictive eating will emerge as a severe subtype of BED or be regarded as a distinct form of an addiction disorder. This distinction will be important to allow for targeted treatment and prevention strategies in susceptible individuals.

Current treatment options for addictive eating largely stem from online self-help groups such as Food Addicts Anonymous^20^ and Overeaters Anonymous.^21^ Originating in the USA, they now have 10 000+ members worldwide and have been in existence for many years, demonstrating a need for services.^22^ A 2021 systematic review^23^ found there is limited evidence supporting implementation of feasible and effective dietary interventions run by clinicians, for the management of addictive eating.^24^ Of the nine studies reviewed, five studies that included lifestyle modification,^25^ medication^26 27^ or bariatric surgery^28 29^ were found to improve symptoms of addictive eating.^23^ Since publication of this review, a further four intervention studies (a behavioural weight loss programme,^30^ a brief telephone-based cognitive–behavioural therapy intervention,^31^ a low-carbohydrate dietary programme^32^ and a probiotic supplement placebo-controlled trial^33^) have been trialled with improvement in YFAS addictive eating symptomatology immediately following the intervention. To date, most studies have been limited in sample size and therefore have not been powered to detect a change in addictive eating symptoms.^23^ Given the limited number of treatment options for addictive eating, there is a clear need for services, and development and testing of interventions. It has been suggested that interventions based on substance use addiction models may be effective at facilitating changes in eating behaviour.^34^

Motivational interviewing (MI)-based interventions for addictive disorders, such as alcohol use, in combination with coping skills training for traits associated with risk of addictive behaviour, have found to be effective.^35 36^ The traits that have been linked to addictive eating include impulsivity, sensation seeking, and anxiety and depression proneness.^37–41^ Findings suggest that individuals with addictive eating may be highly aware of emotions, but lack the skills needed to cope with negative affect.^42^ Using personalised coping skills for traits associated with personality and the risk of addictive behaviour in combination with MI, a communication approach used to identify and resolve ambivalence between desired behaviours and actual behaviours to increase motivation,^43^ may be affective to facilitate behaviour change in individuals with addictive eating.

Telehealth has been shown to be a strategy to increase reach, with virtual sessions being comparable with face-to-face programmes, and to increase access to services without compromising effectiveness.^44^ Telehealth will allow participants from anywhere in Australia to participate from home, and will reduce the demands on time and cost of travel.^44^ Additionally, telehealth may overcome client-centred barriers by allowing a safe atmosphere for some participants to better engage and discuss more sensitive topics that they would not normally raise.^45^ Although the effectiveness of telehealth has not been explicitly explored in populations with addictive eating, recent research demonstrates that telehealth can be as effective as in-person care for the management of mental health conditions,^46^ including substance use disorders^47^ and eating disorders.^48^

This project builds on a programme of work that included an initial feasibility study for the management of addictive eating in adults (Australia New Zealand Clinical Trial Registry ACTRN12619001540101).^24^ Results from the initial study indicated that the programme was feasible in the target population. Feedback received from programme participants and facilitators identified a need for a greater number of programme sessions and improved strategies for increasing retention. As a result, the programme was further refined with consumers using an integrated knowledge translation (iKT) framework.^49^ This co-design phase included consumers with lived experience, as well as health professionals from a range of disciplines, to ensure the culmination of multidisciplinary evidence-based strategies was included. This was unique as previous reports omit this co-design step or are siloed in their approach.^49^ The co-design process used a series of interviews and workshops to gain input into the programme overview, aims, content and materials. Subsequent changes were made to the programme content and language used, and materials were created or refined to improve acceptability. The resultant behaviour change intervention, the TRACE (Targeted Research on Addictive and Compulsive Eating) Programme, is a complex intervention and previously described using the Medical Research Council TIDieR (Template for Intervention Description and Replication) checklist for complex interventions^50^) (see Leary *et al* 49 for the TIDieR checklist of the intervention).

To the authors’ knowledge, the TRACE Programme is the first MI-based telehealth intervention used in combination with personalised coping skills training for the management of addictive eating in adults. The aim of the current study is to determine the efficacy of a telehealth intervention (active intervention) to reduce symptoms of addictive eating in adults, relative to passive intervention and control (no intervention) groups. It is hypothesised that both the active and passive intervention groups will achieve a reduction in addictive eating symptoms relative to the control group. Potential moderators (eg, participant sociodemographics) and mediators (eg, physical activity, diet and sleep) of intervention efficacy will also be evaluated.

## Methods

### Study trial design

The TRACE Programme is a randomised controlled trial with three parallel arms. Assessments will be carried out at three time points: (1) baseline (pre-intervention), (2) 3-month post-baseline (primary time point) and (3) 6-month post-baseline follow-up assessment. This project was approved by the University of Newcastle Human Research Ethics Committee (H-2021-0100) and prospectively registered with the Australian New Zealand Clinical Trials Registry (ACTRN12621001079831). The study protocol was developed in accordance with the Standard Protocol Items: Recommendations for Interventional Trials (SPIRIT) guidelines.^51^ The design, conduct and reporting of the studies will adhere to the CONSORT (Consolidated Standards of Reporting Trials) guidelines.^52^ All participants will provide informed electronic consent (see online supplemental material 1 for a copy of the consent form) to participate and can withdraw at any time for any reason. The funding bodies had no role in the design, conduct or reporting of the study.

10.1136/bmjopen-2022-064151.supp1 Supplementary data

### Setting

The active intervention will be delivered via telehealth sessions, conducted in Australia, and supported by a programme workbook and website containing materials relevant to the intervention.

### Recruitment

Participants will be recruited using a range of strategies including media releases, advertising via local and national newspapers, and social media releases. Informed by our iKT process, a range of recruitment videos (tailored for gender) were also created in addition to written material, which will be released via Twitter and Facebook. A non-probability sampling technique (voluntary response sampling)^53^ will be used, and recruitment will continue until the desired number of participants is achieved. Recruitment commenced in August 2021 and was completed in April 2022. Recruitment materials will direct individuals to the study information sheet and eligibility survey. The eligibility survey takes approximately 15 min to complete (table 1). Online informed consent will be obtained prior to completing the eligibility survey.

**Table 1:** Schedule of measurements

Variable	Instrument	Enrolment	Time point post-allocation		
Primary study		Eligibility screening	t_1_ Baseline	t_2_ 3 months	t_3_ 6 months
Sample characteristics					
Demographics	Age, sex, postcode, mental health status	**✓**			
Socioeconomic factors	Education, income, marital status, employment status, occupation and living/accommodation status	**✓**			
Anthropometrics	Self-report height and weight	**✓**		**✓**	**✓**
Smoking and substance use	Alcohol, Smoking and Substance Involvement Screening Test V.3.0^59^		**✓**	**✓**	**✓**
Purging behaviours	Eating Disorder Examination Questionnaire–Short form^54^	**✓**			
Primary outcomes					
Addictive eating symptoms and severity	Yale Food Addiction Scale 2.0^6^	**✓**		**✓**	**✓**
Secondary outcomes					
Dietary intake and quality	Australian Eating Survey^69 70^		**✓**	**✓**	**✓**
Depression, anxiety and stress	Patient Health Questionnaire-8,^63^ Generalized Anxiety Disorder 7,^60^ Perceived Stress Scale^61^	**✓**		**✓**	**✓**
Mediators/moderators					
Trait/s associated with risk of addictive behaviour	Substance Use Risk Profile Scale^81^		**✓**		
Eating behaviours	Eating Disorder Examination Questionnaire 6.0,^62^ Binge Eating Scale,^82^ Short Inventory of Grazing,^83^ Reward-Based Eating Drive Scale^84^		**✓**	**✓**	**✓**
Participant activation level	Patient Activation Measure 13^64^	**✓**		**✓**	**✓**
Usage and engagement with programme website	Google Analytics (Google) to record number of site visits, visit duration, number of page views and links accessed/resources downloaded		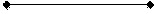		
Usage and engagement with Facebook group	Number of participants to join group; number of views, likes and comments per post manually recorded			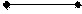	
Other outcomes					
Quality of life	EQ-5D-5L^74^		**✓**	**✓**	**✓**
Physical activity level	Active Australia Survey^75^		**✓**	**✓**	**✓**
Sleep hygiene behaviours	Pittsburgh Sleep Quality Index^76^		**✓**	**✓**	**✓**
Healthcare utilisation	Consumer Services Receipt Inventory^77^		**✓**	**✓**	**✓**
‘Control’ and ‘compulsion’ associated with addictive eating	Qualitative analysis of a segment of the first telehealth session		**✓**		

Eligibility screening=assessment of inclusion/exclusion criteria, baseline=pre-intervention, 3 months=immediate post-intervention, 6 months=3 months post-intervention.

### Eligibility

To be eligible for inclusion in the study, individuals must:

1. Be aged between 18 years and 85 years.
2. Endorse ≥3 symptoms on the YFAS 2.0 (ie, exhibiting mild to severe addictive eating).^6^
3. Have a self-reported weight and height consistent with a BMI ≥18.5 kg/m^2^.
4. Be competent in the English language.
5. Live in Australia.
6. Have access to the internet.

Individuals will be excluded from participating in the study if they:

1. Are pregnant or lactating.
2. Report having a severe mental illness (including schizophrenia or bipolar disorder) or have a health condition that necessitates taking medications, which affect dietary intake or weight status.
3. Report purging behaviours as identified by the Eating Disorder Examination Questionnaire–Short form (EDE-QS).^54^

#### Methodological considerations for eligibility criteria

The eligibility screener excludes individuals with a BMI below 18.5 kg/m^2^. This measure was put in place to reduce the likelihood of recruiting participants with at-risk restrictive eating practices that may be influencing a relatively low weight status. The value of <18.5 kg/m^2^ was chosen as this is below the current healthy weight range in national guidelines for Australians^55^ and the Centers for Disease Control and Prevention in the USA.^56^ Additionally, the eligibility screener includes the EDE-QS.^54^ This 12-item validated tool is commonly used to identify potential eating disorders. Based on the research team consensus, individuals who have compensatory behaviours such as bingeing/purging (specifically asked in question 7 on the EDE-QS), who may be at risk of an eating disorder and are medically compromised, will be deemed not eligible for the current study. Purging is related to higher levels of appearance dissatisfaction, anxiety and depressive symptoms and self-concept instability.^57 58^ As per the ethics protocol, participants endorsing any response to this question, indicating these compensatory behaviours, will be excluded from the study. The tools for eating disorders and psychological health^54 59–63^ used in the study have been widely used in research in the areas of eating disorders, dietary interventions, substance use and mental health and are considered standard tools for their specific measures. The participation information sheet and the last page of the study surveys, provide links for reputable health organistions that participants may contact to obtain further assistance with health behaviours.

### Study procedure

Prospective participants will complete the eligibility survey. This will include demographic questions (eg, sex, postcode, marital status, level of education, employment status); the YFAS 2.0^6^ to confirm endorsement of ≥3 addictive eating symptoms; and the EDE-QS^31^ to confirm the absence of purging behaviours. While not necessary to determine eligibility, the Patient Health Questionnaire-8 (PHQ-8),^63^ Generalized Anxiety Disorder-7 (GAD-7),^60^ Perceived Stress Scale-4,^61^ Patient Activation Measure 13^64^ and two questions relating to previous treatments sought for addictive eating will also be completed by potential participants. These questions have been specifically added to extend our previously reported research^65^ regarding the types of individuals recruited into interventions for addictive eating.

**Figure 1:**
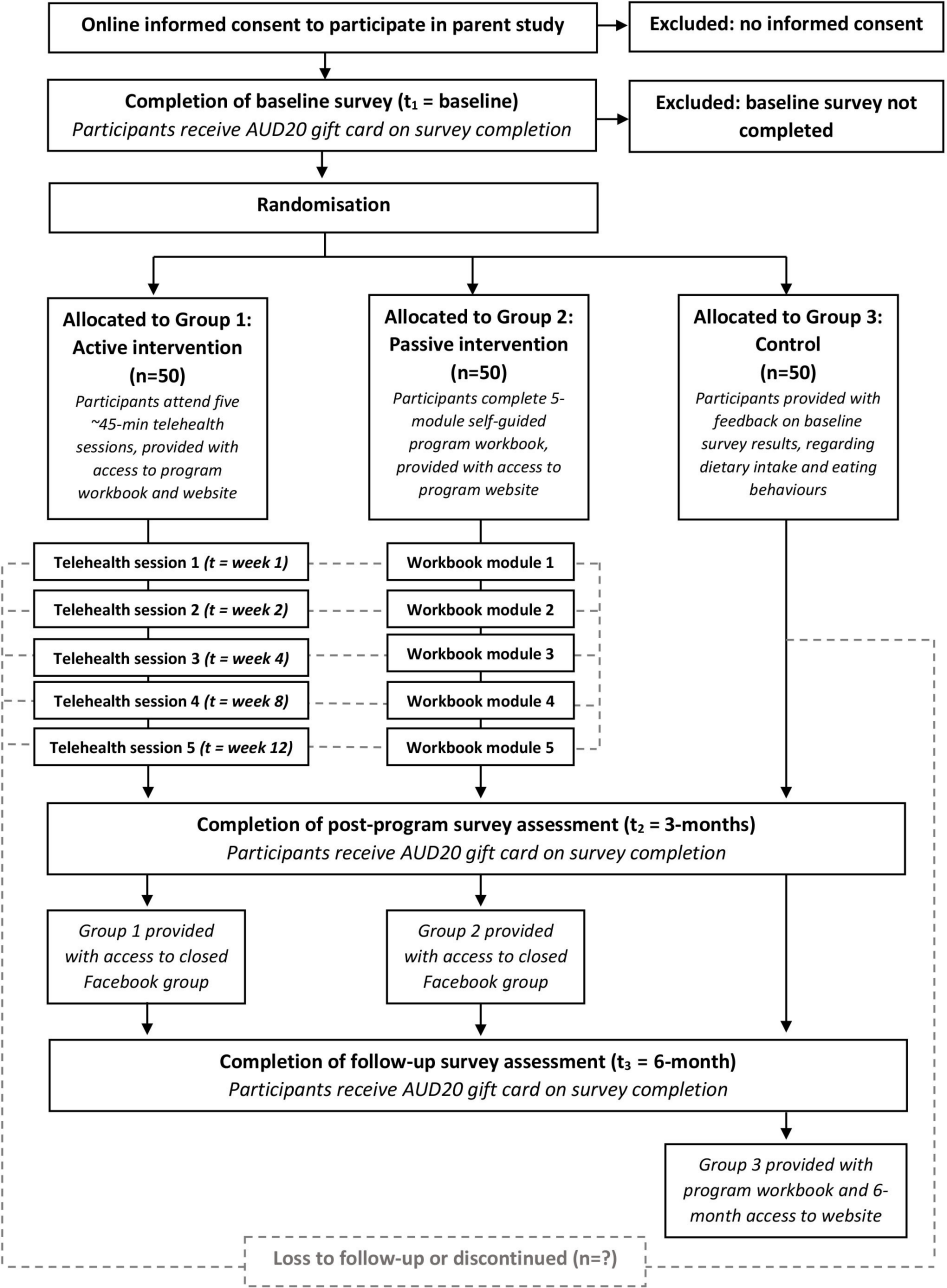
Overview of the study schedule. AUD, Australian dollar.

Participants deemed eligible will proceed to the online consent form (figure 1—overview of study schedule). Participants will be given a 2-week period to consider participation. After this time, a member of the research team will contact any individuals via email who have not completed the consent form to determine their interest in participating. Following this, no other contact will be made. Participants who provide electronic written consent will complete the baseline assessment surveys measuring dietary intake and eating habits, traits associated with personality and risk of addictive behaviour, quality of life and healthcare service utilisation (table 1—schedule of measurements). The surveys take approximately 40 min to complete. On completion of baseline surveys, participants will be randomly allocated to one of three groups (group 1: active intervention; group 2: passive intervention; or group 3: control; see the Intervention section) and informed of their group allocation via email.

Following randomisation (see the Randomisation and blinding section), a member of the research team will contact participants in group 1 via telephone or email to arrange an appointment time for their initial telehealth session. Groups 1 and 2 will be emailed a copy of the programme workbook (printable and fillable PDF versions); a hard copy is available for participants on request and be provided with password-protected access to the programme website at this time. Telehealth sessions 2–5, for participants allocated to the active intervention group (group 1), will be arranged during their first telehealth session.

Participants from all three groups will receive results from the eligibility and/or baseline surveys by the research team via email. On survey completion, groups 1 and 2 will receive feedback on dominant trait/s that may be associated with increased risk of addictive behaviours (eg, anxiety proneness, impulsivity proneness); symptoms of addictive eating; dietary, caffeine and alcohol intake; sleep hygiene and physical activity levels. At this time point, group 3 will only receive feedback on dietary intake via email. At 6 months post-study commencement, group 3 will be provided with feedback on trait/s associated with personality and risk of addictive behaviour; symptoms of addictive eating; sleep hygiene and physical activity levels, along with access to the workbook and website (the passive intervention that group 2 received at baseline). To ensure consistency across participants, email templates and standardised reports will be used by the research team. Group 2 will be guided with written instructions in their workbook on how to use their survey results to allow personalised goal setting regarding their dietary intake and eating patterns.

The primary and secondary outcomes will be assessed at 3 months (primary endpoint, immediate post-active intervention period) and 6 months (follow-up) where participants will complete post-programme surveys (table 1—schedule of measurements). Participants will be sent reminder emails to complete their surveys. They will be reminded a maximum of three times at each time point. If no contact is received after such time, no further contact will be made. Participants will be remunerated with a gift voucher to the value of $A20 at the completion of baseline, 3-month and 6-month surveys, corresponding to a maximum of $A60 per participant over the course of the study.

### Randomisation and blinding

Following completion of baseline assessments, participants will be stratified into four groups by sex and mental health status (presence or absence, based on either depression scale PHQ-8 scores ≥15 or below 15, or anxiety scale GAD-7 scores ≥11 or below 11). Participants within each of these four groups will be randomised to one of the three study groups in equal ratios using permuted block randomisation, with block sizes of six. Randomisation will promote group balance on these variables shown to be important in past cross-sectional research (for example, 7 8 10 65). The randomisation sequence will be generated by an independent statistician and implemented by a designated study coordinator. The allocation list will be stored in a password-protected computer file and accessed only by the study coordinator.

Due to the telehealth nature of the active intervention, blinding of participants and dietitians to intervention group allocation in this study will not be possible. However, several strategies will be employed to reduce the risk of bias. First, participants will only be provided with partial information on the study hypotheses. Second, all communication between participants and research staff during the period of intervention (ie, scheduling concerns, questions regarding the intervention) will be done directly between participants and the ‘study coordinator’ or their respective ‘telehealth clinician’. Lastly, statistical analyses will be conducted by researchers who are blind to group allocation prior to analysis.

### Intervention

The intervention study arms are:

Group 1—active intervention: targeting change in addictive eating behaviours using a multicomponent clinician-led approach (telehealth sessions, programme workbook and programme website).

Group 2—passive intervention: targeting change in addictive eating behaviours using a multicomponent self-guided approach (programme workbook and programme website).

Group 3—control dietary feedback, via paper-based report, provided at baseline, and participants follow their usual dietary pattern for 6 months.

The comparator groups were chosen to provide a passive delivery option of the programme, which would be consistent with a self-guided cognitive–behavioural therapy approach (group 2), and a control group consistent with a standard version of dietary feedback (group 3). The control group is not a wait list control; however, participants in this group will be offered access to the passive intervention (ie, programme workbook and programme website) after the completion of the 6-month assessment.

### TRACE active intervention (group 1)

Participants will receive five standardised one-on-one telehealth/phone sessions with an Accredited Practising Dietitian, with training in behaviour change and eating disorders, over a 3-month period (ie, weeks 1, 2, 4, 8 and 12). Additionally, dietitians leading the intervention delivery will have extensive experience in private practice work and working with clients including those with disordered eating and those with mental health conditions. Sessions will range from 15 to 45 min. Telehealth sessions will be provided via the VSee platform (www.vsee.com). The active intervention uses personalised feedback, skill-building exercises and goal setting to help individuals reduce their symptoms of addictive eating and improve their dietary intake and relationship with food (see table 2 for overview of intervention sessions). The intervention is personalised based on an individual’s dominant trait/s associated with personality and risk of addictive behaviour (ie, the traits: depression proneness, anxiety proneness, sensation proneness and/or impulsivity proneness; measured via the Substance Use Risk Profile Scale,^37^ which the individual scores the most highly for) and addresses a range of factors that influence behaviour, both internal and external. Further, dominant trait/s associated with personality and risk of addictive behaviour are mapped to specific coping skill strategies, which are in turn incorporated into the goal-setting process. As part of session 1, the first 15 min of the consultation will be audio recorded to enable qualitative analysis of responses to two standardised questions regarding the elements of ‘control’ and ‘compulsion’ around the participant’s food intake. These two themes were previously identified, through thematic analysis of the feasibility study data,^66^ as having an influential relationship with addictive eating behaviours. On completion of the five telehealth sessions, participants will be invited to join a closed Facebook group from 3 months post-commencement of the intervention until the 6-month outcome survey measures are conducted. Joining the Facebook group is voluntary.

**Table 2:** Overview of intervention sessions

Session	Session aims
1. Personality (week 1: 45 min)	Introduce the intervention Determine participants’ main concerns with their food intake Provide feedback on baseline scores of addictive eating Discuss what this means when attempting and preparing to make changes Provide feedback on traits associated with personality and risk of addictive behaviour Discuss how personality traits may relate to food intake and addictive eating, and what this means for the individual Discuss coping strategies based on personality traits and complete ‘Urge Surfing’ activity Introduce ‘Distraction List’ Set homework task: choose and practice 2 coping strategy exercises Provide session summary
2. Food (week 2: 45 min)	Review session 1 Check in for episodes of overeating Discuss progress with homework task—coping strategies Provide feedback on dietary intake Discuss core versus non-core food intake (optional: discuss alcohol intake) Develop 3 nutrition goals using *SMARTER Goal Checklist* Positive—increase core foods Reduction—decrease non-core foods ‘Eating awareness’—using strategies to delay or halt overeating Discuss enablers/barriers when making changes to eating habits Discuss 'No Money No Time' website (www.nomoneynotime.com.au) Discuss ‘Practical Strategies to Achieve Goals’ Set homework task: complete ‘Triggers for Overeating Checklist’ Provide session summary
3. Skills (week 4: 30 min)	Review session 2 Assess progress with SMARTER goals Check in for episodes of overeating Discuss homework task—'Triggers for Overeating' Explore strategies to overcome triggers, building on previous coping strategies and ‘Practical Strategies to Achieve Goals’ Discuss and determine a ‘food line’ to identify when eating is no longer enjoyable or not tasting food Discuss strategies to stay below the ‘food line’ Set homework task: complete ‘Mood Monitor*’* worksheet Provide session summary
4. Confidence (week 8: 30 min)	Review session 3 Discuss progress with plan to stay below 'food line' and for episodes of overeating Explore enablers/barriers to achieving goals Discuss homework task—‘Mood Monitor’, and explore emotions that the participant has difficulty coping with Discuss seeing emotions differently Explore coping strategies for difficult emotions Discuss importance of sleep, physical activity and responsible intake of alcohol for emotional health Discuss implementing coping skills plan to achieve SMARTER goals (ie, consolidate information from sessions 1–4) Set homework task: practice implementation of coping skills plan to achieve goals Provide session summary
5. Moving forward (week 12: 20 min)	Review session 4 Check in/briefly problem solve and encourage participants to continue with goals and strategies Discuss topics from previous sessions (participant led) Reassess confidence to achieve goals Provide final *Addictive Eating Action Plan* Discuss how support group on Facebook works and encourage sign up

#### Participant workbook and programme website

Participants will have access to a participant workbook and password-protected access to a study-specific website, both built for the study to support the materials discussed in the intervention sessions. To further facilitate the co-design process, the workbook and website content were piloted with end users (n=2) with lived experience of addictive eating, who participated in the iKT interviews/workshops. The end users reported the workbook and website to be highly usable in terms of the content, and the language used throughout as appropriate with only minor modifications made. Additionally, the piloting process allowed the estimated time to complete each workbook module to be calculated.

#### Programme workbook

The workbook consists of five modules: (1) personality; (2) food; (3) skills; (4) confidence and (5) moving forward. The content of the five modules mirrors that of the telehealth sessions. The workbook also contains reflective activities/worksheets, discussed during the telehealth sessions, for the participants to complete. These elements were deemed important during the iKT process. The amount of time spent completing activities in the workbook each week, between telehealth sessions, will take approximately 30–60 min. However, the time to complete each module may vary from person to person, and participants are advised to work through the workbook at a pace that is right for them.

#### Programme website

The website includes the following pages: (1) home/landing page: brief information about the programme and login; (2) dashboard: navigation page to access each of the programme’s module pages; (3) module pages: each of the five modules within the intervention has a separate page on the website—this includes additional resources to complement the telehealth sessions and workbook; and (4) about us: brief information about the research/clinician team behind the programme, including contact information (email). The website will be available for a period of 12 months from study commencement. All data captured from the website will be encrypted and stored securely on a server.

#### Programme Facebook group

This is a voluntary part of the study, which aims to further support participants with behaviour change. The Facebook forum is set up as a private Facebook group. Participants can use their standard Facebook login, or alternatively, create a new login (a pseudo-account) that does not identify them if they wish to remain anonymous. Participants will be prompted with information related to the intervention for the 3-month duration in the form of short posts, blogs and polls. The Facebook group will allow participants to engage with other participants from the programme, as well as serve as a communication method to remind participants about assessments for the study.

The Facebook forum has the following restrictions: (1) membership will be by invitation only; (2) the group will not appear in search results or the participant’s Facebook profile; and (3) only group members will be able to see the group information and group posts. Participants will be advised of the appropriate use of language and etiquette for using the social media/discussion forum in the workbook and reminded at the final telehealth session. The Facebook group will be moderated by a member of the research team via the TRACE research Facebook account.

#### Intervention fidelity

A detailed clinician manual will be used by the dietitian for all telehealth sessions to maintain treatment fidelity. Dietitians administering the intervention will be trained by the principal investigator prior to study implementation. Dietitians will also follow each session as outlined in the manual and keep a dietitian log of participants’ telehealth sessions. Further, five participants allocated to group 1, with their consent, will have all their telehealth sessions audio recorded. The dietitian log and audio recordings will be reviewed by an independent researcher to ensure the intervention was delivered as intended. Regular supervisory meetings will be conducted with the dietitians and study coordinator led by the principal investigator. Participant adherence to the intervention will be assessed by a session attendance checklist completed by a member of the research team. Dietitians administering the telehealth sessions will monitor completion of homework tasks and workbook activities at the start of telehealth sessions 2–5. Assistance will be provided by the dietitian at this time if participants experienced any difficulties completing the homework tasks/activities. Additionally, to assist with adherence, on completion of each telehealth session, the dietitian will email a personalised ‘Addictive Eating Action Plan’, completed on a standardised template, to the participant.

### TRACE passive intervention (group 2)

Participants will receive the intervention via self-guided approach, with access to the five-module workbook and website (as described above), but without the telehealth consults. The content of the workbook modules mirrors the content of the five telehealth sessions. In addition to the written materials provided, the workbook contains spaces for reflective activities, documenting goals and monitoring progress. Participants will be asked, on receipt of the workbook, to complete the workbook within a 3-month period. The proportion of the workbook completed by participants in the passive intervention arm will not be monitored. Following the 3-month self-guided learning period, participants will be invited to join the closed Facebook group as described above.

### Control (group 3)

Participants will receive personalised dietary feedback on baseline surveys, provided by an automated report, generated from the Australian Eating Survey. This is consistent with standard dietary feedback from a dietitian. Participants in the control group will be offered access to the participant workbook and study website after the completion of the 6-month assessment.

### Patient and public involvement

Consumer (ie, individuals with lived experience of addictive eating who participated in the pilot study) input was received on the pilot version of the intervention (FoodFix process evaluation^24^) that directly guided the enhancement of the TRACE telehealth sessions. The TRACE Programme workbook and website for the current study were developed following the pilot study. A sample of consumer representatives (individuals with lived experience of addictive eating and healthcare experts including clinicians and managers), independent of those involved in the pilot study, were involved in the review of the programme and programme materials.^49^

Consumer representatives were interviewed to:

- Identify what individuals with addictive eating need and want more accurately.
- Gather information about what works well and what needs improving, first hand from consumers who may use them.
- Openly consider different or opposing views about aspects of the research project.
- Test resources during development and refine resources making sure they will work well in practice.
- Detect any unforeseen consequences of a particular decision or direction that has been made regarding the project.
- Gain support of consumers to implement changes to the research project.

The opinion of consumers has been considered to create a programme that:

- Aligns to the needs of the people it is trying to help, that is, individuals with addictive eating.
- Is beneficial in terms of delivering meaningful outcomes for individuals with addictive eating.
- Is conducted in a way that is sensitive to people’s needs.

Consumers were not involved in the design of the current study, the selection of outcome measurements, research questions or the recruitment of additional participants. However, consumers were involved in the overall concepts employed in the study and may be called upon at the dissemination stage. For example, to review plain language summaries of the results, provide advice on ways to communicate/translate our findings or present our findings to the community. Participants of the current study can request a plain English summary of the study outcomes on its completion.

### Outcome measures

All outcome measures are completed at baseline, 3 months (immediate post-active intervention period) and 6 months (follow-up) via online surveys. The same survey tools will be used at each time point. Participants will receive assessment reminders by email (reference to where data collection forms can be found is included in online supplemental material 2).

10.1136/bmjopen-2022-064151.supp2 Supplementary data

### Primary outcomes

#### Addictive eating symptoms and severity

The YFAS 2.0^6^ will be used to assess the change in addictive eating symptomatology and severity. The YFAS 2.0 is a validated self-report 35-item questionnaire. The YFAS 2.0 asks participants to think of specific foods they have had difficulty controlling the consumption of within the past 3 months (eg, ice cream, chocolate, chips, hamburgers). The YFAS 2.0 provides an addictive eating symptom score ranging from 0 to 11. Additionally, two items assess clinically significant impairment or distress from eating. A ‘food addiction diagnosis’ can be given when ≥2 symptoms are endorsed, and clinically significant impairment or distress is present. However, for the purpose of this study, a ‘food addiction diagnosis’ will not be given, and severity of addictive eating will be classified in accordance with YFAS scoring instructions as follows: ‘mild’=3 symptoms, ‘moderate’=4–5 symptoms or ‘severe’=≥6 symptoms. The YFAS 2.0 has been found to be a robust and psychometrically sound measure of addictive eating symptomatology in non-clinical^2 67^ and clinical populations with good test/retest validity.^68^ Preliminary evidence^23 24^ suggests that YFAS scores are sensitive to change and are decreased after intervention.

### Secondary outcomes

#### Dietary intake and quality

Changes in dietary intake and quality will be measured using the Australian Eating Survey (AES).^69^ The following dietary outcomes will be measured: (1) core food and non-core food percentage contribution to total energy intake; (2) average daily energy intake, proportion of total energy intake contributed by macronutrient and micronutrient intake; and (3) overall diet quality. The AES is a validated 120-item semiquantitative Food Frequency Questionnaire that assesses usual food and nutrient intake over the previous 3–6 months. The AES includes a comprehensive list of foods, including drinks, milk and dairy foods, breads and cereals, sweet and savoury snacks, main meals, other foods, vegetables and fruits. Frequency response options for each food, or food type, range from ‘never’ to ‘≥7 times per day’. The AES has been assessed for comparative validity relative to weighed food records and for fruit and vegetable intake using plasma carotenoids.^69 70^ Standard portion sizes for adult men and women have been determined for each AES item in the survey, using data from the most recent Australian National Nutrition Survey. The food and beverage weight per serving, used in the calculation of food group servings (as serves per day), is consistent with sizes specified in the Australian Guide to Healthy Eating.^69–71^ Nutrient intake from the AES Food Frequency Questionnaire was computed using data in the AUSNUT 2011–2013 database.^72^ The AES also provides an Australian Recommended Food Score (ARFS), derived from a subset of 70 AES questions, as a measure of diet quality that reflects the overall healthiness and nutritional quality of an individual’s usual eating pattern.^70^ The ARFS is based on the frequency of consumption of core foods, recommended in the Australian Dietary Guidelines,^73^ with foods awarded 1 point for a consumption frequency of ≥once per week. The total score is calculated by summing the points for each item and scores can range from 0 to 73, with higher scores awarded for greater dietary variety.^70^

#### Depression, anxiety and stress

Changes in symptom scores for depression, anxiety and stress will be measured using the PHQ-8,^63^ the GAD-7^60^ and the Perceived Stress Scale (PSS-4),^61^ respectively. The PHQ-8 is a validated self-report eight-item tool that asks the individual to rate the severity of depressive symptoms over the past 2 weeks from 0 (‘not at all’) to 3 (‘nearly every day’). Total scores for the eight items range from 0 to 24, and severity will be determined using the following cut-offs: 0–4=minimal, 5–9=mild, 10–14=moderate, 15–19=moderately severe and 20–24=severe.^63^ The GAD-7 is a validated self-report seven-item tool that asks the individual to rate the severity of symptoms over the past 2 weeks from 0 (‘not at all sure’) to 3 (‘nearly every day’). GAD-7 total scores range from 0 to 21, and severity is determined using the following cut-offs: 0–5=mild, 6–10=moderate, 11–15=moderately severe and 16–21=severe.^60^ The PSS-4 is a validated self-report four-item tool that assesses the degree to which a person perceives life as stressful.^61^ The questions have been designed to assess how unpredictable, uncontrollable and overloaded a person feels their life to be. Frequency over the previous month is rated on a 5-point Likert scale ranging from ‘never’ to ‘very often’. PSS-4 total scores range from 0 to 16, and higher scores indicate greater stress.^61^ Currently, there is no established cut-off for the PSS-4 score to indicate adverse levels of stress.

### Other outcomes

A selection of other outcomes was chosen based on co-occurring health conditions (see table 1 for schedule of measurements).

#### Quality of life

Changes in subjective quality of life will be measured using the EQ-5D-5L.^74^ The EQ-5D-5L is a validated self-report five-item tool to assess health-related quality of life. It is a descriptive system comprising five dimensions: mobility, self-care, usual activities, pain/discomfort and anxiety/depression. Each dimension has three levels: no problems, some problems and extreme problems (labelled 1–3). Participants are asked to indicate their health state by ticking the box next to the most appropriate statement in each of the five dimensions. This decision results in a 1-digit number that expresses the level selected for that dimension. The digits for the five dimensions can be combined into a 5-digit number that describes the respondent’s health state. The EQ-5D-5L will be analysed to produce an index score between 0 (state of death) and 1 (perfect health).

#### Physical activity level

Changes in physical activity level will be measured using the Active Australia Survey (AAS).^75^ The AAS is a validated self-report tool containing eight core questions to assess participation (hours/min per week) in moderate and vigorous-intensity physical activity and walking for recreation, over the previous week.

#### Sleep hygiene behaviours

Changes in sleep hygiene behaviours will be measured using the Pittsburgh Sleep Quality Index (PSQI).^76^ The PSQI is a validated self-report survey with 19 self-rated items and 5 items rated by the bed partner or roommate (if applicable). The tool assesses seven components of sleep to provide one global score. Components measured include (1) subjective sleep quality, (2) sleep latency, (3) sleep duration, (4) habitual sleep, (5) sleep disturbances, (6) use of sleeping medication and (7) daytime dysfunction. The overall global score of sleep quality will be calculated and the subcomponents reported.

#### Healthcare utilisation

For the purpose of conducting a cost analysis, the Consumer Services Receipt Inventory (CSRI)^77^ will be completed by participants at each time point. The CSRI is an adaptable tool that ensures the format, language, scope and content are compatible with the research aims, context, participants’ likely circumstances, and the quantity and precision of information required.^78^ Healthcare utilisation is captured through self-report and includes information on the number of appointments and type of healthcare services used in the preceding 3 months.

#### Cost analysis

A cost–consequence analysis will be conducted including calculating the cost of each intervention (ie, active, passive and control) and reporting intervention costs alongside mean change outcomes. Intervention costs will be recorded in terms of cost of intervention development, intervention delivery and the operating costs of the randomised controlled trial. Outcomes to be reported as part of the cost analysis will include mean change in addictive eating symptom scores assessed using the YFAS (ie, the primary outcome), as well as mean change in the number of healthcare appointments in the past 3 months assessed using the CSRI, and mean change in quality-adjusted life years assessed using the EQ-5D-5L. This approach was selected to provide a comprehensive and transparent overview of intervention costs, given the lack of cost analysis data in this area of research.^79 80^

### Mediators/moderators

The following potential mediators and moderators of intervention efficacy will be examined:

#### Trait/s associated with personality and risk of addictive behaviour

Participants will complete the Substance Use Risk Profile Scale (SURPS)^81^ at baseline to determine their dominant trait/s. The SURPS is a validated self-report 23-item survey that assesses four traits associated with increased risk of addictive behaviours (impulsivity proneness, sensation proneness, depression proneness and anxiety proneness).

#### Eating behaviours

Eating behaviours that have been shown to have overlap with addictive eating will be measured. This includes eating disorders, binge eating, grazing behaviours and reward-driven eating. Eating disorders will be measured using the Eating Disorder Examination Questionnaire 6.0 (EDEQ-6.0)^62^ The EDEQ-6.0 is a validated self-report 28-item questionnaire that assesses the occurrence and frequencies of key eating disorder behaviours with cognitive subscales related to eating disorders (restraint, eating concern, shape concern and weight concern) and behavioural symptoms related to these concerns (eg, frequency of binge eating, vomiting, use of laxatives or diuretics, and overexercise). Subscale and global scores reflect the severity of eating disorder psychopathology. Binge eating will be measured using the Binge Eating Scale (BES).^82^ The BES is a validated self-report 16-item questionnaire to assess the presence of certain binge eating behaviours, over the past 28 days, which may be indicative of an eating disorder. Each item contains three to four statements about behaviours, thoughts and emotional states. Grazing behaviours will be measured using the Short Inventory of Grazing (SIG).^83^ The SIG is a validated self-report two-item measure to assess (1) the presence and frequency of grazing in general, and (2) the presence and frequency of grazing accompanied by a sense of loss of control. Reward-driven eating will be measured using the Reward-Based Eating Drive Scale (REDX-5).^84^ The REDX-5 is a validated self-report five-item questionnaire, in a 5-point Likert scale format from 1 (strongly disagree) to 5 (strongly agree), that assesses reward-driven eating (loss of control over eating, lack of satiety and preoccupation with food).

#### Participant activation level

Participants’ underlying knowledge, skills and confidence in managing their addictive eating behaviours and overall health will be measured using the Patient Activation Measure (PAM-13).^85^ The PAM-13 is a validated self-report 13-item scale that draws on concepts such as health locus of control, self-efficacy in managing health behaviours and readiness to change health behaviours.^64 86^ Higher PAM-13 scores indicate that individuals have higher levels of activation, and understand their role in the self-management process and feel capable of fulfilling that role.^87^ Research has demonstrated that a single-point change in PAM score is meaningful.^88^

### Engagement and use of the programme website and Facebook group

Interaction with the website will be objectively tracked throughout the study (baseline to 6 months, that is, time points 1–3) using Google Analytics (Google). Measures of engagement and usage will include number of website visits, website visit duration, number of page views and links accessed/resources downloaded.

Interaction with the Facebook group will be measured throughout the post-intervention period (3–6 months from baseline, that is, time points 2–3). Measures of engagement and usage will include number of participants to join the Facebook group, and number of views, likes and comments per post.

### Study sample characteristics

Sociodemographic data will be collected by an online questionnaire at baseline. Participants will provide information on their age, sex, marital status, postal code, years of education, employment status and current living situation. Index of Relative Socio-Economic Disadvantage score,^89^ based on the Australian Bureau of Statistics census data and reflecting a proxy index of socioeconomic status, will be determined by postal code of residence. Current smoking and substance use will be measured using the Alcohol, Smoking and Substance Involvement Screening Test V.3.0.^59^ Additionally, previous treatment sought for overeating from health professionals or products used to treat overeating will be collected.

Anthropometric data (self-reported height and weight) will be collected by online questionnaire at baseline. BMI will be calculated using standardised techniques and categorised according to the WHO adult cut-off points.^90^

### Sample size

The sample size for the study was calculated based on data from the feasibility study,^24^ given the absence of other intervention studies. Through guidance with statisticians, a large effect size was chosen and needed to enable the possibility of a clinically meaningful result. A clinically meaningful difference in symptoms of addictive eating was selected as a decrease of two symptoms, given this would correspond to a change in severity classification on the YFAS 2.0 tool. To detect a mean two-unit difference (SD=2.2) in the YFAS symptoms between the active intervention group and the passive intervention group or control group and using a standardised effect size of d=0.91, a sample size of 32 individuals per group (total sample size n=96) is required to detect this change with a power of 0.90 and a type 1 error rate set at 0.025 to account for multiple testing. However, allowing for a 30% dropout rate from the pilot, a sample size of 46 individuals per group (total sample size n=138) would be required. Therefore, a total sample size of 150 individuals, with 50 per group, was chosen to remain conservative.

### Statistical analysis plan

Data analysis will be conducted by a researcher blinded to the intervention conditions. Descriptive statistics of sample characteristics will be presented. For the primary YFAS outcome, a linear mixed model (LMM) will be based on a model with main effects for group (active intervention, passive intervention, control) and time (treated as categorical at levels baseline, 3 and 6 months), and the group-by-time interaction. An unstructured residual covariance structure will be used to allow for correlation between the repeated measurements for a subject. The primary outcome effect will be reported as the difference between means at baseline and 3 months, with a 95% CI for the difference. Mental health condition and BMI will be examined for possible moderating effects on the effect size, and if so, adjustment for them will be carried out. Secondary descriptive analysis will be carried out to identify whether specific symptoms were predominantly associated with reductions in YFAS score.

A secondary outcome will be a categorical variable, clinically significant change from baseline to 3 months, where significant requires a reduction of two or more symptoms in the YFAS. This will be analysed using logistic regression with group being the only factor. Additional secondary outcomes will include dietary outcomes (average daily energy intake, proportion of total energy intake contributed by core food and non-core food intake, macronutrient intake, micronutrient intake and overall diet quality) and mental health status (depression, anxiety and stress scores). These will also be analysed using LMMs as per the approach above. All available data will be used with no imputation of missing values at 3 and 6 months; however, baseline scores will be kept. The participants will be analysed according to their allocated randomisation group consistent with an intention-to-treat analysis. Statistical significance will be set at 0.05.

### Data management and monitoring

Online survey data will be managed using REDCap (Research Electronic Data Capture) electronic data capture tools^91 92^ hosted at the University of Newcastle. REDCap is a secure, web-based software platform designed to support data capture for research studies, providing (1) an intuitive interface for validated data capture; (2) audit trails for tracking data manipulation and export procedures; (3) automated export procedures for seamless data downloads to common statistical packages; and (4) procedures for data integration and interoperability with external sources.

All data captured from the study website will be encrypted and stored securely on the server. All other data collected will be entered into a password-protected central database, which is hosted on secure university-based servers, which comply with robust security standards for clinical data and are subject to daily backups and regular offsite backups. Only authorised members of the research team will have access to the database. Research staff handling study data are trained in procedures for handling sensitive information, accurate data entry, surveillance and intervention-specific data storage and data archive. Facilitators of the telehealth sessions are responsible for the electronic storage of study forms on the central database. All completed forms will be checked for completeness and accuracy, first by data collectors and later by a member of the research team responsible for data management. Throughout the study period (at 25% and 50% of required participants), approximately 5% of records will be randomly selected for data quality checks of accuracy and completeness by an independent reviewer.

A Data Safety Monitoring Board will not be established for this study as all elements of the intervention have been previously explored and used in interventions. To monitor for potential risks, the study coordinator managing the day-to-day conduct of the trial, and facilitators of the telehealth sessions, will report weekly to the chief investigator. Oversight concerning the overall conduct of the trial will be provided by our multidisciplinary research team. This will include regular meetings to review protocol adherence, participant retention rates and safety reports. For the entire study period, any adverse events, of any kind, that might be related to either the trial intervention or trial procedures will be logged in an adverse event log and reported to the Human Research Ethics Committee by the chief investigator. To maintain the welfare of participants, with their consent, relevant survey results from the GAD-7^60^ and PHQ-8^63^ will be sent to the participant’s nominated general practitioner/health professional if they score in the severe category for either anxiety (GAD-7 scores ≥16) or depression (PHQ-8 scores ≥20), if participants consent to this disclosure.

### Study sponsorship and organisation

The sponsor of the trial is the University of Newcastle, and funding was provided by the National Health and Medical Research Council. The trial will be conducted and evaluated independent of the study sponsor and funder. The study is coordinated independently of the study sponsor and funder, by researchers at the University of Newcastle, Australia with the study overseen by the trial management committee comprising the chief investigators.

## Ethics and dissemination

The trial will be undertaken in compliance with the Declaration of Helsinki and approval to conduct the study was received from the University of Newcastle Human Research Ethics Committee (H-2021-0100). This trial adheres to the SPIRIT guidelines for randomised trial protocols^51^ and the results will be reported in accordance with CONSORT guidelines (TIDieR checklist and guide^50^). Protocol modifications will be registered with the Ethics Committee and trial register. All participants will provide electronic consent to participate prior to completing the eligibility and baseline surveys. Results of the study will be disseminated via peer-reviewed publications and presentations at national and international conferences and will also form part of student dissertations. Data from the TRACE Study may be made available in the future for collaborative research questions. Such requests must be authorised by the principal investigators and the appropriate Human Research Ethics Committees.

### Limitations

Limitations of the study include the level of experience required of the dietitians administering the telehealth sessions, which may impact the scalability of the intervention. However, dietitians are highly trained professionals in behaviour change and extra care was taken given the uniqueness of the intervention. The fidelity outcomes assessed as part of the trial will provide important information regarding future implementation. Additional limitations include the exclusion of individuals with severe mental illnesses or complex health conditions. The current intervention is not designed for complex comorbidities. It is envisaged that for these individuals, a more complex care model is required where the TRACE Programme could be implemented alongside other approaches or treatments.

Currently, there are few published interventions run by dietitians and/or other health clinicians for addictive eating demonstrating the clear need for services and trialling of interventions that may be effective at facilitating changes in addictive eating behaviours. The TRACE Programme was designed to raise awareness and support behaviour change of addictive eating. If successful, our study will provide essential evidence regarding the efficacy of behavioural and dietary improvement in the management of addictive eating, thus allowing for the implementation of management strategies for addictive eating into community and clinical healthcare services. Further, if both the active and passive interventions are found to be effective, it will provide evidence of different levels of care that could be used within these services.

## Supplementary Material

Reviewer comments

Author's manuscript
