# Supplementary material for: Examining the efficacy of a telehealth intervention targeting addictive eating in Australian adults (the TRACE Programme): a randomised controlled trial protocol
Source: BMJ Open. 2023 Jun 6;13(6):e064151. doi: 10.1136/bmjopen-2022-064151 (PMC10255192; doi:10.1136/bmjopen-2022-064151)
Supplement: Supplementary data [file bmjopen-2022-064151supp001.pdf]

WELCOME BACK!As you have been found eligible to participate in the personality based intervention for addictive eating, it is important that you carefullly read the following information and give consent at the bottom if you wish to continue to the baseline surveys.

I have read the previous Participant Information Statement and give my consent to participate in this study?	◯ Yes<br>◯ No
I understand that it is possible that some questionnaires may identify potential health issues that may require follow-up with my GP. I give consent for a copy of the relevant results to be sent to my local doctor/GP or other Health Professional	◯ Yes<br>◯ No
Please provide Health Professional details (e.g Name, Phone number)	________________________________

I agree to participate in the Personality based intervention for Addictive Eating Behaviours study and give my consent freely.

I understand that the project will be conducted as described in the previous Participant Information Statement, a copy of which I have had the opportunity to download.

I understand I can withdraw from the project at any time and do not have to give any reason for withdrawing.
I am aware I have an equal chance of being allocated into one of three intervention groups. If allocated to:

• Group 1, I consent to completing online questionnaires at the beginning of the study and after 3 and 6 months, and to participating in five telehealth/phone consultations of 30-45mins with an Accredited Practising Dietitian.

• Group 2, I consent to completing online questionnaires at the beginning of the study and after 3 and 6 months, and to complete the self-guided workbook and access the study website.

• Group 3, I consent to completing online questionnaires at the beginning of the study and after 3 and 6 months, and to follow my usual dietary intake for the study duration. I understand that after 6 months I will have access to complete the self-guided workbook and access to the study website.

If allocated to group 1, I consent for my five sessions with the dietitian to be recorded for quality and training purposes.	◯ Yes<br>◯ No
Please provide your First Name:	__________________________
Please provide your Last Name:	__________________________
Please sign:	________________________________
Do you wish to continue to the Baseline Surveys?	◯ Yes<br>◯ No

Skinner JA, *et al. BMJ Open* 2023; 13:e064151. doi: 10.1136/bmjopen-2022-064151
