# Supplementary material for: Examining the efficacy of a telehealth intervention targeting addictive eating in Australian adults (the TRACE Programme): a randomised controlled trial protocol
Source: BMJ Open. 2023 Jun 6;13(6):e064151. doi: 10.1136/bmjopen-2022-064151 (PMC10255192; doi:10.1136/bmjopen-2022-064151)
Supplement: Supplementary data [file bmjopen-2022-064151supp002.pdf]

Title: Examining the efficacy of a telehealth intervention targeting addictive eating in Australian adults (the TRACE program): a randomised controlled trial protocol

Reference to where data collection forms can be found

Survey	Assessment tool	Reference	Available from
**AAS**	**Active Australia Survey**	Australian Institute of Health and Welfare (AIHW) 2003. The Active Australia Survey: a guide and manual for implementation, analysis and reporting. Canberra: AIHW.	https://www.aihw.gov.au/reports/physical-activity/active-australia-survey/summary
**AES**	**Australian Eating Survey**	Ashton L, Williams R, Wood L, Schumacher T, Burrows T, Rollo M, et al. Comparison of Australian Recommended Food Score (ARFS) and Plasma Carotenoid Concentrations: A Validation Study in Adults. Nutrients. 2017;9(8):888. http://doi.org/10.3390/nu9080888<br><br>Collins CE, Boggess MM, Watson JF, Guest M, Duncanson K, Pezdirc K, et al. Reproducibility and comparative validity of a food frequency questionnaire for Australian adults. Clinical Nutrition. 2014;33(5):906-14. doi: 10.1016/j.clnu.2013.09.015	https://australianeatingsurvey.com.au/
**ASSIST**	**Alcohol, Smoking and Substance Involvement Screening Test - Version 3.0**	WHO ASSIST Working Group (2002) The Alcohol, Smoking and Substance Involvement Screening Test (ASSIST): development, reliability and feasibility. Addiction, 97:1183-1194. doi: 10.1046/j.1360-0443.2002.00185.x	https://www.who.int/publications/i/item/978924159938-2
**BES**	**Binge Eating Scale**	Gormally J, Black S, Daston S, Rardin D. The assessment of binge eating severity among obese persons. Addictive Behaviors. 1982;7(1):47-55. https://doi.org/10.1016/0306-4603(82)90024-7	Available in the publication
**CSRI**	**Consumer Services Receipt Inventory**	Beecham J and Knapp M. (2001) Costing psychiatric interventions, in G. Thornicroft (ed.) Measuring Mental Health Needs, Gaskell, 2nd edition, 200-224.	https://www.pssru.ac.uk/csri/what-is-the-csri/
**EDE-Q 6.0**	**Eating Disorder**	Fairburn C, Cooper Z, O'Connor M. Eating disorders examination (16.0D) In: Fairburn C (Ed.), editor. In:	https://nedc.com.au/assets/Medicare-related-forms/Eating-Disorder-Examination-Questionaire-Smart-PDF.pdf

	**Examination Questionnaire 6.0**	Cognitive behavior therapy and eating disorders. New York: Guilford Press; 2008.	
**EDE-QS**	**Eating Disorder Examination Questionnaire Short Form**	Prnjak K, Mitchison D, Griffiths S, Mond J, Gideon N, Serpell L, Hay P. Further development of the 12-item EDE-QS: identifying a cut-off for screening purposes. BMC Psychiatry. 2020;20:146. https://doi.org/10.1186/s12888-020-02565-5	Available as Supporting Information accompanying the publication
**EQ5D-5L**	**EQ5D-5L**	Brazier J, Ratcliffe J, Tsuchiya A, Salomon J. Measuring and Valuing Health Benefits for Economic Evaluation. 2nd ed. Oxford: Oxford University Press; 2016. doi: 10.1093/med/9780198725923.001.0001.	https://aci.health.nsw.gov.au/__data/assets/pdf_file/0003/632847/EuroQol-5-Dimension.pdf
**GAD-7**	**Generalized Anxiety Disorder 7**	Spitzer RL, Kroenke K, Williams JBW, Löwe B. A Brief Measure for Assessing Generalized Anxiety Disorder (The GAD-7). Archives of Internal Medicine. 2006;166(10):1092–7 https://doi.org/10.1001/archinte.166.10.1092	https://adaa.org/sites/default/files/GAD-7_Anxiety-updated_0.pdf
**PAM-13**	**Patient Activation Measure 13-item**	Hibbard JH, Stockard J, Mahoney ER, Tusler M. (2004). Development of the Patient Activation Measure (PAM): conceptualizing and measuring activation in patients and consumers. Health services research. 2004;39(4 Pt 1): 1005–1026. https://doi.org/10.1111/j.1475-6773.2004.00269.x	https://www.insigniahealth.com/products/pam
**PQH-8**	**Patient Health Questionnaire**	Kroenke K, et al., The PHQ-8 as a measure of current depression in the general population. Journal of affective disorders, 2009. 114(1-3): p. 163-173 https://doi.org/10.1016/j.jad.2008.06.026	https://www.psychologywizard.net/uploads/2/6/6/4/26640833/kroenke_phq8.pdf
**PSQI**	**Pittsburgh Sleep Quality Index**	Buysse DJ, Reynolds CF 3rd, Monk TH, Berman SR, Kupfer DJ. The Pittsburgh Sleep Quality Index: a new instrument for psychiatric practice and research. Psychiatry Research. 1989;28(2): 193–213. https://doi.org/10.1016/0165-1781(89)90047-4	https://www.med.upenn.edu/cbti/assets/user-content/documents/Pittsburgh%20Sleep%20Quality%20Index%20(PSQI).pdf
**PSS-4**	**Perceived Stress Scale**	Ingram PB 4th, Clarke E, Lichtenberg JW. Confirmatory Factor Analysis of the Perceived Stress Scale-4 in a Community Sample. Stress Health. 2016; 32(2): 173–176. https://doi.org/10.1002/smi.2592	https://scholar.harvard.edu/files/bettina.hoeppner/files/pss-4.pdf

**REDX-5**	**Reward-Based Eating Drive Scale**	Vainik U, Han C, Epel ES, Dagher A, Mason AE. Rapid assessment of reward-related eating: The RED-X5. Obesity. 2019;27(2):325–31. doi:10.1002/oby.22374	https://www.ncbi.nlm.nih.gov/pmc/articles/PMC6352904/
**SURPS**	**Substance Use Risk Profile Scale**	Woicik PA, Stewart SH, Pihl RO, Conrod PJ. The Substance Use Risk Profile Scale: a scale measuring traits linked to reinforcement-specific substance use profiles. Addictive Behaviors. 2009;34(12): 1042-55. doi: 10.1016/j.addbeh.2009.07.001	Available on request from the corresponding author:<br>Woicik can be contacted at Neuropsychoimaging Group, Brookhaven National Laboratory, Medical Department, Building 490, Upton, New York, 11973, United States. Tel.: +1 631 344 4472. Conrod, NIHR Biomedical Research Centre, Section of Addiction, Department of Psychological Medicine and Psychiatry, King's College London, 4 Windsor Walk, Denmark Hill, London, SE5 8BB, United Kingdom. Tel.: +44 207 848 0836; fax: +44 207 701 8584.p.conrod@iop.kcl.ac.uk
**YFAS 2.0**	**Yale Food Addiction Scale 2.0**	Gearhardt AN, Corbin WR, Brownell KD. Development of the Yale Food Addiction Scale Version 2.0. Psychology of Addictive Behaviors. 2016;30(1):113-21. doi: 10.1037/adb0000136	https://sites.lsa.umich.edu/fastlab/yale-food-addiction-scale/
