# Supplementary material for: Examining the efficacy of a telehealth intervention targeting addictive eating in Australian adults (the TRACE Programme): a randomised controlled trial protocol
Source: BMJ Open. 2023 Jun 6;13(6):e064151. doi: 10.1136/bmjopen-2022-064151 (PMC10255192; doi:10.1136/bmjopen-2022-064151)
Supplement: Author's manuscript [file bmjopen-2022-064151.draft_revisions.pdf]

BMJ Open

BMJ Open is committed to open peer review. As part of this commitment we make the peer review history of every article we publish publicly available.

When an article is published we post the peer reviewers' comments and the authors' responses online. We also post the versions of the paper that were used during peer review. These are the versions that the peer review comments apply to.

The versions of the paper that follow are the versions that were submitted during the peer review process. They are not the versions of record or the final published versions. They should not be cited or distributed as the published version of this manuscript.

BMJ Open is an open access journal and the full, final, typeset and author-corrected version of record of the manuscript is available on our site with no access controls, subscription charges or pay-per-view fees (http://bmjopen.bmj.com).

If you have any questions on BMJ Open's open peer review process please email info.bmjopen@bmj.com

BMJ Open

Examining the efficacy of a telehealth intervention targeting addictive eating in Australian adults (the TRACE program): a randomised controlled trial protocol

Journal:	*BMJ Open*
Manuscript ID	bmjopen-2022-064151
Article Type:	Protocol
Date Submitted by the Author:	25-Apr-2022
Complete List of Authors:	Skinner, Janelle; The University of Newcastle, School of Health<br>Whatnall, Megan; The University of Newcastle, School of Health<br>Leary, Mark; The University of Newcastle,<br>Collins, Rebecca; The University of Newcastle, School of Health<br>Pursey, Kirrilly; The University of Newcastle, School of Health<br>Verdejo-García, Antonio; Monash University<br>Hay, Phillipa ; Western Sydney University<br>Baker, Amanda; The University of Newcastle,<br>Hides, Leanne; University of Queensland,<br>Paxton, Susan; La Trobe University<br>Wood, Lisa; The University of Newcastle, Respiratory Medicine<br>Colyvas, Kim; The University of Newcastle, School of Mathematical and Physical Sciences<br>Collins, Clare; The University of Newcastle<br>Burrows, Tracy; The University of Newcastle,
Keywords:	NUTRITION & DIETETICS, PUBLIC HEALTH, Eating disorders < PSYCHIATRY

SCHOLARONE™
Manuscripts

Article type: Protocol

Title: Examining the efficacy of a telehealth intervention targeting addictive eating in Australian adults (the TRACE program): a randomised controlled trial protocol

Authors: Janelle A Skinner[1,2], Megan Whatnall[1,2], Mark Leary[1,2], Rebecca A Collins[1,2], Kirrilly M Pursey[1,2], Antonio Verdejo-Garcia[3], Phillipa J Hay[4], Amanda L Baker[2,5], Leanne Hides[6], Susan J Paxton[7], Lisa G Wood[2,8], Kim Colyvas[1], Clare E Collins[1,2], Tracy L Burrows[1,2*]

Institutions:

[1]School of Health Sciences, College of Health, Medicine and Wellbeing, University of Newcastle, Callaghan NSW 2308, Australia

[2]Hunter Medical Research Institute, University of Newcastle, New Lambton Heights, NSW, 2305, Australia

[3]School of Psychological Sciences and Turner Institute for Brain and Mental Health, Monash University, Clayton, VIC 3800, Australia

[4]Translational Health Research Institute, Western Sydney University, Campbelltown, NSW, 2751, Australia

[5]School of Medicine and Public Health, College of Health, Medicine and Wellbeing, University of Newcastle, Callaghan, NSW 2308, Australia

[6]School of Psychology, University of Queensland, St Lucia, QLD, 4072, Australia

[7]Department of Psychology and Counselling, Latrobe University, Melbourne, VIC, 3086

[8]School of Biomedical Sciences and Pharmacy, College of Health, Medicine and Wellbeing, University of Newcastle, Callaghan NSW 2308, Australia

***Corresponding Author:** Professor Tracy Burrows. Mailing address: School of Health Sciences, University Drive, University of Newcastle, Callaghan, NSW, Australia, 2308. Ph: +610249215514. Email: tracy.burrows@newcastle.edu.au

Word count: 7344

Key words: addictive eating, food addiction, Yale Food Addiction Scale

For peer review only - http://bmjopen.bmj.com/site/about/guidelines.xhtml

ARTICLE SUMMARY

Title: Examining the efficacy of a telehealth intervention targeting addictive eating in Australian adults (the TRACE program): a randomised controlled trial protocol

Abstract

Introduction *A*pproximately 15-20% of the adult population endorse symptoms of addictive eating and there are currently limited options for management. Motivational interviewing-based interventions, containing personality risk targeted coping skills training for addictive disorders, have been found to be effective for behaviour change. This project builds upon foundations of a feasibility study and co-design. **Methods and analysis** Using a three-arm randomised controlled trial design, this study aims to determine the efficacy of a telehealth intervention targeting addictive eating symptoms in Australian adults compared to passive intervention and control (no intervention) groups. Addictive eating symptoms are assessed using the Yale Food Addiction Scale (YFAS). Using a multicomponent clinician led approach, the active intervention consists of five telehealth sessions (15-45min each) delivered by a dietitian over 3 months. The intervention uses personalised feedback, skill-building exercises, reflective activities, and goal setting. Participants are provided with a workbook and web site access. The passive intervention group receive the intervention via a self-guided approach with access to the workbook and website (no telehealth). The control group will receive personalised written dietary feedback at baseline and participants advised to follow their usual dietary pattern for six months. The control group will be offered the passive intervention after 6-months. Other outcomes assessed will include dietary intake and quality, depression, anxiety and stress, quality of life, physical activity, and sleep hygiene. Data collection will occur at baseline (pre-intervention), 3 months (post-intervention) and 6 months. The primary endpoint is YFAS symptom scores at 3 months. A cost consequence analysis will determine intervention costs alongside mean change outcomes. **Ethics and dissemination** The Human Research Ethics Committee of The University of Newcastle Australia provided approval: H-

For peer review only - http://bmjopen.bmj.com/site/about/guidelines.xhtml

2021-0100. Findings will be disseminated via publication in peer-reviewed journals, conference presentations, community presentations and student theses. Trial registration: Australia New Zealand Clinical Trial Registry (ANZCTR) ACTRN12619001540101.

Strengths and Limitations of this study

- Targeting of personality risk factors through tailoring of coping strategies and use of motivational interviewing to improve symptoms of addictive eating
- Co-design approach taken, with both consumers and a range of health professionals, to develop a program that is relevant and acceptable to end-users
- Detailed assessment of eating behaviours, mental health and lifestyle factors provides the opportunity for personalised feedback to examine how these behaviours change during the intervention
- Fidelity outcomes assessed and cost consequence analysis will provide important information regarding future implementation
- Limitations include participants being excluded with severe mental illnesses or complex health conditions

For peer review only - http://bmjopen.bmj.com/site/about/guidelines.xhtml

INTRODUCTION

Research in addictive eating has increased rapidly in recent years. Although the construct remains contentious in the scientific community,[1] addictive eating or 'food addiction' is accepted by consumers[2] and health practitioners.[3.4] Using self-report surveys, approximately 15-20% of the adult population endorse symptoms for addictive eating.[5,6] Higher prevalence rates have been reported in individuals with higher weight status.[6,7] Results from recent research indicate that individuals with addictive eating have significantly lower diet quality and higher intakes of highly processed foods.[8-10] There is also evidence that addictive eating commonly co-occurs with mental health co-morbidities, particularly depression and anxiety, as well as overlapping with eating disorders, specifically binge eating.[5, 11]

Current treatment options for addictive eating largely stem from online self-help groups such as Food Addicts Anonymous[12] and Overeaters Anonymous[13] which have 10 000+ members and have been in existence for many years, demonstrating a need for services.[14] A recent systematic review[15] found there is limited evidence supporting implementation of feasible and effective dietary interventions, run by clinicians, for the management of addictive eating.[16] Of the nine studies reviewed, five interventional studies were found to improve symptoms of food addiction[15] as indicated by improvements in Yale Food Addiction Scale (YFAS)[17, 18] outcomes. These interventions included medication (combination of naltrexone and bupropion,[19] as well as pexacerfont[20]), bariatric surgery[21, 22] and lifestyle modification.[23] However to date, most studies have been limited in sample size and therefore not been powered to detect a change in addictive eating symptoms.[15]

Motivational interviewing based interventions containing personality risk targeted coping skills training for addictive disorders, such as alcohol use, have been found to be effective.[24, 25] This project builds on a program of work that included an initial feasibility study of a targeted

For peer review only - http://bmjopen.bmj.com/site/about/guidelines.xhtml

personality-based intervention for the management of addictive eating in adults (Australia New Zealand Clinical Trial Registry ACTRN12619001540101).[16] Results from the initial study indicated that the program was feasible in the target population. Feedback, received from program participants and facilitators, identified a need for a greater number of program sessions and improved strategies for increasing retention. As a result, the program was further refined with end users using an integrated knowledge translation (iKT) framework.[26] This co-design phase included consumers with lived experience, as well as health professionals from a range of disciplines to ensure the culmination of multidisciplinary evidence-based strategies were included. This was unique as previous reports omit this co-design step or are siloed in their approach.[26] The co-design process used a series of interviews and workshops to gain input into the program overview, aims, content and materials. Subsequent changes were made to the program content, language used, and materials were created or refined to improve acceptability.

Addictive eating is a complex issue often overlapping with other health conditions, and likely transdiagnostic. The resultant behaviour change intervention, the TRACE (Targeted Research on Addictive and Compulsive Eating) program, is a complex intervention and previously described using the Medical Research Council TiDier checklist for complex interventions.[27] The aim of the current study is to determine the efficacy of a telehealth intervention (active intervention) to reduce symptoms of addictive eating in adults, relative to passive intervention and control (no intervention) groups. It is hypothesised that both the active and passive intervention groups will achieve a statistically significant reduction in addictive eating symptoms relative to the control group. Potential moderators (e.g., participant sociodemographics) and mediators (e.g., physical activity, diet, and sleep) of intervention efficacy will also be evaluated.

For peer review only - http://bmjopen.bmj.com/site/about/guidelines.xhtml

METHODS

Study trial design

The TRACE program is a randomised controlled trial with three parallel arms (n=50 per group). The primary outcome is the change in addictive eating symptoms at the 3-month post-baseline assessment (primary time point). The study also includes a 6-month post-baseline follow-up assessment. In this study, symptoms of addictive eating are assessed using the Yale Food Addiction Scale 2.0 (YFAS).[18]

The intervention study arms are:

Group 1. Active intervention: targeting change in addictive eating behaviours using a multicomponent clinician led approach (telehealth sessions, program workbook and program website)

Group 2. Passive intervention: targeting change in addictive eating behaviours using a multicomponent self-guided approach (program workbook and program website)

Group 3. Control: dietary feedback, via paper-based report, provided at baseline and participants follow their usual dietary pattern for six months.

The comparator groups were chosen to provide a passive delivery option of the program which would be consistent with a self-guided Cognitive Behaviour Therapy approach (Group 2), and a control group consistent with a standard version of dietary feedback (Group 3). The control group is not a wait list control, however participants in this group will be offered access to the passive intervention (i.e., program workbook and program website) after the completion of the 6-month assessment.

This project was approved by The University of Newcastle Human Research Ethics Committee (H-2021-0100) and prospectively registered with the Australian New Zealand Clinical Trials Registry (ACTRN12621001079831). The study protocol was developed in accordance with the Standard Protocol Items: Recommendations for Interventional Trials (SPIRIT)

For peer review only

For peer review only - http://bmjopen.bmj.com/site/about/guidelines.xhtml

guidelines[28] and the intervention has been described using the TIDIER (Template for Intervention Description and Replication) checklist.[27] The design, conduct and reporting of the studies will adhere to the CONSORT (Consolidated Standards of Reporting Trials) guidelines.[29] All participants will provide informed electronic consent to participate and can withdraw at any time for any reason. The funding bodies had no role in the design, conduct or reporting of the study.

Setting

The target population for the TRACE program are adults (≥ 18 years) living in Australia seeking management of addictive eating, who meet eligibility criteria, assessed through an online screening questionnaire. The active intervention will be delivered via telehealth sessions and supported by a program workbook, and website containing materials relevant to the intervention.

Recruitment

Participants will be recruited using a range of strategies including media releases, advertising via local and national newspapers, and social-media releases. Informed by our iKT process, a range of recruitment videos (tailored for gender) were also created in addition to written material which will be released via Twitter and Facebook. Recruitment commenced in August 2021 and was completed in April 2022. Recruitment materials will direct individuals to the study information sheet and eligibility survey. The eligibility survey takes approximately 15 mins to complete (Table 1). Online informed consent will be obtained prior to completing the eligibility survey.

For peer review only - http://bmjopen.bmj.com/site/about/guidelines.xhtml

Table 1. Schedule of measurements for the parent study and the subgroup study

Variable	Instrument	Enrolment	Timepoint post allocation		
**Primary study**		Eligibility Screening	t_1 Baseline	t_2 3-months	t_3 6-months
**Sample characteristics**					
Demographics	Age, sex, postcode, mental health status	✓			
Socioeconomic factors	Education, income, marital status, employment status, occupation and living/accommodation status	✓			
Anthropometrics	Self-report height and weight	✓		✓	✓
Smoking and substance use	Alcohol, Smoking and Substance Involvement Screening Test - Version 3.0[1]		✓	✓	✓
Purging behaviours	Eating Disorder Examination Questionnaire Short form (EDE-QS)[2]	✓			
**Primary Outcomes**					
Food addiction symptoms and severity	Yale Food Addiction Scale 2.0[3]	✓		✓	✓
**Secondary Outcomes**					
Dietary intake and quality	Australian Eating Survey[4, 5]		✓	✓	✓
Depression, anxiety and stress	Patient Health Questionnaire-9,[6] Generalized Anxiety Disorder 7,[7] Perceived Stress Scale[8]	✓		✓	✓
**Mediators/moderators**					
Dominant personality trait/s	Substance Use Risk Profile Scale[9]		✓		
Eating Behaviours	Eating Disorder Examination Questionnaire 6.0,[10] Binge Eating Scale,[11] Short Inventory of Grazing,[12] Reward-Based Eating Drive Scale[13]		✓	✓	✓
Participant activation level	Patient Activation Measure 13[14]	✓		✓	✓
Usage and engagement with program website	Google Analytics (Google LLC) to record number of site visits, visit durations, number of page views, and links accessed/resources downloaded		◆	—	◆
Usage and engagement with Facebook group	Number of participants to join group; number of views, likes and comments per post manually recorded			◆	◆
**Other outcomes**					
Quality of life	EQ-5D-5L[15]		✓	✓	✓
Physical activity level	Active Australia Survey[16]		✓	✓	✓
Sleep hygiene behaviours	Pittsburgh Sleep Quality Index[17]		✓	✓	✓
Health care utilisation	Consumer Services Receipt Inventory[18]		✓	✓	✓
'Control' and 'Compulsion' associated with addictive eating	Qualitative analysis of a segment of the first telehealth session		✓		

Eligibility Screening = assessment of inclusion/exclusion criteria, Baseline = pre-intervention, 3-months = immediate post-intervention, 6-months = 3-months post-intervention.

For peer review only

For peer review only - http://bmjopen.bmj.com/site/about/guidelines.xhtml

Eligibility

To be eligible for inclusion in the study individuals must:

1. Be aged between 18 years and 85 years
2. Endorse ≥ 3 symptoms on the Yale Food Addiction Scale 2.0[18]
3. Have a self-reported weight and height consistent with a body mass index (BMI) ≥ 18.5 kg/m^2
4. Be competent in the English language
5. Live in Australia
6. Have access to the internet

Individuals will be excluded from participating in the study if they:

1. Are pregnant or lactating
2. Report having a severe mental illness (such as schizophrenia or bipolar disorder) or health condition
3. Report purging behaviours as identified by the Eating Disorder Examination Questionnaire – Short form (EDE-QS)[30]

Methodological considerations for eligibility criteria: The eligibility screener excludes individuals with a BMI below 18.5kg/m^2. This measure was put in place to reduce the likelihood of recruiting participants with at-risk restrictive eating practices that may be influencing a relatively low weight status. The value of <18.5 kg/m^2 was chosen as this is below the current healthy weight range in national guidelines for Australians[47] and The Centre of Disease Control and Prevention (CDC) in the USA.[48] Additionally, the eligibility screener includes the Eating Disorder Examination Questionnaire Short Form (EDE-QS).[31] This 12-item validated tool is commonly used to identify potential eating disorders. Based on the research team consensus, individuals who have compensatory behaviours such as bingeing/purging (specifically asked in question 7 on the EDE-QS), who may be at risk of an eating disorder and are medically compromised, will be deemed not eligible for the current study. Purging is

For peer review only

For peer review only - http://bmjopen.bmj.com/site/about/guidelines.xhtml

related to higher levels of appearance dissatisfaction, anxiety and depressive symptoms and self-concept instability.[49, 50] As per the ethics protocol, participants endorsing any response to this question, indicating these compensatory behaviours will be excluded from the study. The tools for eating disorders and psychological health[30,31,34-36,38] used in the study have been widely used in research in the areas of eating disorders, dietary interventions, substance use and mental health and are considered standard tools for their specific measures. Study information as well as at completion of surveys participants are provided with contact information if they experience or further assistance with health behaviours.

Study procedure

Prospective participants will complete the eligibility survey. This will include demographic questions (e.g., sex, postcode, marital status, level of education, employment status) the Yale Food Addiction Scale 2.0[18] to confirm endorsement of ≥ 3 addictive eating symptoms; the EDE-QS[31] to confirm the absence of purging behaviours; and the Patient Health Questionnaire-9 (PHQ-9),[34] Generalized Anxiety Disorder-7 (GAD-7),[35] and Perceived Stress Scale-4[36] to determine severity of depression, anxiety and stress, respectively. For participants scoring in the severe category for either depression (PHQ-9 scores of 16-20) or anxiety (GAD-7 scores of 15-21), with the participant's consent, copies of the relevant results will be sent to their nominated General Practitioner. While not necessary to determine eligibility, the Patient Activation Measure 13,[42] and two questions relating to previous treatments sought for addictive eating, will also be completed by potential participants. These questions have been specifically added to extend our previously reported research[51] regarding the types of individuals recruited into interventions for food addiction.

Participants deemed eligible will proceed to the online consent form (Figure 1. Overview of study schedule). Participants will be given a two-week period to consider participation. After this time, a member of the research team will contact any individuals via email who have not completed the consent form to determine their interest in participating. Following this, no other

For peer review only - http://bmjopen.bmj.com/site/about/guidelines.xhtml

contact will be made. Participants who provide electronic written consent will complete the baseline assessment surveys measuring dietary intake and eating habits, personality, quality of life and healthcare service utilisation (Table 1. Schedule of measurements). The surveys take approximately 40 minutes to complete. On completion of baseline surveys, participants will be randomly allocated to one of three groups (Group 1: active intervention; Group 2: passive intervention; or Group 3: control; see *Intervention* description) and informed of their group allocation via email.

Figure 1. Overview of the study schedule

Following randomisation (see *Randomisation and Blinding*), a member of the research team will contact participants in Group 1 via telephone or email to arrange an appointment time for their initial telehealth session. Groups 1 and 2 will be emailed a copy of the program workbook (printable and fillable PDF versions); a hard copy is available for participants on request; and be provided with password protected access to the program website at this time. Telehealth sessions 2 – 5, for participants allocated to the active intervention group (Group 1), will be arranged during their first telehealth session.

Participants from all three groups will receive results from the eligibility and/or baseline surveys by the research team via email. On survey completion, Groups 1 and 2 will receive feedback on dominant personality trait/s that may be associated with increased risk for addictive behaviours (e.g., anxiety-proneness, impulsivity-proneness); symptoms of addictive eating; dietary, caffeine and alcohol intake; sleep hygiene and physical activity levels. At this timepoint, Group 3 will only receive feedback on dietary intake via email. At 6-months post study commencement, Group 3 will be provided with feedback on personality trait/s; symptoms of addictive eating; sleep hygiene and physical activity levels, along with access to the workbook and website (the passive intervention that Group 2 received at baseline). To ensure consistency across participants, email templates and standardised reports will be used by the

For peer review only - http://bmjopen.bmj.com/site/about/guidelines.xhtml

research team. Group 2 will be guided with written instructions in their workbook on how to utilise their survey results to allow personalised goal setting regarding their dietary intake and eating patterns.

The primary and secondary outcomes will be assessed at 3-months (primary endpoint, immediate post-active intervention period) and 6-months (follow-up) where participants will complete post-program surveys (Table 1. Schedule of measurements). Participants will be sent reminder emails to complete their surveys. They will be reminded a maximum of three times at each time point. If no contact is received after such time, no further contact will be made. Participants will be remunerated with a gift voucher to the value of AUD20 at the completion of baseline, 3-month and 6-month surveys, corresponding to a maximum of AUD60 per participant over the course of the study.

Randomisation and Blinding

Following completion of baseline assessments, participants will be randomised to one of the three study groups in equal ratios using permuted block randomisation, with block sizes of six. Randomisation will be stratified by sex and mental health status (depression and anxiety) to ensure group balance on these important variables. The randomisation sequence will be generated by an independent statistician and implemented by a designated study co-ordinator. The allocation list will be stored in a password protected computer file and accessed only by the study co-ordinator.

Blinding of participants and dietitians to intervention group allocation in this study will not be possible. However, several strategies will be employed to reduce the risk of bias. First, participants will only be provided with partial information on the study hypotheses. Second, all communication between participants and research staff during the period of intervention (i.e., scheduling concerns, questions regarding the intervention) will be done directly between participants and the ‘study co-ordinator’ or their respective ‘telehealth clinician’. Lastly,

For peer review only

statistical analyses will be conducted by researchers who are blind to group allocation prior to analysis.

Intervention

TRACE Active Intervention (Group 1): Participants will receive five standardised one-on-one telehealth/phone sessions with an Accredited Practising Dietitian, with training in behaviour change and eating disorders, over a 3-month period (i.e., weeks 1, 2, 4, 8 and 12). Additionally, dietitians leading the intervention delivery will have extensive experience in private practice work and working with clients including those with disordered eating and those with mental health conditions. Sessions will range from 15-45 mins. Telehealth sessions will be provided via the VSee platform (www.vsee.com). The active intervention uses personalised feedback, skill-building exercises, and goal setting to help individuals reduce their symptoms of addictive eating and improve their dietary intake, and relationship with food (see Table 2 for *Overview of intervention sessions*). The intervention is personalised based on an individual's dominant personality trait/s (i.e., the traits; depression proneness, anxiety proneness, sensation proneness and/or impulsivity proneness; measured via The Substance Use Risk Profile Scale[37] which the individual scores the most highly for) and addresses a range of factors that influence behaviour, both internal and external. Further, dominant personality trait/s are mapped to specific coping skill strategies which are in turn incorporated into the goal setting process. As part of session 1, the first 15 mins of the consultation will be audio recorded to enable qualitative analysis of responses to standardised questions regarding two elements of 'control' and 'compulsion' around the participant's food intake. These two themes were previously identified, through thematic analysis of the feasibility study data,[52] as having an influential relationship with addictive eating behaviours. On completion of the five telehealth sessions, participants will be invited to join a closed Facebook group from 3-months post commencement of the intervention until the 6-month outcome survey measures are conducted. Joining the Facebook group is voluntary.

For peer review only - http://bmjopen.bmj.com/site/about/guidelines.xhtml

Table 2. Overview of intervention sessions

Session	Session aims
**1) Personality (Week 1: 45 mins)**	• Introduce the intervention<br>• Determine participant's main concerns with their food intake<br>• Provide feedback on baseline scores of addictive eating<br>• Discuss what this means when attempting and preparing to make changes<br>• Provide feedback on dominant personality trait/s<br>• Discuss how personality traits may relate to food intake and addictive eating, and what this means for the individual<br>• Discuss coping strategies based on personality traits and complete '*Urge Surfing*' activity<br>• Introduce '*Distraction List*'<br>• Set homework task: choose and practice 2 coping strategy exercises<br>• Provide session summary
**2) Food (Week 2: 45 min)**	• Review session 1<br>• Check in for episodes of overeating<br>• Discuss progress with homework task - coping strategies<br>• Provide feedback on dietary intake<br>• Discuss core vs non-core food intake (Optional: discuss alcohol intake)<br>• Develop 3 nutrition goals using *SMARTER Goal Checklist*<br>1) Positive – increase core foods<br>2) Reduction – decrease non-core foods<br>3) 'Eating awareness' – using strategies to delay or halt overeating<br>• Discuss enablers/barriers when making changes to eating habits<br>• Discuss '*No Money No Time*' website (www.nomoneynotime.com.au)<br>• Discuss '*Practical Strategies to Achieve Goals*'<br>• Set homework task: complete '*Triggers for Overeating Checklist*'<br>• Provide session summary
**3) Skills (Week 4: 30 min)**	• Review session 2

For peer review only

Session	Session aims
	• Assess progress with SMARTER goals<br>• Check in for episodes of overeating<br>• Discuss homework task - '*Triggers for Overeating*'<br>• Explore strategies to overcome triggers, building on previous personality-based coping strategies and '*Practical Strategies to Achieve Goals*'<br>• Discuss and determine a '*food line*' to identify when eating is no longer enjoyable or not tasting food<br>• Discuss strategies to stay below the 'food line'<br>• Set homework task: complete '*Mood Monitor'* worksheet<br>• Provide session summary
**4) Confidence (Week 8: 30 min)**	• Review session 3<br>• Discuss progress with plan to stay below 'food line' and for episodes of overeating<br>• Explore enablers/barriers to achieving goals<br>• Discuss homework task - '*Mood Monitor*', and explore emotions that participant has difficulty coping with<br>• Discuss seeing emotions differently<br>• Explore coping strategies for difficult emotions<br>• Discuss importance of sleep, physical activity, and responsible intake of alcohol for emotional health<br>• Discuss implementing coping skills plan to achieve SMARTER goals (i.e. consolidate information from sessions 1 – 4)<br>• Set homework task: practice implementation of coping skills plan to achieve goals<br>• Provide session summary
**5) Moving forward (Week 12: 20 mins)**	• Review session 4<br>• Check in/briefly problem solve and encourage participant to continue with goals and strategies<br>• Discuss topics from previous sessions (participant led)<br>• Reassess confidence to achieve goals<br>• Provide final *Addictive Eating Action Plan*<br>• Discuss how support group on Facebook works and encourage sign up

345

For peer review only - http://bmjopen.bmj.com/site/about/guidelines.xhtml

Participant Workbook and Program Website: Participants will have access to a participant workbook and password protected access to a study specific website (www.tracedietetics.com.au), both built for the study to support the materials discussed in the intervention sessions. To further facilitate the co-design process, the workbook and website content was piloted with end users (n=2) with lived experience of addictive eating, who participated in the iKT interviews/workshops. The end users reported the workbook and website to be highly usable in terms of the content, and the language used throughout as appropriate with only minor modifications made. Additionally, the piloting process allowed the estimated time to complete each workbook module to be calculated.

Program Workbook: The workbook consists of five modules: 1) Personality; 2) Food; 3) Skills; 4) Confidence; and 5) Moving forward. The content of the five modules mirrors that of the telehealth sessions. The workbook also contains reflective activities/worksheets, discussed during the telehealth sessions, for the participants to complete. These elements were deemed important during the iKT process. The amount of time spent completing activities in the workbook each week, between telehealth sessions, will take approximately 30 - 60 minutes. However, the time to complete each module may vary from person to person, and participants are advised to work through the workbook at a pace that is right for them.

Program Website: The website includes the following pages: 1) Home/Landing page: brief information about the program and login; 2) Dashboard: navigation page to access each of the program's module pages; 3) Module pages: each of the five modules within the intervention has a separate page on the website. This includes additional resources to complement the telehealth sessions and workbook; and 4) About us: brief information about the research/clinician team behind the program, including contact information (email). The website will be available for a period of 12 months from study commencement. All data captured from the website will be encrypted and stored securely on a server.

For peer review only

Program Facebook Group: This is a voluntary part of the study which aims to further support participants with behaviour change. The Facebook forum is set up as a private Facebook group. Participants can use their standard Facebook login, or alternatively, create a new login (a pseudo account) that does not identify them if they wish to remain anonymous. Participants will be prompted with information related to the intervention for the 3-month duration in the form of short posts, blogs, and polls. The Facebook group will allow participants to engage with other participants from the program, as well as serve as a communication method to remind participants about assessments for the study.

To maintain participant's privacy the Facebook forum will be set up as a private Facebook group with the following restrictions: 1) Membership will be by invitation only; 2) The group will not appear in search results or the participants Facebook profile; and 3) Only group members will be able to see the group information and group posts. Participants will be advised of the appropriate use of language and etiquette for using the social media/discussion forum in the workbook and reminded at the final telehealth session. The Facebook group will be moderated by a member of the research team via the TRACE research Facebook account.

Intervention fidelity: A detailed clinician manual will be used by the dietitian for all telehealth sessions to maintain treatment fidelity. Dietitians administering the intervention will be trained by the principal investigator prior to study implementation. Dietitians will also follow each session as outlined in the manual and keep a dietitian log of participants telehealth sessions. Further, five participants allocated to Group 1, with their consent, will have all their telehealth sessions audio recorded. The dietitian log and audio recordings will be reviewed by an independent researcher to ensure the intervention was delivered as intended. Regular supervisory meetings will be conducted with the dietitians and program coordinator led by the principal investigator. Participant adherence to the intervention will be assessed by a session attendance checklist completed by a member of the research team. Dietitians administering the telehealth sessions will monitor completion of homework tasks and workbook activities at

the start of telehealth sessions 2 to 5. Assistance will be provided by the dietitian at this time if participants experienced any difficulties completing the homework tasks/activities. Additionally, to assist with adherence, on completion of each telehealth session the dietitian will email a personalised 'Addictive Eating Action Plan', completed on a standardised template, to the participant.

TRACE Passive Intervention (Group 2): Participants will receive the intervention via self-guided approach, with access to the five-module workbook and website (as described above), but without the telehealth consults. The content of the workbook modules mirrors the content of the five telehealth sessions. In addition to the written materials provided, the workbook contains spaces for reflective activities, documenting goals and monitoring progress. Participants will be asked, on receipt of the workbook, to complete the workbook within a 3-month period. The proportion of the workbook completed by participants in the passive intervention arm will not be monitored. Following the 3-month self-guided learning period, participants will be invited to join the closed Facebook group as described above.

Control (Group 3): Participants will receive personalised dietary feedback on baseline surveys, provided by an automated report, generated from the Australian Eating Survey. This is consistent with standard dietary feedback from a dietitian. Participants in the control group will be offered access to the participant workbook and study website after the completion of the 6-month assessment.

Outcome measures

All outcome measures are completed at baseline, 3 months (immediate post-active intervention period) and 6 months (follow-up) via online surveys. The same survey tools will be used at each time point. Participants will receive assessment reminders by email.

For peer review only - http://bmjopen.bmj.com/site/about/guidelines.xhtml

Primary outcomes

Food addiction symptoms and severity: The Yale Food Addiction Scale (YFAS 2.0)[18] will be used to assess the change in food addiction symptomatology and severity. The YFAS 2.0 is a validated self-report 35-item questionnaire. The YFAS 2.0 asks participants to think of specific foods they have had difficulty controlling the consumption of within the past 3 months (e.g., ice cream, chocolate, chips, hamburgers). The YFAS 2.0 provides a food addiction symptom score based on similar criteria for substance use disorder of the Diagnostic and Statistical Manual of Mental Disorders (DSM-5).[53] Scores can range from zero to 11, and symptoms include craving, loss of control, tolerance and withdrawal associated with eating behaviours. Additionally, two items assess clinically significant impairment or distress from eating. A food addiction "diagnosis" can be given when ≥2 symptoms are endorsed, and clinically significant impairment or distress is present. However, for the purpose of this study a 'food addiction' diagnosis will not be given, and severity of addictive eating will be classified as follows: "mild" = 3 symptoms, "moderate" = 4-5 symptoms or "severe" ≥6 symptoms. The YFAS 2.0 has been found to be a robust and psychometrically sound measure of food addiction symptomatology in non-clinical[17,54] and clinical populations with good test/retest validity.[55] Preliminary evidence[15,16] suggests that YFAS scores are sensitive to change and are decreased after intervention.

Secondary outcomes

Dietary intake and quality: Changes in dietary intake and quality will be measured using the Australian Eating Survey (AES).[32] The following dietary outcomes will be measured: (1) core foods and non-core foods percentage contribution to total energy intake; (2) average daily energy intake, proportion of total energy intake contributed by macronutrients, micronutrient intakes; and (3) overall diet quality. The AES is a validated 120-item semi-quantitative FFQ that assesses usual food and nutrient intakes over the previous 3-6 months. The AES includes a comprehensive list of foods, including drinks, milk and dairy foods, breads and cereals, sweet and savoury snacks, main meals, other foods, vegetables and fruit. Frequency

For peer review only - http://bmjopen.bmj.com/site/about/guidelines.xhtml

response options for each food, or food type, range from ‘never’ to ‘≥7 times per day’. The AES has been assessed for comparative validity relative to weighed food records and for fruit and vegetable intakes using plasma carotenoids.[32,33] Standard portion sizes for adult men and women have been determined for each AES item in the survey, using data from the most recent Australian National Nutrition Survey. The food and beverage weight per serving, used in the calculation of food group servings (as serves per day) is consistent with sizes specified in the Australian Guide to Healthy Eating.[32,33,56] Nutrient intakes from the AES FFQ were computed using data in the AUSNUT 2011–13 database.[57] The AES also provides an Australian Recommended Food Score (ARFS), derived from a subset of 70 AES questions, as a measure of diet quality that reflects the overall healthiness and nutritional quality of an individual's usual eating pattern.[33] The ARFS is based on the frequency of consumption of core foods, recommended in the Australian Dietary Guidelines,[58] with foods awarded one point for a consumption frequency of ≥once per week. The total score is calculated by summing the points for each item and scores can range from zero to 73, with higher scores awarded for greater dietary variety.[33]

Depression, anxiety and stress: Changes in symptom scores for depression, anxiety and stress will be measured using the Patient Health Questionnaire (PHQ-9),[34] the Generalized Anxiety Disorder 7 (GAD-7)[35] and the Perceived Stress Scale (PSS-4),[36] respectively. The PHQ-9 is a validated self-report 9-item tool that asks the individual to rate the severity of depressive symptoms over the past two weeks from 0 (‘not at all’) to 3 (‘nearly every day’). For this study, the question within the PQH-9 relating to suicide ideation was not included. Total scores for the remaining 8 items range from 0 to 24, and severity will be determined using the following cut-offs: 0-4 = minimal, 5-9 = mild, 10-14 = moderate, 15-19 = moderately severe, and 20-24 = severe.[34] The GAD-7 is a validated self-report 7-item tool that asks the individual to rate the severity of symptoms over the past two weeks from 0 (‘not at all sure’) to 3 (‘nearly every day’). GAD-7 total scores range from 0 to 21, and severity is determined using the following cut-offs: 0-5 = mild, 6-10 = moderate, 11-15 = moderately severe, and 15-21 =

For peer review only - http://bmjopen.bmj.com/site/about/guidelines.xhtml

severe.[35] The PSS-4 is a validated self-report 4-item tool that assesses the degree to which a person perceives life as stressful.[36] The questions have been designed to assess how unpredictable, uncontrollable, and overloaded a person feels their life to be. Frequency over the previous month is rated on a five-point Likert scale ranging from 'Never' to 'Very often'. PSS-4 total scores range from 0 to 16, and higher scores indicate greater stress.[36] Currently, there is no established cut-off for the PSS-4 score to indicate adverse levels of stress.

Other outcomes

A selection of other outcomes was chosen based on co-occurring health conditions. Outcomes collected (see Table 1 for schedule of measurements) include the following:

Quality of Life: Changes in subjective quality of life will be measured using the EQ-5D-5L.[43] The EQ5D-5L is a validated self-report 5-item tool to assess health-related quality of life. A descriptive system comprising five dimensions: mobility, self-care, usual activities, pain/discomfort, and anxiety/depression. Each dimension has 3 levels: no problems, some problems, and extreme problems (labelled 1–3). Participants are asked to indicate their health state by ticking the box next to the most appropriate statement in each of the five dimensions. This decision results into a 1-digit number that expresses the level selected for that dimension. The digits for the five dimensions can be combined into a 5-digit number that describes respondent's health state. The EQ-5D-5 L will be analysed to produce an index score between 0 (state of death) and 1 (perfect health).

Physical activity level: Changes in physical activity level will be measured using the Active Australia Survey (AAS).[44] The AAS is a validated self-report tool containing eight core questions to assess participation (hours/mins per week) in moderate and vigorous intensity physical activity and walking for recreation, over the previous week.

For peer review only - http://bmjopen.bmj.com/site/about/guidelines.xhtml

Sleep hygiene behaviours: Changes in sleep hygiene behaviours will be measured using the Pittsburgh Sleep Quality Index (PSQI).[45] The PSQI is a validated self-report survey with 19 self-rated items and 5 items rated by the bed partner or roommate (if applicable). The tool assesses seven components of sleep to provide one global score. Components measured include 1) Subjective sleep quality, 2) Sleep latency, 3) Sleep duration, 4) Habitual sleep, 5) Sleep disturbances, 6) Use of sleeping medication, and 7) Daytime dysfunction. The overall global score of sleep quality will be calculated, and the subcomponents reported.

Health care utilisation: For the purpose of conducting a cost analysis the Consumer Services Receipt Inventory (CSRI)[46] will be completed by participants at each time point. The CSRI is an adaptable tool that ensures the format, language, scope and content is compatible with the research aims, context, participants' likely circumstances, and the quantity and precision of information required.[59] Health care utilisation is captured through self-report and includes information on the number of appointments and type of health care services used in the preceding 3 months.

Cost analysis: A cost-consequence analysis will be conducted including calculating the cost of each intervention (i.e., active, passive and control) and reporting intervention costs alongside mean change outcomes. Intervention costs will be recorded in terms of cost of intervention development, intervention delivery and the operating costs of the RCT. Outcomes to be reported as part of the cost analysis will include mean change in addictive eating symptom scores assessed using the YFAS (i.e. the primary outcome), as well as mean change in the number of health care appointments in the past 3-months assessed using the CSRI, and mean change in Quality Adjusted Life Years (QALYs) assessed using the EQ-5D-5L. This approach was selected to provide a comprehensive and transparent overview of intervention costs, given the lack of cost analysis data in this area of research.[60,61]

For peer review only - http://bmjopen.bmj.com/site/about/guidelines.xhtml

Mediators/Moderators

The following potential mediators and moderators of intervention efficacy will be examined:

Dominant personality trait/s: Participant's will complete the Substance Use Risk Profile Scale (SURPS)[37] at baseline to determine their dominant personality trait/s. The SURPS is a validated self-report 23-item survey that assess four personality traits associated with increased risk for addictive behaviours (Impulsivity proneness, Sensation proneness, Depression proneness, and Anxiety proneness).

Eating behaviours: Eating behaviours that have been shown to have overlap with addictive eating will be measured. This includes eating disorders, binge eating, grazing behaviours and reward driven eating. Eating disorders will be measured using the Eating Disorder Examination Questionnaire 6.0 (EDEQ-6.0)[38] The EDEQ-6.0 is a validated self-report 28-item questionnaire that assesses the occurrence and frequencies of key eating disorder behaviours with cognitive subscales related to eating disorders (restraint, eating concern, shape concern, and weight concern) and behavioural symptoms related to these concerns (e.g. frequency of binge eating, vomiting, use of laxatives or diuretics, and overexercise). Subscale and global scores reflect the severity of eating disorder psychopathology. Binge eating will be measured using the Binge Eating Scale (BES).[39] The BES is a validated self-report 16-item questionnaire to assess the presence of certain binge eating behaviours, over the past 28 days, which may be indicative of an eating disorder. Each item contains 3-4 statements about behaviours, thoughts, and emotional states. Grazing behaviours will be measured using the Short Inventory of Grazing (SIG).[40] The SIG is a validated self-report 2-item measure to assess 1) the presence and frequency of grazing in general, and 2) the presence and frequency of grazing accompanied by a sense of loss of control. Reward driven eating will be measured using the Reward-Based Eating Drive Scale (REDX-5).[41] The REDX-5 is a validated self-report 5-item questionnaire, in 5-point Likert scale format from 1

For peer review only - http://bmjopen.bmj.com/site/about/guidelines.xhtml

(strongly disagree) to 5 (strongly agree), that assesses reward-driven eating (loss of control over eating, lack of satiety, and preoccupation with food).

Participant Activation Level: Participant's underlying knowledge, skills and confidence in managing their addictive eating behaviours and overall health will be measured using the Patient Activation Measure (PAM-13).[62] The PAM-13 is a validated self-report 13-item scale that draws on concepts such as health locus of control, self-efficacy in managing health behaviours and readiness to change health behaviours.[42,63] Higher PAM-13 scores indicate that individuals have higher levels of activation, and understand their role in the self-management process and feel capable of fulfilling that role.[64] Research has demonstrated that a single point change in PAM score is meaningful.[65]

Engagement and use of the program website and Facebook group: Interaction with the website will be objectively tracked throughout the study (baseline to 6 months i.e., timepoints 1 to 3) using Google Analytics (Google LLC). Measures of engagement and usage will include number of website visits, website visit duration, number of page views and links accessed/resources downloaded.

Interaction with the Facebook group will be measured throughout the post-intervention period (3 to 6 months from baseline i.e., timepoints 2 to 3). Measures of engagement and usage will include number of participants to join the Facebook group, and number of views, likes and comments per post.

Study sample characteristics

Sociodemographic data will be collected by online questionnaire at baseline. Participants will provide information on their age, sex, marital status, postal code, years of education, employment status and current living situation. Index of Relative Socio-Economic Disadvantage (IRSD) score,[66] based on the Australian Bureau of Statistics census data and

For peer review only - http://bmjopen.bmj.com/site/about/guidelines.xhtml

reflecting a proxy index of socioeconomic status, will be determined by postal code of residence. Current smoking and substance use will be measured using the Alcohol, Smoking and Substance Involvement Screening Test - Version 3.0.[30] Additionally, previous treatment sought for overeating from health professionals or products used to treat overeating will be collected.

Anthropometric data (self-reported height and weight) will be collected by online questionnaire at baseline. BMI will be calculated using standardised techniques and categorised according to the World Health Organization adult cut-off points.[67]

Sample size

The sample size for the study was calculated based on data from the feasibility study,[16] given the absence of other intervention studies. A clinically meaningful difference in symptoms of addictive eating was selected as a decrease of 2 symptoms, given this would correspond to a change in severity classification on the YFAS 2.0 tool. To detect a mean 2-unit difference (SD = 2.2) in the YFAS symptoms between the active intervention group and the passive intervention group or control group and using a standardised effect size of d=0.91, a sample size of 32 individuals per group (total sample size n=96) is required to detect this change with a power of 0.90 and a type 1 error rate set at 0.025 to account for multiple testing. However, allowing for a 30% dropout rate from the pilot, a sample size of 46 individuals per group (total sample size n=138) would be required. Therefore, a total sample size of 150 individuals, with 50 per group, was chosen to remain conservative.

Statistical analysis plan

Data analysis will be conducted by a researcher blinded to the intervention conditions. Descriptive statistics of sample characteristics will be presented. For the primary YFAS outcome a Linear Mixed Model (LMM) will be based on a model with main effects for group (active intervention, passive intervention, control) and time (treated as categorical at levels

For peer review only

For peer review only - http://bmjopen.bmj.com/site/about/guidelines.xhtml

baseline, 3 and 6 months), and the group-by-time interaction. An unstructured residual covariance structure will be used to allow for correlation between the repeated measurements for a subject. The primary outcome effect will be reported as the difference between means at baseline and 3 months, with a 95% CI for the difference. Mental health condition and BMI will be examined for possible moderating effects on the effect size, and if so adjustment for them will be carried out. Secondary descriptive analysis will be carried out to identify whether specific symptoms were predominantly associated with reductions in YFAS score.

A secondary outcome will be a categorical variable, clinically significant change from baseline to 3 months, where significant requires a reduction of 2 or more symptoms in the YFAS. This will be analysed using logistic regression with group being the only factor. Additional secondary outcomes will include dietary outcomes (average daily energy intake, proportion of total energy intake contributed by core foods and non-core foods intakes, macronutrients intakes, micronutrient intakes; and overall diet quality) and mental health status (depression, anxiety and stress scores). These will also be analysed using LMMs as per the approach above. All available data will be used with no imputation of missing values at 3 and 6 months, however baseline scores will be kept. The participants will be analysed in their allocated randomisation group. Statistical significance will be set at 0.05.

Data management and monitoring

Online survey data will be managed using REDCap electronic data capture tools[68,69] hosted at the University of Newcastle. REDCap (Research Electronic Data Capture) is a secure, web-based software platform designed to support data capture for research studies, providing 1) an intuitive interface for validated data capture; 2) audit trails for tracking data manipulation and export procedures; 3) automated export procedures for seamless data downloads to common statistical packages; and 4) procedures for data integration and interoperability with external sources.

For peer review only - http://bmjopen.bmj.com/site/about/guidelines.xhtml

For peer review only

All data captured from the study website will be encrypted and stored securely on the server. All other data collected will be entered into a password protected central database which is hosted on secure university-based servers, which comply with robust security standards for clinical data and are subject to daily backups and regular offsite backups. Only authorised members of the research team will have access to the database. Research staff handling study data are trained in procedures for handling sensitive information, accurate data entry, surveillance and intervention-specific data storage and data archive. Facilitators of the telehealth sessions are responsible for the electronic storage of study forms on the central database. All completed forms will be checked for completeness and accuracy, first by data collectors and later by a member of the research team responsible for data management. Throughout the study period (at 25% and 50% of required participants) approximately 5% of records will be randomly selected for data quality checks of accuracy and completeness by an independent reviewer.

For the entire study period, any adverse events, of any kind, that might be related to either the trial intervention or trial procedures will be logged in an adverse event log and reported to the Human Research Ethics Committee by the Chief Investigator. To maintain the welfare of participants, with their consent, relevant survey results from the GAD-7[35] and PHQ-9[34] will be sent to the participant's nominated General Practitioner/ health professional if they score in the severe category for either anxiety (GAD-7 scores ≥16) or depression (PHQ-9 scores ≥20) if participants consent to this disclosure.

Study sponsorship and organisation

The sponsor of the trial is the University of Newcastle, and funding was provided by the National Health and Medical Research Council (NHMRC). The trial will be conducted and evaluated independent of the study sponsor and funder. The study is coordinated independently of the study sponsor and funder, by researchers at the University of Newcastle,

For peer review only - http://bmjopen.bmj.com/site/about/guidelines.xhtml

Australia with the study overseen by the trial management committee comprising the chief investigators.

Patient and public involvement

Consumer (i.e., individuals with lived experience of addictive eating who participated in the pilot study) input was received on the pilot version of the intervention (FoodFix process evaluation[16]) that directly guided the enhancement of the TRACE telehealth sessions.

The TRACE program workbook and website for the current study was developed following the pilot study. A sample of consumer representatives (individuals with lived experience of addictive eating and healthcare experts including clinicians and managers), independent of those involved in the pilot study, were involved in the review of the program and program materials.[26]

Consumer representatives were interviewed to:

- Identify what individuals with addictive eating need and want more accurately
- Gather information about what works well and what needs improving, first-hand from consumers who may use them
- Openly consider different or opposing views about aspects of the research project
- Test resources during development and refine resources making sure they will work well in practice
- Detect any unforeseen consequences of a particular decision or direction that has been made regarding the project
- Gain support of consumers to implement changes to the research project

The opinion of consumers has been considered to create a program that:

- Aligns to the needs of the people it is trying to help i.e., individuals with addictive eating

For peer review only - http://bmjopen.bmj.com/site/about/guidelines.xhtml

- Is beneficial in terms of delivering meaningful outcomes for individuals with addictive eating
- Is conducted in a way that is sensitive to peoples' needs

Consumers were not involved in the design of the current study, the selection of outcome measurements, research questions or the recruitment of additional participants. Participants of the current study can request a plain English summary of the study outcomes on its completion.

Ethics and dissemination

The trial will be undertaken in compliance with the Declaration of Helsinki and approval to conduct the study was received from the University of Newcastle Human Research Ethics Committee (H-2021-0100). This trial adheres to the SPIRIT guidelines for randomised trials protocols[28] and the results will be reported in accordance with CONSORT guidelines (TIDieR checklist and guide[27]). Protocol modifications will be registered with the Ethics Committee and trial register. All participants will provide electronic consent to participate prior to completing the eligibility and baseline surveys. Results of the study will be disseminated via peer-reviewed publications and presentations at national and international conferences and will also form part of student dissertations.

Discussion

This study will examine the efficacy of the TRACE personality-based telehealth intervention to reduce addictive eating symptoms, and severity of food addiction, in adults. Examining the efficacy of an intervention that is designed to be scalable is important given that approximately 70% of adults meeting the YFAS criteria for food addiction report ≥ four symptoms, and have significantly lower diet quality, higher intakes of non-core foods and higher weight status.[8-10] Individuals seeking management of addictive eating are also likely to have other mental health comorbidities, such as depression, anxiety, binge eating or other disordered eating

For peer review only

behaviours.[5,7,51] Currently there are few published interventions, run by dietitians and/or other health clinicians, for addictive eating or 'food addiction'[15] demonstrating the clear need for services and trailing of interventions that may be effective at facilitating changes in eating behaviour.

This project will build on the feasibility study utilising the updated program to provide personalised management of addictive eating in adults. We expect that the intervention will provide adults with practical tools (e.g., coping strategies) to improve their eating behaviours and increase awareness of their dietary intakes and potential triggers for overeating, as well as increase help seeking behaviours. Addressing addictive eating may additionally contribute to secondary prevention of other health related issues, such as overweight and obesity, as well as overlapping heavily with validated strategies for the management of some highly prevalent medical conditions, such as cardiovascular disease and type 2 diabetes mellitus.

Strengths of this study include the unique targeting of personality risk factors through tailoring of coping strategies and use of motivational interviewing to increase participant activation as use of these techniques in interventions for other addictive disorders, such as alcohol use, have been shown to be highly effective. While the coping strategies provided by the TRACE program are tailored, it is common for individuals to not uniquely fit into one category for personality and for this reason participants are provided with access via the website to the full set of coping strategies to trial. Telehealth will allow participants to participate from home and reduce the demands on time and travel. Further strengths include the co-design approach taken, with both consumers and a range of health professionals, to avoid siloed research. This collaborative approach aims to provide a program that is relevant and acceptable to end-users, and the randomised controlled design to establish effect. Limitations of the study include the level of experience required of the dietitians administering the telehealth sessions, which may impact the scalability of the intervention. However, dietitians are highly trained professionals in behaviour change and extra care was taken given the uniqueness of the intervention. The

For peer review only - http://bmjopen.bmj.com/site/about/guidelines.xhtml

fidelity outcomes assessed as part of the trial will provide important information regarding future implementation. Additional limitations include the exclusion of individuals with severe mental illnesses or complex health conditions. The current intervention is not designed for complex co-morbidities. It is envisaged that for these individuals a more complex care model is required where the TRACE program could be implemented alongside other approaches or treatments.

The TRACE program is designed to raise awareness, and support behaviour change, of addictive eating. If successful, our study will provide essential evidence regarding the efficacy of behavioural and dietary improvement in the management of addictive eating, thus allowing for the implementation of management strategies for addictive eating into community and clinical healthcare services. Further, if both the active and passive interventions are found to be effective it will provide evidence of different levels of care that could be utilised within these services.

Author Contributions: TLB conceptualised the study, and TLB, JAS, MW, ML, RC, KMP, AVG, PJH, ALB, LH, SJP, LGW, KC and CEC contributed to the study protocol. TLB, JAS, MW, ML, RC, KMP, AVG, PJH, ALB, LH, SJP, and CEC contributed to the intervention development and design, intervention resources and assessment methodology. JAS wrote the initial manuscript draft. TLB, JAS, MW, ML, RC, KMP, AVG, PJH, ALB, LH, SJP, LGW, KC and CEC contributed to the writing of the final manuscript and/or provided critical comments during revisions. All authors approved the final version prior to submission. TLB, JAS, MW, ML and RC will be responsible for recruitment, data collection and intervention delivery.

Competing interests: None declared.

For peer review only - http://bmjopen.bmj.com/site/about/guidelines.xhtml

Funding: This work was supported by the National Health and Medical Research Council (NHMRC) grant number [G1801414].

References

1. Gearhardt AN, Hebebrand J. The concept of “food addiction” helps inform the understanding of overeating and obesity: Debate Consensus. *Am J Clin Nutr*. 2021;113(2):274–6. https://doi.org/10.1093/ajcn/nqaa345

2. Ruddock HK, Christiansen P, Halford JCG, et al. The development and validation of the Addiction-like Eating Behaviour Scale. *Int J Obes (London)*. 2017;41(11):1710-7. https://doi.org/10.1038/ijo.2017.158

3. Burrows T, Verdejo-Garcia A, Carter A, et al. Health Professionals’ and Health Professional Trainees’ Views on Addictive Eating Behaviours: A Cross-Sectional Survey. *Nutrients*. 2020;12(9):E2860. https://doi.org/10.3390/nu12092860

4. Whatnall M, Skinner J, Verdejo-Garcia A, et al. Symptoms of Addictive Eating: What Do Different Health Professions Think? *Behav Sci*. 2021;11(5):60. https://doi.org/10.3390/bs11050060

5. Burrows T, Kay-Lambkin F, Pursey K, et al. Food addiction and associations with mental health symptoms: a systematic review with meta-analysis. *J Hum Nutr Diet*. 2018;31(4):544-72. https://doi.org/10.1111/jhn.12532

6. Pursey K, Stanwell P, Gearhardt A, et al. The prevalence of food addiction as assessed by the Yale Food Addiction Scale: a systematic review. Nutrients. 2014;6(10):4552–90. https://doi.org/10.3390/nu6104552

7. Skinner J, Jebeile H, Burrows T. Food addiction and mental health in adolescents: a systematic review. The Lancet Child and adolescent health. 2021;S2352-4642 (21):00126-7. https://doi.org/10.1016/S2352-4642(21)00126-7

For peer review only

8. Pursey KM, Skinner J, Leary M, et al. The Relationship between Addictive Eating and Dietary Intake: A Systematic Review. Nutrients. 2021(1):164. https://doi.org/10.3390/nu14010164

9. Pursey KM, Collins CE, Stanwell P, et al. Foods and dietary profiles associated with 'food addiction' in young adults. Addictive Behaviors Reports 2015;2:41–8. http://dx.doi.org/10.1016/j.abrep.2015.05.007

10. Burrows T, Hides L, Brown R, et al. Differences in Dietary Preferences, Personality and Mental Health in Australian Adults with and without Food Addiction. Nutrients. 2017;9 (3):E285. https://doi.org/10.3390/nu9030285

11. Burrows T, Skinner J, McKenna R, et al. Food Addiction, Binge Eating Disorder, and Obesity: Is There a Relationship? *Behav Sci*. 2017;7(3):pii: E54. https://doi.org/10.3390/bs7030054

12. Food Addicts Anonymous [Homepage], USA, accessed 24 March 2022. Available from: https://faacanhelp.org/

13. Overeaters Anonymous [Homepage], USA, accessed 24 March 2022. Available from: https://oa.org/

14. McKenna RA, Rollo ME, Skinner JA, et al. Food Addiction Support: Website Content Analysis. JMIR Cardio. 2018;2(1):e10. https://doi.org/10.2196/cardio.8718

15. Leary M, Pursey KM, Verdejo-Garcia A, et al. Current Intervention Treatments for Food Addiction: A Systematic Review. *Behav Sci*. 2021;11(6):80. https://doi.org/10.3390/bs11060080

16. Burrows T, Collins R, Rollo M, et al. The feasibility of a personality targeted intervention for addictive overeating: FoodFix. *Appetite*. 2021;156(104974). https://doi.org/10.1016/j.appet.2020.104974

17. Gearhardt AN, Corbin WR, Brownell KD. Preliminary validation of the Yale Food Addiction Scale. *Appetite*. 2009;52(2):430-6. https://doi.org/10.1016/j.appet.2008.12.003

For peer review only - http://bmjopen.bmj.com/site/about/guidelines.xhtml

18. Gearhardt AN, Corbin WR, Brownell KD. Development of the Yale Food Addiction Scale Version 2.0. *Psychol Addict Behav*. 2016;30(1):113-21. https://doi.org/10.1037/adb0000136

19. Carbone EA, Caroleo M, Rania M, et al. An open-label trial on the efficacy and tolerability of naltrexone/bupropion SR for treating altered eating behaviours and weight loss in binge eating disorder. *Eat Weight Disord*. 2021;26(3):779–88. https://doi.org/10.1007/s40519-020-00910-x

20. Epstein DH, Kennedy AP, Furnari M, et al. Effect of the CRF1-receptor antagonist pexacerfont on stress-induced eating and food craving. *Psychopharmacology (Berl)*. 2016;233(23-24):3921–32. https://doi.org/10.1007/s00213-016-4424-5

21. Sevinçer GM, Konuk N, Bozkurt S, et al. Food addiction and the outcome of bariatric surgery at 1-year: Prospective observational study. *Psychiatry Res*. 2016;244 159–64. https://doi.org/10.1016/j.psychres.2016.07.022

22. Murray SM, Tweardy S, Geliebter A, et al. A Longitudinal Preliminary Study of Addiction-Like Responses to Food and Alcohol Consumption Among Individuals Undergoing Weight Loss Surgery. *Obes Surg*. 2019;29(8):2700–3. https://doi.org/10.1007/s11695-019-03915-3

23. Chao AM, Wadden TA, Tronieri JS, et al. Effects of addictive-like eating behaviors on weight loss with behavioral obesity treatment. *J Behav Med*. 2019;42(2):246–55. https://doi.org/10.1007/s10865-018-9958-z

24. Hides L, Kavanagh DJ, Daglish M, et al. The Quik Fix study: a randomised controlled trial of brief interventions for young people with alcohol-related injuries and illnesses accessing emergency department and crisis support care. *BMC Emerg Med*. 2014;14(19). https://doi.org/10.1186/1471-227X-14-19

25. Hides L, Wilson H, Quinn C, et al. QuikFix: enhanced motivational interviewing interventions for youth substance use. *Adv Dual Diagn*. 2016;9(2/3):53-65. https://doi.org/10.1108/ADD-03-2016-0008

For peer review only - http://bmjopen.bmj.com/site/about/guidelines.xhtml

26. Leary M, Pursey KM, Verdejo-Garcia A, et al. Designing an online intervention for adults with addictive eating: A qualitative Integrated Knowledge Translation approach. *BMJ Open*. 2022;In press.

27. Hoffmann T, Glasziou P, Boutron I, et al. Better reporting of interventions: template for intervention description and replication (TIDieR) checklist and guide. *BMJ*. 2014;348:g1687. https://doi.org/10.1136/bmj.g1687

28. Chan A-W, Tetzlaff JM, Altman DG, et al. SPIRIT 2013 Statement: Defining standard protocol items for clinical trials. Annals of Internal Medicine. 2013;158(3):200-7. https://doi.org/10.7326/0003-4819-158-3-201302050-00583

29. Schulz KF, Altman DG, Moher D, et al. CONSORT 2010 statement: updated guidelines for reporting parallel group randomised trials. *BMJ*. 2010;340(c332). https://doi.org/10.1136/bmj.c332

30. WHO ASSIST Working Group. The Alcohol, Smoking and Substance Involvement Screening Test (ASSIST): development, reliability and feasibility. *Addiction*. 2002;97(9):1183-94. https://doi.org/10.1046/j.1360-0443.2002.00185.x

31. Prnjak K, Mitchison D, Griffiths S, et al. Further development of the 12-item EDE-QS: identifying a cut-off for screening purposes. *BMC Psychiatry*. 2020;20(1):146. https://doi.org/10.1186/s12888-020-02565-5

32. Collins CE, Boggess MM, Watson JF, et al. Reproducibility and comparative validity of a food frequency questionnaire for Australian adults. *Clin Nutr*. 2014;33(5):906-14. https://doi.org/10.1016/j.clnu.2013.09.015

33. Ashton L, Williams R, Wood L, et al. Comparison of Australian Recommended Food Score (ARFS) and Plasma Carotenoid Concentrations: A Validation Study in Adults. *Nutrients*. 2017;9(8):888. http://doi.org/10.3390/nu9080888

34. Martin A, Rief W, Klaiberg A, et al. Validity of the Brief Patient Health Questionnaire Mood Scale (PHQ-9) in the general population. *Gen Hosp Psychiatry*. 2006;28(1):71-7. https://doi.org/10.1016/j.genhosppsych.2005.07.003

For peer review only

For peer review only - http://bmjopen.bmj.com/site/about/guidelines.xhtml

35. Spitzer RL, Kroenke K, Williams JBW, et al. A Brief Measure for Assessing Generalized Anxiety Disorder (The GAD-7). *Arch Intern Med*. 2006;166(10):1092–7 https://doi.org/10.1001/archinte.166.10.1092

36. Ingram PB 4th, Clarke E, Lichtenberg JW. Confirmatory Factor Analysis of the Perceived Stress Scale-4 in a Community Sample. *Stress Health*. 2016;32(2):173–6. https://doi.org/10.1002/smi.2592

37. Woicik PA, Stewart SH, Pihl RO, et al. The Substance Use Risk Profile Scale: a scale measuring traits linked to reinforcement-specific substance use profiles. *Addict Behav*. 2009;34(12):1042-55. https://doi.org/10.1016/j.addbeh.2009.07.001

38. Fairburn C, Cooper Z, O'Connor M. Eating disorders examination (16.0D). In: Fairburn C, ed. Cognitive behavior therapy and eating disorders. New York: Guilford Press 2008.

39. Gormally J, Black S, Daston S, et al. The assessment of binge eating severity among obese persons. *Addict Behav*. 1982;7(1):47-55. https://doi.org/10.1016/0306-4603(82)90024-7

40. Heriseanu AI, Hay P, Touyz S. The short inventory of grazing (SIG): development and validation of a new brief measure of a common eating behaviour with a compulsive dimension. *J Eat Disord*. 2019;7(4). https://doi.org/10.1186/s40337-019-0234-6

41. Vainik U, Han C, Epel ES, et al. Rapidly assessing reward-related eating: The RED-X5. *Obesity (Silver Spring)*. 2019;27(2):325–31. https://doi.org/10.1002/oby.22374

42. Hibbard JH, Stockard J, Mahoney ER, et al. Development of the Patient Activation Measure (PAM): conceptualizing and measuring activation in patients and consumers. *Health Serv Res*. 2004;39(4 Pt 1):1005–26. https://doi.org/10.1111/j.1475-6773.2004.00269.x

43. Rabin R, de Charro F. EQ-5D: a measure of health status from the EuroQol Group. *Ann Med*. 2001;33(5):337-43. https://doi.org/10.3109/07853890109002087

44. Australian Institute of Health and Welfare (AIHW). The Active Australia Survey: a guide and manual for implementation, analysis and reporting. Canberra, Australia: 2003.

For peer review only

For peer review only - http://bmjopen.bmj.com/site/about/guidelines.xhtml

Accessed 22 March 2022. Available from: https://www.aihw.gov.au/reports/physical-activity/active-australia-survey/summary

45. Buysse DJ, Reynolds CF 3rd, Monk TH, et al. The Pittsburgh Sleep Quality Index: a new instrument for psychiatric practice and research. *Psychiatry Res*. 1989;28(2):193–213. https://doi.org/10.1016/0165-1781(89)90047-4

46. Beecham J, Knapp M. Costing psychiatric interventions. In: Thornicroft G, ed. Measuring Mental Health Needs (2nd edition). London: Gaskell 2001: 200-24.

47. Australian Government Department of Health. Body mass index (BMI) and waist measurement. Canberra, Australia: 2021. Accessed 22 March 2022. Available from: https://www.health.gov.au/health-topics/overweight-and-obesity/bmi-and-waist

48. Centers for Disease Control and Prevention (CDC). Healthy Weight, Nutrition, and Physical Activity: Assessing Your Weight. USA: 2021. Accessed 22 March 2022. Available from: https://www.cdc.gov/healthyweight/assessing/index.html

49. Mond J, Hay P, Rodgers B, et al. Use of extreme weight control behaviors with and without binge eating in a community sample: implications for the classification of bulimic-type eating disorders. *Int J Eat Disord*. 2006;39(4):294-302. https://doi.org/10.1002/eat.20265

50. Mond JJ, Hay PJ, Rodgers B, et al. Correlates of the use of purging and non-purging methods of weight control in a community sample of women. *Aust N Z J Psychiatry*. 2006;40(2):136-42. https://doi.org/10.1080/j.1440-1614.2006.01760.x

51. Pursey KM, Collins R, Skinner J, et al. Characteristics of individuals seeking addictive eating treatment. *Eat Weight Disord*. 2021. https://doi.org/10.1007/s40519-021-01147-y

52. Collins R, Haracz K, Leary M, et al. No control and overwhelming cravings: Australian adults' perspectives on the experience of food addiction. *Appetite*. 2021;59(105054). https://doi.org/10.1016/j.appet.2020.105054

53. American Psychiatric Association. Diagnostic and statistical manual of mental disorders. 5th ed. Arlington: VA American Psychiatric Publishing; 2013.

For peer review only

For peer review only - http://bmjopen.bmj.com/site/about/guidelines.xhtml

54. Pedram P, Wadden D, Amini P, et al. Food addiction: its prevalence and significant association with obesity in the general population. *PLOS One*. 2013;8(9):e74832. https://doi.org/10.1371/journal.pone.0074832

55. Lemeshow AR, Gearhardt AN, Genkinger JM, et al. Assessing the psychometric properties of two food addiction scales. *Eat Behav*. 2016;23:110–4. https://doi.org/10.1016/j.eatbeh.2016.08.005

56. National Health and Medical Research Council (NHMRC). Australian Guide to Healthy Eating. Canberra, Australia: 2017. Accessed 22 April 2022. Available from: https://www.eatforhealth.gov.au/guidelines/australian-guide-healthy-eating

57. Food Standards Australia New Zealand (FSANZ). AUSNUT 2011–13–Australian Food Composition Database. Kingston, ACT, Australia: 2014. Accessed 22 March 2022. Available from: http://foodstandards.gov.au/

58. National Health and Medical Research Council. Australian Dietary Guidelines. Canberra, Australia: 2013. Accessed 22 March 2022. Available from: www.nhmrc.gov.au/guidelines-publications/n55

59. Personal Social Services Research Unit (PSSRU). Canterbury, Kent, UK: University of Kent; 2022. Accessed 14 April 2022. Available from: https://www.pssru.ac.uk/csri/client-service-receipt-inventory/

60. Mauskopf JA, Paul JE, Grant DM, et al. The role of cost-consequence analysis in healthcare decision-making. Pharmacoeconomics. 1998;13(3):277-88. https://doi.org/10.2165/00019053-199813030-00002

61. Hunter R, James Shearer J. Cost-consequences analysis - an underused method of economic evaluation. United Kingdom: National Institute for Health Research. Accessed 14 April 2022. Available from: https://www.rds-london.nihr.ac.uk/wpcms/wp-content/uploads/2018/09/Cost-consequences-analysis-an-underused-method.pdf.

62. Hibbard JH, Mahoney ER, Stockard J, et al. Development and testing of a short form of the patient activation measure. *Health Serv Res*. 2005;40(6 Pt 1):1918-30. https://doi.org/10.1111/j.1475-6773.2005.00438.x

For peer review only - http://bmjopen.bmj.com/site/about/guidelines.xhtml

63. Greene J, Hibbard JH. Why does patient activation matter? An examination of the relationships between patient activation and health-related outcomes. *J Gen Intern Med*. 2012;27(5):520–6. https://doi.org/10.1007/s11606-011-1931-2

64. Insigina Health. Patient activation measure (PAM) 13 TM Licence Materials copyright. LLC: Insigna Health; 2011.

65. Remmers C, Hibbard J, Mosen DM, et al. Is patient activation associated with future health outcomes and healthcare utilization among patients with diabetes? *J Ambul Care Manage*. 2009;32(4):320-7. https://doi.org/10.1097/JAC.0b013e3181ba6e77

66. Australian Bureau of Statistics (ABS). 2033.0.55.001 - Socio-Economic Indexes for Areas (SEIFA). Canberra, Australia: ABS; 2016. Accessed 14 September 2021. Available from: www.abs.gov.au

67. World Health Organization (WHO). Body mass index – BMI. WHO Regional Office for Europe; c2018. Accessed 14 September 2021. Available from: http://www.euro.who.int/en/health-topics/disease-prevention/nutrition/a-healthy-lifestyle/body-mass-index-bmi

68. Harris PA, Taylor R, Minor BL, et al. REDCap Consortium. The REDCap consortium: Building an international community of software platform partners. *J Biomed Inform*. 2019;95(103208). https://doi.org/10.1016/j.jbi.2019.103208

69. Harris PA, Taylor R, Thielke R, et al. Research electronic data capture (REDCap) – A metadata-driven methodology and workflow process for providing translational research informatics support. *J Biomed Inform*. 2009;42(2):377-81. https://doi.org/10.1016/j.jbi.2008.08.010

References for Table 1

1. WHO ASSIST Working Group. The Alcohol, Smoking and Substance Involvement Screening Test (ASSIST): development, reliability and feasibility. *Addiction*. 2002;97(9):1183-94. https://doi.org/10.1046/j.1360-0443.2002.00185.x

For peer review only

For peer review only - http://bmjopen.bmj.com/site/about/guidelines.xhtml

2. Prnjak K, Mitchison D, Griffiths S, et al. Further development of the 12-item EDE-QS: identifying a cut-off for screening purposes. *BMC Psychiatry*. 2020;20(1):146. https://doi.org/10.1186/s12888-020-02565-5

3. Gearhardt AN, Corbin WR, Brownell KD. Development of the Yale Food Addiction Scale Version 2.0. *Psychol Addict Behav*. 2016;30(1):113-21. https://doi.org/10.1037/adb0000136

4. Collins CE, Boggess MM, Watson JF, et al. Reproducibility and comparative validity of a food frequency questionnaire for Australian adults. *Clin Nutr*. 2014;33(5):906-14. https://doi.org/10.1016/j.clnu.2013.09.015

5. Ashton L, Williams R, Wood L, et al. Comparison of Australian Recommended Food Score (ARFS) and Plasma Carotenoid Concentrations: A Validation Study in Adults. *Nutrients*. 2017;9(8):888. http://doi.org/10.3390/nu9080888

6. Martin A, Rief W, Klaiberg A, et al. Validity of the Brief Patient Health Questionnaire Mood Scale (PHQ-9) in the general population. *Gen Hosp Psychiatry*. 2006;28(1):71-7. https://doi.org/10.1016/j.genhosppsych.2005.07.003

7. Spitzer RL, Kroenke K, Williams JBW, et al. A Brief Measure for Assessing Generalized Anxiety Disorder (The GAD-7). *Arch Intern Med*. 2006;166(10):1092–7 https://doi.org/10.1001/archinte.166.10.1092

8. Ingram PB 4th, Clarke E, Lichtenberg JW. Confirmatory Factor Analysis of the Perceived Stress Scale-4 in a Community Sample. *Stress Health*. 2016;32(2):173–6. https://doi.org/10.1002/smi.2592

9. Woicik PA, Stewart SH, Pihl RO, et al. The Substance Use Risk Profile Scale: a scale measuring traits linked to reinforcement-specific substance use profiles. *Addict Behav*. 2009;34(12):1042-55. https://doi.org/10.1016/j.addbeh.2009.07.001

10. Fairburn C, Cooper Z, O'Connor M. Eating disorders examination (16.0D). In: Fairburn C, ed. Cognitive behavior therapy and eating disorders. New York: Guilford Press 2008.

For peer review only

For peer review only - http://bmjopen.bmj.com/site/about/guidelines.xhtml

11. Gormally J, Black S, Daston S, et al. The assessment of binge eating severity among obese persons. *Addict Behav*. 1982;7(1):47-55. https://doi.org/10.1016/0306-4603(82)90024-7

12. Heriseanu AI, Hay P, Touyz S. The short inventory of grazing (SIG): development and validation of a new brief measure of a common eating behaviour with a compulsive dimension. *J Eat Disord*. 2019;7(4). https://doi.org/10.1186/s40337-019-0234-6

13. Vainik U, Han C, Epel ES, et al. Rapidly assessing reward-related eating: The RED-X5. *Obesity (Silver Spring)*. 2019;27(2):325–31. https://doi.org/10.1002/oby.22374

14. Hibbard JH, Stockard J, Mahoney ER, et al. Development of the Patient Activation Measure (PAM): conceptualizing and measuring activation in patients and consumers. *Health Serv Res*. 2004;39(4 Pt 1):1005–26. https://doi.org/10.1111/j.1475-6773.2004.00269.x

15. Rabin R, de Charro F. EQ-5D: a measure of health status from the EuroQol Group. *Ann Med*. 2001;33(5):337-43. https://doi.org/10.3109/07853890109002087

16. Australian Institute of Health and Welfare (AIHW). The Active Australia Survey: a guide and manual for implementation, analysis and reporting. Canberra, Australia: 2003. Accessed 22 March 2022. Available from: https://www.aihw.gov.au/reports/physical-activity/active-australia-survey/summary

17. Buysse DJ, Reynolds CF 3rd, Monk TH, et al. The Pittsburgh Sleep Quality Index: a new instrument for psychiatric practice and research. *Psychiatry Res*. 1989;28(2):193–213. https://doi.org/10.1016/0165-1781(89)90047-4

18. Beecham J, Knapp M. Costing psychiatric interventions. In: Thornicroft G, ed. Measuring Mental Health Needs (2nd edition). London: Gaskell 2001: 200-24.

For peer review only - http://bmjopen.bmj.com/site/about/guidelines.xhtml

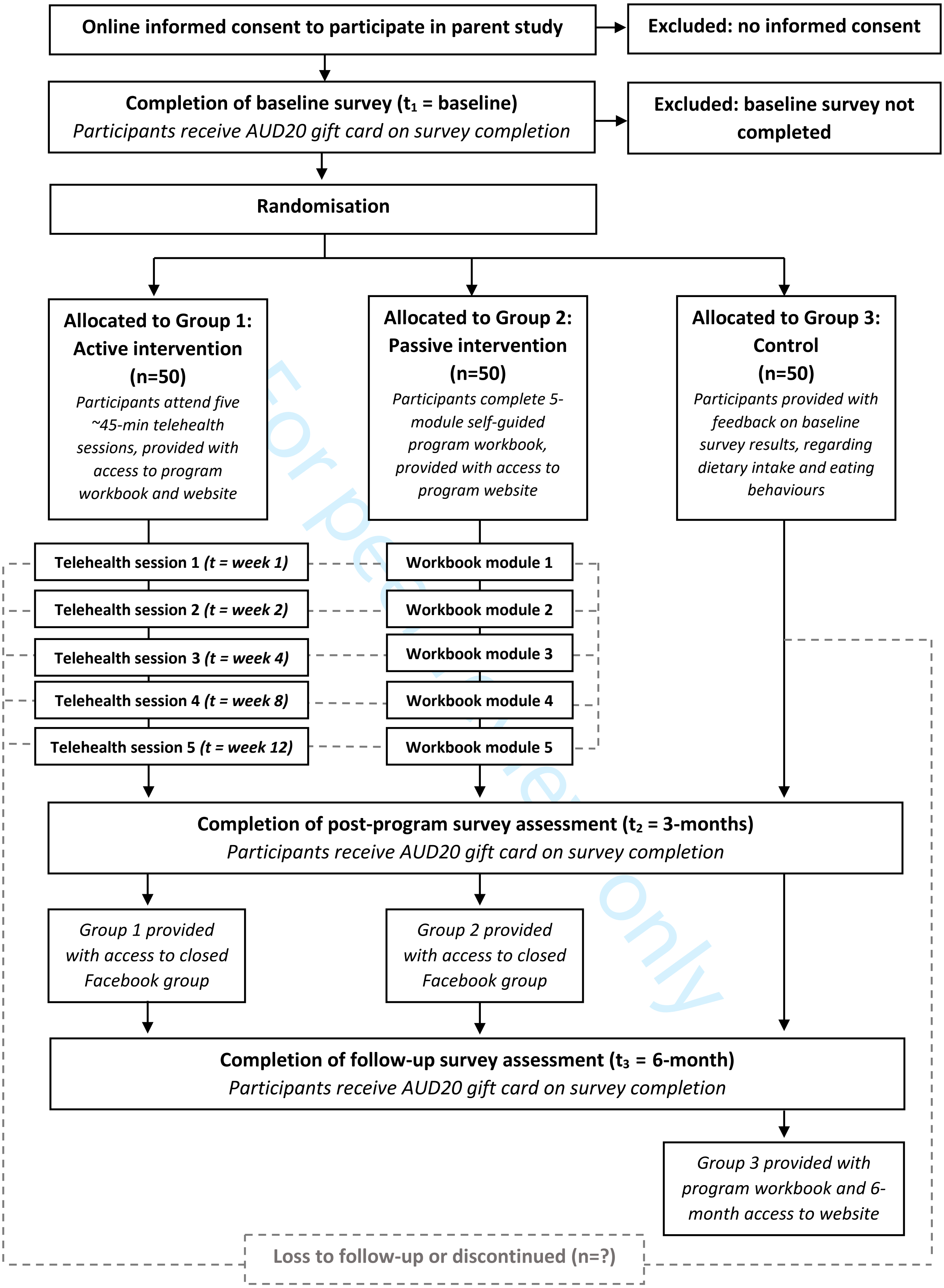

For peer review only

SPIRIT
STANDARD PROTOCOL ITEMS: RECOMMENDATIONS FOR INTERVENTIONAL TRIALS

SPIRIT 2013 Checklist: Recommended items to address in a clinical trial protocol and related documents*

Section/item	Item No	Description	Addressed on page number
**Administrative information**			
Title	1	Descriptive title identifying the study design, population, interventions, and, if applicable, trial acronym	2
Trial registration	2a	Trial identifier and registry name. If not yet registered, name of intended registry	3, 6
	2b	All items from the World Health Organization Trial Registration Data Set	NA
Protocol version	3	Date and version identifier	-
Funding	4	Sources and types of financial, material, and other support	27
Roles and responsibilities	5a	Names, affiliations, and roles of protocol contributors	1, 31
	5b	Name and contact information for the trial sponsor	1
	5c	Role of study sponsor and funders, if any, in study design; collection, management, analysis, and interpretation of data; writing of the report; and the decision to submit the report for publication, including whether they will have ultimate authority over any of these activities	7
	5d	Composition, roles, and responsibilities of the coordinating centre, steering committee, endpoint adjudication committee, data management team, and other individuals or groups overseeing the trial, if applicable (see Item 21a for data monitoring committee)	NA

For peer review only

Introduction

Background and rationale	6a	Description of research question and justification for undertaking the trial, including summary of relevant studies (published and unpublished) examining benefits and harms for each intervention	4, 5
	6b	Explanation for choice of comparators	6
Objectives	7	Specific objectives or hypotheses	5
Trial design	8	Description of trial design including type of trial (eg, parallel group, crossover, factorial, single group), allocation ratio, and framework (eg, superiority, equivalence, noninferiority, exploratory)	6, 7

Methods: Participants, interventions, and outcomes

Study setting	9	Description of study settings (eg, community clinic, academic hospital) and list of countries where data will be collected. Reference to where list of study sites can be obtained	7
Eligibility criteria	10	Inclusion and exclusion criteria for participants. If applicable, eligibility criteria for study centres and individuals who will perform the interventions (eg, surgeons, psychotherapists)	9
Interventions	11a	Interventions for each group with sufficient detail to allow replication, including how and when they will be administered	10 - 18
	11b	Criteria for discontinuing or modifying allocated interventions for a given trial participant (eg, drug dose change in response to harms, participant request, or improving/worsening disease)	7
	11c	Strategies to improve adherence to intervention protocols, and any procedures for monitoring adherence (eg, drug tablet return, laboratory tests)	17, 18
	11d	Relevant concomitant care and interventions that are permitted or prohibited during the trial	NA
Outcomes	12	Primary, secondary, and other outcomes, including the specific measurement variable (eg, systolic blood pressure), analysis metric (eg, change from baseline, final value, time to event), method of aggregation (eg, median, proportion), and time point for each outcome. Explanation of the clinical relevance of chosen efficacy and harm outcomes is strongly recommended	19 - 22
Participant timeline	13	Time schedule of enrolment, interventions (including any run-ins and washouts), assessments, and visits for participants. A schematic diagram is highly recommended (see Figure)	8 (Table 1)

For peer review only - http://bmjopen.bmj.com/site/about/guidelines.xhtml

Sample size	14	Estimated number of participants needed to achieve study objectives and how it was determined, including clinical and statistical assumptions supporting any sample size calculations	25
Recruitment	15	Strategies for achieving adequate participant enrolment to reach target sample size	7
**Methods: Assignment of interventions (for controlled trials)**			
Allocation:			
Sequence generation	16a	Method of generating the allocation sequence (eg, computer-generated random numbers), and list of any factors for stratification. To reduce predictability of a random sequence, details of any planned restriction (eg, blocking) should be provided in a separate document that is unavailable to those who enrol participants or assign interventions	12
Allocation concealment mechanism	16b	Mechanism of implementing the allocation sequence (eg, central telephone; sequentially numbered, opaque, sealed envelopes), describing any steps to conceal the sequence until interventions are assigned	12
Implementation	16c	Who will generate the allocation sequence, who will enrol participants, and who will assign participants to interventions	12
Blinding (masking)	17a	Who will be blinded after assignment to interventions (eg, trial participants, care providers, outcome assessors, data analysts), and how	12, 13
	17b	If blinded, circumstances under which unblinding is permissible, and procedure for revealing a participant's allocated intervention during the trial	NA
**Methods: Data collection, management, and analysis**			
Data collection methods	18a	Plans for assessment and collection of outcome, baseline, and other trial data, including any related processes to promote data quality (eg, duplicate measurements, training of assessors) and a description of study instruments (eg, questionnaires, laboratory tests) along with their reliability and validity, if known. Reference to where data collection forms can be found, if not in the protocol	17, 19-24, 27
	18b	Plans to promote participant retention and complete follow-up, including list of any outcome data to be collected for participants who discontinue or deviate from intervention protocols	12

Data management	19	Plans for data entry, coding, security, and storage, including any related processes to promote data quality (eg, double data entry; range checks for data values). Reference to where details of data management procedures can be found, if not in the protocol	26, 27
Statistical methods	20a	Statistical methods for analysing primary and secondary outcomes. Reference to where other details of the statistical analysis plan can be found, if not in the protocol	25, 26
	20b	Methods for any additional analyses (eg, subgroup and adjusted analyses)	25, 26
	20c	Definition of analysis population relating to protocol non-adherence (eg, as randomised analysis), and any statistical methods to handle missing data (eg, multiple imputation)	26
**Methods: Monitoring**			
Data monitoring	21a	Composition of data monitoring committee (DMC); summary of its role and reporting structure; statement of whether it is independent from the sponsor and competing interests; and reference to where further details about its charter can be found, if not in the protocol. Alternatively, an explanation of why a DMC is not needed	NA
	21b	Description of any interim analyses and stopping guidelines, including who will have access to these interim results and make the final decision to terminate the trial	NA
Harms	22	Plans for collecting, assessing, reporting, and managing solicited and spontaneously reported adverse events and other unintended effects of trial interventions or trial conduct	27
Auditing	23	Frequency and procedures for auditing trial conduct, if any, and whether the process will be independent from investigators and the sponsor	27
**Ethics and dissemination**			
Research ethics approval	24	Plans for seeking research ethics committee/institutional review board (REC/IRB) approval	6, 29
Protocol amendments	25	Plans for communicating important protocol modifications (eg, changes to eligibility criteria, outcomes, analyses) to relevant parties (eg, investigators, REC/IRBs, trial participants, trial registries, journals, regulators)	NA

Consent or assent	26a	Who will obtain informed consent or assent from potential trial participants or authorised surrogates, and how (see Item 32)	7, 10, 11
	26b	Additional consent provisions for collection and use of participant data and biological specimens in ancillary studies, if applicable	NA
Confidentiality	27	How personal information about potential and enrolled participants will be collected, shared, and maintained in order to protect confidentiality before, during, and after the trial	26, 27
Declaration of interests	28	Financial and other competing interests for principal investigators for the overall trial and each study site	31
Access to data	29	Statement of who will have access to the final trial dataset, and disclosure of contractual agreements that limit such access for investigators	26, 27
Ancillary and post-trial care	30	Provisions, if any, for ancillary and post-trial care, and for compensation to those who suffer harm from trial participation	NA
Dissemination policy	31a	Plans for investigators and sponsor to communicate trial results to participants, healthcare professionals, the public, and other relevant groups (eg, via publication, reporting in results databases, or other data sharing arrangements), including any publication restrictions	29
	31b	Authorship eligibility guidelines and any intended use of professional writers	NA
	31c	Plans, if any, for granting public access to the full protocol, participant-level dataset, and statistical code	NA
**Appendices**			
Informed consent materials	32	Model consent form and other related documentation given to participants and authorised surrogates	NA
Biological specimens	33	Plans for collection, laboratory evaluation, and storage of biological specimens for genetic or molecular analysis in the current trial and for future use in ancillary studies, if applicable	NA

*It is strongly recommended that this checklist be read in conjunction with the SPIRIT 2013 Explanation & Elaboration for important clarification on the items. Amendments to the protocol should be tracked and dated. The SPIRIT checklist is copyrighted by the SPIRIT Group under the Creative Commons "Attribution-NonCommercial-NoDerivs 3.0 Unported" license.

For peer review only

BMJ Open

Examining the efficacy of a telehealth intervention targeting addictive eating in Australian adults (the TRACE program): a randomised controlled trial protocol

Journal:	*BMJ Open*
Manuscript ID	bmjopen-2022-064151.R1
Article Type:	Protocol
Date Submitted by the Author:	09-Mar-2023
Complete List of Authors:	Skinner, Janelle; The University of Newcastle, School of Health<br>Whatnall, Megan; The University of Newcastle, School of Health<br>Leary, Mark; The University of Newcastle,<br>Collins, Rebecca; The University of Newcastle, School of Health<br>Pursey, Kirrilly; The University of Newcastle, School of Health<br>Verdejo-García, Antonio; Monash University<br>Hay, Phillipa ; Western Sydney University<br>Baker, Amanda; The University of Newcastle,<br>Hides, Leanne; University of Queensland,<br>Paxton, Susan; La Trobe University<br>Wood, Lisa; The University of Newcastle, Respiratory Medicine<br>Colyvas, Kim; The University of Newcastle, School of Mathematical and Physical Sciences<br>Collins, Clare; The University of Newcastle<br>Burrows, Tracy; The University of Newcastle,
**Primary Subject Heading**:	Addiction
Secondary Subject Heading:	Mental health
Keywords:	NUTRITION & DIETETICS, PUBLIC HEALTH, Eating disorders < PSYCHIATRY

SCHOLARONE™
Manuscripts

Article type: Protocol

Title: Examining the efficacy of a telehealth intervention targeting addictive eating in Australian adults (the TRACE program): a randomised controlled trial protocol

Authors: Janelle A Skinner[1,2], Megan Whatnall[1,2], Mark Leary[1,2], Rebecca A Collins[1,2], Kirrilly M Pursey[1,2], Antonio Verdejo-Garcia[3], Phillipa J Hay[4], Amanda L Baker[2,5], Leanne Hides[6], Susan J Paxton[7], Lisa G Wood[2,8], Kim Colyvas[1], Clare E Collins[1,2], Tracy L Burrows[1,2*]

Institutions:

[1]School of Health Sciences, College of Health, Medicine and Wellbeing, University of Newcastle, Callaghan NSW 2308, Australia

[2]Hunter Medical Research Institute, University of Newcastle, New Lambton Heights, NSW, 2305, Australia

[3]School of Psychological Sciences and Turner Institute for Brain and Mental Health, Monash University, Clayton, VIC 3800, Australia

[4]Translational Health Research Institute, Western Sydney University, Campbelltown, NSW, 2751, Australia

[5]School of Medicine and Public Health, College of Health, Medicine and Wellbeing, University of Newcastle, Callaghan, NSW 2308, Australia

[6]School of Psychology, University of Queensland, St Lucia, QLD, 4072, Australia

[7]Department of Psychology and Counselling, Latrobe University, Melbourne, VIC, 3086

[8]School of Biomedical Sciences and Pharmacy, College of Health, Medicine and Wellbeing, University of Newcastle, Callaghan NSW 2308, Australia

***Corresponding Author:** Professor Tracy Burrows. Mailing address: School of Health Sciences, University Drive, University of Newcastle, Callaghan, NSW, Australia, 2308. Ph: +610249215514. Email: tracy.burrows@newcastle.edu.au

Word count: 7910

Key words: addictive eating, food addiction, Yale Food Addiction Scale

For peer review only

For peer review only - http://bmjopen.bmj.com/site/about/guidelines.xhtml

ARTICLE SUMMARY

Title: Examining the efficacy of a telehealth intervention targeting addictive eating in Australian adults (the TRACE program): a randomised controlled trial protocol

Abstract

Introduction *A*pproximately 15-20% of the adult population endorse symptoms of addictive eating and there are currently limited options for management. Motivational interviewing-based interventions, containing personalised coping skills training for addictive disorders, have been found to be effective for behaviour change. This project builds upon foundations of a feasibility study, and co-design process involving consumers. The primary aim of this three-arm randomised controlled trial is to examine the efficacy of a telehealth intervention targeting addictive eating symptoms in Australian adults compared to passive intervention and control (no intervention) groups. **Methods and analysis** Participants, aged 18-85 years, endorsing ≥3 symptoms on the Yale Food Addiction Scale 2.0, with BMI >18.5kg/m^2 will be recruited. Addictive eating symptoms are assessed at baseline (pre-intervention), 3 months (post-intervention) and 6 months. Other outcomes will include dietary intake and quality, depression, anxiety, stress, quality of life, physical activity, and sleep hygiene. Using a multicomponent clinician-led approach, the active intervention consists of five telehealth sessions (15-45min each) delivered by a dietitian over 3 months. The intervention uses personalised feedback, skill-building exercises, reflective activities, and goal setting. Participants are provided with a workbook and web site access. The passive intervention group receive the intervention via a self-guided approach with access to the workbook and website (no telehealth). The control group receive personalised written dietary feedback at baseline and participants advised to follow their usual dietary pattern for six months. The control group will be offered the passive intervention after 6-months. The primary endpoint is YFAS symptom scores at 3 months. A cost consequence analysis will determine intervention costs alongside mean change outcomes. **Ethics and dissemination** The Human Research Ethics Committee of The

For peer review only

For peer review only - http://bmjopen.bmj.com/site/about/guidelines.xhtml

University of Newcastle Australia provided approval: H-2021-0100. Findings will be disseminated via publication in peer-reviewed journals, conference presentations, community presentations and student theses. Trial registration: Australia New Zealand Clinical Trial Registry (ANZCTR) ACTRN12621001079831.

Strengths and Limitations of this study

- Targeting addictive risk factors through personalised tailoring of coping strategies and use of motivational interviewing for management of symptoms of addictive eating
- Co-design approach taken, with both consumers and multidisciplinary health professionals, to inform program development
- Detailed assessment of eating behaviours, mental health and lifestyle factors with personalised feedback provided to participants during the telehealth intervention
- Fidelity outcomes will be assessed, and cost consequence analysis conducted regarding implementation
- Limitations include participants being excluded with severe mental illnesses or complex health conditions

For peer review only - http://bmjopen.bmj.com/site/about/guidelines.xhtml

INTRODUCTION

Research in addictive eating has increased rapidly in recent years. Addictive eating, theorised as being on the severe end of a spectrum of overeating,[1] is a phenotype of eating behaviour marked by the chronic excessive and dysregulated consumption of food.[2, 3] Addictive eating or 'food addiction' is accepted by consumers[4] and health practitioners,[5, 6] though the construct remains contentious in the scientific community with ongoing debate regarding terms and definitions.[7] Specifically, whether the construct should be described as a behavioural disorder or as a substance-related addiction, whereby certain foods or components in foods are capable of activating an addictive-like process in susceptible individuals.[8] Addictive eating, not categorised as a distinct disorder in the Diagnostic and Statistical Manual of Mental Disorders (DSM)[9] or the International Classification of Disease[10] systems, is most commonly assessed using the Yale Food Addiction Scale (YFAS).[11] The YFAS adapts the DSM criteria for 'substance-related and addictive disorders' to specific foods.[9] Developed in 2009[2] and revised in 2016 (YFAS 2.0)[11] according to the DSM-5 criteria, this psychometric tool assesses the presence of 11 symptoms of addictive eating. Symptoms include craving, loss of control, tolerance and withdrawal associated with eating behaviours, the repeated unsuccessful attempts to reduce the consumption of specific foods and maintenance of these behaviours despite adverse physical/emotional/social/interpersonal consequences.[11] The YFAS 2.0 provides two scoring options: a continuous symptom score, reflecting the number of endorsed addiction-like symptoms; and a dichotomous diagnosis of 'food addiction'.[11] Using this self-report survey, approximately 15-20% of the adult population endorse ≥ three YFAS symptoms for addictive eating. [12-14]

Higher prevalence rates of addictive eating have been reported in individuals with higher Body Mass Indexes (BMI) classified as overweight or obese compared to lower BMIs.[13, 15] Although addictive eating is not exclusive to those with higher weight status.[16] It has been suggested that addictive eating in those with underweight may be related to dietary restriction practices (e.g., consuming more than intended that breaches self-imposed dietary rules,

For peer review only

intense craving resulting from extreme dieting practices).[16, 17] Irrespective of weight status, results from recent research indicate that individuals with addictive eating have significantly lower diet quality and higher intakes of highly processed foods.[14, 18, 19] Poor diet is a significant contributor to early death globally[20] and addressing addictive eating may contribute to the prevention or management of adverse health outcomes.

Addictive eating is a complex issue often overlapping with other health conditions, and likely transdiagnostic.[21, 22] There is evidence that addictive eating commonly co-occurs with mental health co-morbidities, particularly depression and anxiety, as well as overlapping with eating disorders, specifically binge eating disorder (BED).[12, 19] Approximately 50% of individuals with BED meet criteria for 'food addiction' according to the YFAS.[12] The present state of the literature demonstrates there is considerable overlap between BED and addictive eating.[23, 24] Commonalities include the loss of control over consumption, continued overuse despite negative consequences, and repeated failed attempts to reduce consumption.[24] However, differences have been observed. In BED large amounts of food are consumed over discrete periods of time, and caloric intake can vary markedly from day to day depending on the number of binge episodes experienced.[25, 26] Whereas, addictive eating behaviours in some can occur over the entirety of a day.[27] Anecdotally, individuals with addictive eating often report the quantity of food consumed remains consistent from day to day. Further, individuals with addictive eating report preferences for specific foods, typically those high in added fat, and/or refined carbohydrates,[18] and not on the consumption of all foods in general as may be presented in BED. At this time, it is unclear if addictive eating will emerge as a severe subtype of BED or be regarded as a distinct form of an addiction disorder. This distinction will be important to allow for targeted treatment and prevention strategies in susceptible individuals.

Current treatment options for addictive eating largely stem from online self-help groups such as Food Addicts Anonymous[28] and Overeaters Anonymous[29] which have 10 000+

For peer review only - http://bmjopen.bmj.com/site/about/guidelines.xhtml

members and have been in existence for many years, demonstrating a need for services.[30] A 2021 systematic review[31] found there is limited evidence supporting implementation of feasible and effective dietary interventions run by clinicians, for the management of addictive eating.[32] Of the nine studies reviewed, five interventional studies were found to improve symptoms of addictive eating[31] as indicated by improvements in YFAS[2, 11] outcomes. These interventions included medication (combination of naltrexone and bupropion,[33] as well as pexacerfont[34]), bariatric surgery[35, 36] and lifestyle modification.[37] Since publication of this review, a further four intervention studies (a behavioural weight loss program, [38] a brief telephone-based cognitive behavioural therapy intervention,[39] a low carbohydrate dietary program,[40] and a probiotic supplement placebo-controlled trial[41]) have been trialled. All four studies reported an improvement in YFAS addictive eating symptomatology immediately following the intervention, but two studies[38, 39] noted this improvement was not sustained over the longer-term. To date, most studies have been limited in sample size and therefore not been powered to detect a change in addictive eating symptoms.[31] Given the limited number of treatment options for addictive eating, there is a clear need for services, and development and testing of interventions. It has been suggested that interventions based on substance use addiction models may be effective at facilitating changes in eating behaviour.[42]

Motivational interviewing (MI) based interventions for addictive disorders, such as alcohol use, that contain coping skills training for traits associated with risk of addictive behaviour, have found to be effective.[43, 44] The traits that have been linked to addictive eating include impulsivity, sensation seeking, and anxiety and depression proneness.[45-49] Findings suggest that individuals with addictive eating may be highly aware of emotions, but lack the skills needed to cope with negative affect.[50] Using personalised coping skills for traits associated with personality and the risk of addictive behaviour in combination with MI, a communication approach used to identify and resolve ambivalence between desired behaviors and actual behaviors to increase motivation,[51] may be affective to facilitate

For peer review only - http://bmjopen.bmj.com/site/about/guidelines.xhtml

behaviour change in individuals with addictive eating. This project builds on a program of work that included an initial feasibility study for the management of addictive eating in adults (Australia New Zealand Clinical Trial Registry ACTRN12619001540101).[32] Results from the initial study indicated that the program was feasible in the target population. Feedback, received from program participants and facilitators, identified a need for a greater number of program sessions and improved strategies for increasing retention. As a result, the program was further refined with consumers using an integrated knowledge translation (iKT) framework.[52] This co-design phase included consumers with lived experience, as well as health professionals from a range of disciplines to ensure the culmination of multidisciplinary evidence-based strategies were included. This was unique as previous reports omit this co-design step or are siloed in their approach.[52] The co-design process used a series of interviews and workshops to gain input into the program overview, aims, content and materials. Subsequent changes were made to the program content, language used, and materials were created or refined to improve acceptability. The resultant behaviour change intervention, the TRACE (Targeted Research on Addictive and Compulsive Eating) program, is a complex intervention and previously described using the Medical Research Council TiDier (Template for Intervention Description and Replication) checklist for complex interventions.[53] (See [52] for the TIDieR checklist of the intervention).

To the authors knowledge, the TRACE program is the first MI-based telehealth intervention involving personalised coping skills training for the management of addictive eating in adults. Telehealth has been shown to be a strategy to increase reach, with virtual sessions being comparable to face-to-face programs, and to increase access to services without compromising effectiveness.[54] Telehealth will allow participants from anywhere in Australia to participate from home, and will reduce the demands on time and cost of travel.[54] Additionally, telehealth may overcome client-centred barriers by allowing a safe atmosphere for some participants to better engage and discuss more sensitive topics that they would not normally raise.[55]

For peer review only

For peer review only - http://bmjopen.bmj.com/site/about/guidelines.xhtml

The aim of the current study is to determine the efficacy of a telehealth intervention (active intervention) to reduce symptoms of addictive eating in adults, relative to passive intervention and control (no intervention) groups. It is hypothesised that both the active and passive intervention groups will achieve a reduction in addictive eating symptoms relative to the control group. Potential moderators (e.g., participant sociodemographics) and mediators (e.g., physical activity, diet, and sleep) of intervention efficacy will also be evaluated.

METHODS

Study trial design

The TRACE program is a randomised controlled trial with three parallel arms (n=50 per group). The primary outcome is the change in addictive eating symptoms at the 3-month post-baseline assessment (primary time point). The study also includes a 6-month post-baseline follow-up assessment. This project was approved by The University of Newcastle Human Research Ethics Committee (H-2021-0100) and prospectively registered with the Australian New Zealand Clinical Trials Registry (ACTRN12621001079831). The study protocol was developed in accordance with the Standard Protocol Items: Recommendations for Interventional Trials (SPIRIT) guidelines.[56] The design, conduct and reporting of the studies will adhere to the CONSORT (Consolidated Standards of Reporting Trials) guidelines.[57] All participants will provide informed electronic consent (see Supplementary Material 1 for a copy of the consent form) to participate and can withdraw at any time for any reason. The funding bodies had no role in the design, conduct or reporting of the study.

Setting

The active intervention will be delivered via telehealth sessions, conducted in Australia, and supported by a program workbook, and website containing materials relevant to the intervention.

For peer review only - http://bmjopen.bmj.com/site/about/guidelines.xhtml

Recruitment

Participants will be recruited using a range of strategies including media releases, advertising via local and national newspapers, and social-media releases. Informed by our iKT process, a range of recruitment videos (tailored for gender) were also created in addition to written material which will be released via Twitter and Facebook. A non-probability sampling technique (voluntary response sampling)[58] will be used, and recruitment will continue until the desired number of participants is achieved. Recruitment commenced in August 2021 and was completed in April 2022. Recruitment materials will direct individuals to the study information sheet and eligibility survey. The eligibility survey takes approximately 15 mins to complete (Table 1). Online informed consent will be obtained prior to completing the eligibility survey.

Eligibility

To be eligible for inclusion in the study individuals must:

1. Be aged between 18 years and 85 years
2. Endorse ≥ 3 symptoms on the Yale Food Addiction Scale 2.0 (i.e. exhibiting mild to severe addictive eating)[11]
3. Have a self-reported weight and height consistent with a body mass index (BMI) ≥ 18.5 kg/m^2
4. Be competent in the English language
5. Live in Australia
6. Have access to the internet

Individuals will be excluded from participating in the study if they:

1. Are pregnant or lactating
2. Report having a severe mental illness (including schizophrenia or bipolar disorder) or have a health condition that necessitates taking medications which affect dietary intake or weight status

For peer review only - http://bmjopen.bmj.com/site/about/guidelines.xhtml

3. Report purging behaviours as identified by the Eating Disorder Examination Questionnaire – Short form (EDE-QS)[59]

Methodological considerations for eligibility criteria: The eligibility screener excludes individuals with a BMI below 18.5kg/m^2. This measure was put in place to reduce the likelihood of recruiting participants with at-risk restrictive eating practices that may be influencing a relatively low weight status. The value of <18.5 kg/m^2 was chosen as this is below the current healthy weight range in national guidelines for Australians[60] and The Centre of Disease Control and Prevention (CDC) in the USA.[61] Additionally, the eligibility screener includes the Eating Disorder Examination Questionnaire Short Form (EDE-QS).[59] This 12-item validated tool is commonly used to identify potential eating disorders. Based on the research team consensus, individuals who have compensatory behaviours such as bingeing/purging (specifically asked in question 7 on the EDE-QS), who may be at risk of an eating disorder and are medically compromised, will be deemed not eligible for the current study. Purging is related to higher levels of appearance dissatisfaction, anxiety and depressive symptoms and self-concept instability.[62, 63] As per the ethics protocol, participants endorsing any response to this question, indicating these compensatory behaviours will be excluded from the study. The tools for eating disorders and psychological health[59, 64-68] used in the study have been widely used in research in the areas of eating disorders, dietary interventions, substance use and mental health and are considered standard tools for their specific measures. Study information as well as at completion of surveys participants are provided with contact information if they experience or further assistance with health behaviours.

Study procedure

Prospective participants will complete the eligibility survey. This will include demographic questions (e.g., sex, postcode, marital status, level of education, employment status); the Yale Food Addiction Scale 2.0[11] to confirm endorsement of ≥ 3 addictive eating symptoms; the EDE-QS[31] to confirm the absence of purging behaviours. While not necessary to determine

For peer review only - http://bmjopen.bmj.com/site/about/guidelines.xhtml

Table 1. Schedule of measurements

Variable	Instrument	Enrolment	Timepoint post allocation		
**Primary study**		Eligibility Screening	t_1 Baseline	t_2 3-months	t_3 6-months
**Sample characteristics**					
Demographics	Age, sex, postcode, mental health status	✓			
Socioeconomic factors	Education, income, marital status, employment status, occupation and living/accommodation status	✓			
Anthropometrics	Self-report height and weight	✓		✓	✓
Smoking and substance use	Alcohol, Smoking and Substance Involvement Screening Test - Version 3.0[64]		✓	✓	✓
Purging behaviours	Eating Disorder Examination Questionnaire Short form (EDE-QS)[59]	✓			
**Primary Outcomes**					
Addictive eating symptoms and severity	Yale Food Addiction Scale 2.0[11]	✓		✓	✓
**Secondary Outcomes**					
Dietary intake and quality	Australian Eating Survey[69, 70]		✓	✓	✓
Depression, anxiety and stress	Patient Health Questionnaire-8,[68] Generalized Anxiety Disorder 7,[65] Perceived Stress Scale[66]	✓		✓	✓
**Mediators/moderators**					
Trait/s associated with risk of addictive behaviour	Substance Use Risk Profile Scale[71]		✓		
Eating Behaviours	Eating Disorder Examination Questionnaire 6.0,[67] Binge Eating Scale,[72] Short Inventory of Grazing,[73] Reward-Based Eating Drive Scale[74]		✓	✓	✓
Participant activation level	Patient Activation Measure 13[75]	✓		✓	✓
Usage and engagement with program website	Google Analytics (Google LLC) to record number of site visits, visit durations, number of page views, and links accessed/resources downloaded		◆———	———	———◆
Usage and engagement with Facebook group	Number of participants to join group; number of views, likes and comments per post manually recorded			◆———	———◆
**Other outcomes**					
Quality of life	EQ-5D-5L[76]		✓	✓	✓
Physical activity level	Active Australia Survey[77]		✓	✓	✓
Sleep hygiene behaviours	Pittsburgh Sleep Quality Index[78]		✓	✓	✓
Health care utilisation	Consumer Services Receipt Inventory[79]		✓	✓	✓
'Control' and 'Compulsion' associated with addictive eating	Qualitative analysis of a segment of the first telehealth session		✓		

Eligibility Screening = assessment of inclusion/exclusion criteria, Baseline = pre-intervention, 3-months = immediate post-intervention, 6-months = 3-months post-intervention.

For peer review only - http://bmjopen.bmj.com/site/about/guidelines.xhtml

eligibility, the Patient Health Questionnaire-8 (PHQ-8),[68] Generalized Anxiety Disorder-7 (GAD-7),[65] Perceived Stress Scale-4,[66] Patient Activation Measure 13,[75] and two questions relating to previous treatments sought for addictive eating, will also be completed by potential participants. These questions have been specifically added to extend our previously reported research[80] regarding the types of individuals recruited into interventions for addictive eating.

Participants deemed eligible will proceed to the online consent form (Figure 1. Overview of study schedule). Participants will be given a two-week period to consider participation. After this time, a member of the research team will contact any individuals via email who have not completed the consent form to determine their interest in participating. Following this, no other contact will be made. Participants who provide electronic written consent will complete the baseline assessment surveys measuring dietary intake and eating habits, traits associated with personality and risk of addictive behaviour, quality of life and healthcare service utilisation (Table 1. Schedule of measurements). The surveys take approximately 40 minutes to complete. On completion of baseline surveys, participants will be randomly allocated to one of three groups (Group 1: active intervention; Group 2: passive intervention; or Group 3: control; see *Intervention* description) and informed of their group allocation via email.

Figure 1. Overview of the study schedule

Following randomisation (see *Randomisation and Blinding*), a member of the research team will contact participants in Group 1 via telephone or email to arrange an appointment time for their initial telehealth session. Groups 1 and 2 will be emailed a copy of the program workbook (printable and fillable PDF versions); a hard copy is available for participants on request; and be provided with password protected access to the program website at this time. Telehealth sessions 2 – 5, for participants allocated to the active intervention group (Group 1), will be arranged during their first telehealth session.

For peer review only - http://bmjopen.bmj.com/site/about/guidelines.xhtml

Participants from all three groups will receive results from the eligibility and/or baseline surveys by the research team via email. On survey completion, Groups 1 and 2 will receive feedback on dominant trait/s that may be associated with increased risk for addictive behaviours (e.g., anxiety-proneness, impulsivity-proneness); symptoms of addictive eating; dietary, caffeine and alcohol intake; sleep hygiene and physical activity levels. At this timepoint, Group 3 will only receive feedback on dietary intake via email. At 6-months post study commencement, Group 3 will be provided with feedback on trait/s associated with personality and risk of addictive behaviour; symptoms of addictive eating; sleep hygiene and physical activity levels, along with access to the workbook and website (the passive intervention that Group 2 received at baseline). To ensure consistency across participants, email templates and standardised reports will be used by the research team. Group 2 will be guided with written instructions in their workbook on how to utilise their survey results to allow personalised goal setting regarding their dietary intake and eating patterns.

The primary and secondary outcomes will be assessed at 3-months (primary endpoint, immediate post-active intervention period) and 6-months (follow-up) where participants will complete post-program surveys (Table 1. Schedule of measurements). Participants will be sent reminder emails to complete their surveys. They will be reminded a maximum of three times at each time point. If no contact is received after such time, no further contact will be made. Participants will be remunerated with a gift voucher to the value of AUD20 at the completion of baseline, 3-month and 6-month surveys, corresponding to a maximum of AUD60 per participant over the course of the study.

Randomisation and Blinding

Following completion of baseline assessments, participants will be stratified into 4 groups by sex and mental health status (presence or absence, based on either depression, scale PHQ-8 scores ≥15 or below 15, or anxiety scale GAD-7 scores ≥11 or below 11). Participants within

For peer review only - http://bmjopen.bmj.com/site/about/guidelines.xhtml

each of these four groups will be randomised to one of the three study groups in equal ratios using permuted block randomisation, with block sizes of six. Randomisation will promote group balance on these variables shown to be important in past cross-sectional research (for example, [12, 13, 15, 80]). The randomisation sequence will be generated by an independent statistician and implemented by a designated study co-ordinator. The allocation list will be stored in a password protected computer file and accessed only by the study co-ordinator.

Due to the telehealth nature of the active intervention, blinding of participants and dietitians to intervention group allocation in this study will not be possible. However, several strategies will be employed to reduce the risk of bias. First, participants will only be provided with partial information on the study hypotheses. Second, all communication between participants and research staff during the period of intervention (i.e., scheduling concerns, questions regarding the intervention) will be done directly between participants and the 'study co-ordinator' or their respective 'telehealth clinician'. Lastly, statistical analyses will be conducted by researchers who are blind to group allocation prior to analysis.

Intervention

The intervention study arms are:

- Group 1. Active intervention: targeting change in addictive eating behaviours using a multicomponent clinician led approach (telehealth sessions, program workbook and program website)
- Group 2. Passive intervention: targeting change in addictive eating behaviours using a multicomponent self-guided approach (program workbook and program website)
- Group 3. Control: dietary feedback, via paper-based report, provided at baseline and participants follow their usual dietary pattern for six months.

The comparator groups were chosen to provide a passive delivery option of the program which would be consistent with a self-guided Cognitive Behaviour Therapy approach (Group 2), and

For peer review only

a control group consistent with a standard version of dietary feedback (Group 3). The control group is not a wait list control, however participants in this group will be offered access to the passive intervention (i.e., program workbook and program website) after the completion of the 6-month assessment.

TRACE Active Intervention (Group 1): Participants will receive five standardised one-on-one telehealth/phone sessions with an Accredited Practising Dietitian, with training in behaviour change and eating disorders, over a 3-month period (i.e., weeks 1, 2, 4, 8 and 12). Additionally, dietitians leading the intervention delivery will have extensive experience in private practice work and working with clients including those with disordered eating and those with mental health conditions. Sessions will range from 15-45 mins. Telehealth sessions will be provided via the VSee platform (www.vsee.com). The active intervention uses personalised feedback, skill-building exercises, and goal setting to help individuals reduce their symptoms of addictive eating and improve their dietary intake, and relationship with food (see Table 2 for *Overview of intervention sessions*). The intervention is personalised based on an individual's dominant trait/s associated with personality and risk of addictive behaviour (i.e., the traits: depression proneness, anxiety proneness, sensation proneness and/or impulsivity proneness; measured via The Substance Use Risk Profile Scale[37] which the individual scores the most highly for) and addresses a range of factors that influence behaviour, both internal and external. Further, dominant trait/s associated with personality and risk of addictive behaviour are mapped to specific coping skill strategies which are in turn incorporated into the goal setting process. As part of session 1, the first 15 mins of the consultation will be audio recorded to enable qualitative analysis of responses to standardised questions regarding two elements of 'control' and 'compulsion' around the participant's food intake. These two themes were previously identified, through thematic analysis of the feasibility study data,[81] as having an influential relationship with addictive eating behaviours. On completion of the five telehealth sessions, participants will be invited to join a closed Facebook group from 3-months post

For peer review only - http://bmjopen.bmj.com/site/about/guidelines.xhtml

commencement of the intervention until the 6-month outcome survey measures are conducted. Joining the Facebook group is voluntary.

Table 2. Overview of intervention sessions

Session	Session aims
**1) Personality (Week 1: 45 mins)**	• Introduce the intervention<br>• Determine participant's main concerns with their food intake<br>• Provide feedback on baseline scores of addictive eating<br>• Discuss what this means when attempting and preparing to make changes<br>• Provide feedback on traits associated with personality and risk of addictive behaviour<br>• Discuss how personality traits may relate to food intake and addictive eating, and what this means for the individual<br>• Discuss coping strategies based on personality traits and complete '*Urge Surfing*' activity<br>• Introduce '*Distraction List*'<br>• Set homework task: choose and practice 2 coping strategy exercises<br>• Provide session summary
**2) Food (Week 2: 45 min)**	• Review session 1<br>• Check in for episodes of overeating<br>• Discuss progress with homework task - coping strategies<br>• Provide feedback on dietary intake<br>• Discuss core vs non-core food intake (Optional: discuss alcohol intake)<br>• Develop 3 nutrition goals using *SMARTER Goal Checklist*<br>1) Positive – increase core foods<br>2) Reduction – decrease non-core foods<br>3) 'Eating awareness' – using strategies to delay or halt overeating<br>• Discuss enablers/barriers when making changes to eating habits<br>• Discuss '*No Money No Time*' website (www.nomoneynotime.com.au)<br>• Discuss '*Practical Strategies to Achieve Goals*'<br>• Set homework task: complete '*Triggers for Overeating Checklist*'

For peer review only

For peer review only - http://bmjopen.bmj.com/site/about/guidelines.xhtml

Session	Session aims
	• Provide session summary
**3) Skills (Week 4: 30 min)**	• Review session 2<br>• Assess progress with SMARTER goals<br>• Check in for episodes of overeating<br>• Discuss homework task - '*Triggers for Overeating*'<br>• Explore strategies to overcome triggers, building on previous coping strategies and '*Practical Strategies to Achieve Goals*'<br>• Discuss and determine a '*food line*' to identify when eating is no longer enjoyable or not tasting food<br>• Discuss strategies to stay below the 'food line'<br>• Set homework task: complete '*Mood Monitor'* worksheet<br>• Provide session summary
**4) Confidence (Week 8: 30 min)**	• Review session 3<br>• Discuss progress with plan to stay below 'food line' and for episodes of overeating<br>• Explore enablers/barriers to achieving goals<br>• Discuss homework task - '*Mood Monitor*', and explore emotions that participant has difficulty coping with<br>• Discuss seeing emotions differently<br>• Explore coping strategies for difficult emotions<br>• Discuss importance of sleep, physical activity, and responsible intake of alcohol for emotional health<br>• Discuss implementing coping skills plan to achieve SMARTER goals (i.e. consolidate information from sessions 1 – 4)<br>• Set homework task: practice implementation of coping skills plan to achieve goals<br>• Provide session summary
**5) Moving forward (Week 12: 20 mins)**	• Review session 4<br>• Check in/briefly problem solve and encourage participant to continue with goals and strategies<br>• Discuss topics from previous sessions (participant led)<br>• Reassess confidence to achieve goals<br>• Provide final *Addictive Eating Action Plan*<br>• Discuss how support group on Facebook works and encourage sign up

404

For peer review only - http://bmjopen.bmj.com/site/about/guidelines.xhtml

Participant Workbook and Program Website: Participants will have access to a participant workbook and password protected access to a study specific website, both built for the study to support the materials discussed in the intervention sessions. To further facilitate the co-design process, the workbook and website content was piloted with end users (n=2) with lived experience of addictive eating, who participated in the iKT interviews/workshops. The end users reported the workbook and website to be highly usable in terms of the content, and the language used throughout as appropriate with only minor modifications made. Additionally, the piloting process allowed the estimated time to complete each workbook module to be calculated.

Program Workbook: The workbook consists of five modules: 1) Personality; 2) Food; 3) Skills; 4) Confidence; and 5) Moving forward. The content of the five modules mirrors that of the telehealth sessions. The workbook also contains reflective activities/worksheets, discussed during the telehealth sessions, for the participants to complete. These elements were deemed important during the iKT process. The amount of time spent completing activities in the workbook each week, between telehealth sessions, will take approximately 30 - 60 minutes. However, the time to complete each module may vary from person to person, and participants are advised to work through the workbook at a pace that is right for them.

Program Website: The website includes the following pages: 1) Home/Landing page: brief information about the program and login; 2) Dashboard: navigation page to access each of the program's module pages; 3) Module pages: each of the five modules within the intervention has a separate page on the website. This includes additional resources to complement the telehealth sessions and workbook; and 4) About us: brief information about the research/clinician team behind the program, including contact information (email). The website will be available for a period of 12 months from study commencement. All data captured from the website will be encrypted and stored securely on a server.

For peer review only

For peer review only - http://bmjopen.bmj.com/site/about/guidelines.xhtml

Program Facebook Group: This is a voluntary part of the study which aims to further support participants with behaviour change. The Facebook forum is set up as a private Facebook group. Participants can use their standard Facebook login, or alternatively, create a new login (a pseudo account) that does not identify them if they wish to remain anonymous. Participants will be prompted with information related to the intervention for the 3-month duration in the form of short posts, blogs, and polls. The Facebook group will allow participants to engage with other participants from the program, as well as serve as a communication method to remind participants about assessments for the study.

The Facebook forum has the following restrictions: 1) Membership will be by invitation only; 2) The group will not appear in search results or the participants Facebook profile; and 3) Only group members will be able to see the group information and group posts. Participants will be advised of the appropriate use of language and etiquette for using the social media/discussion forum in the workbook and reminded at the final telehealth session. The Facebook group will be moderated by a member of the research team via the TRACE research Facebook account.

Intervention fidelity: A detailed clinician manual will be used by the dietitian for all telehealth sessions to maintain treatment fidelity. Dietitians administering the intervention will be trained by the principal investigator prior to study implementation. Dietitians will also follow each session as outlined in the manual and keep a dietitian log of participants telehealth sessions. Further, five participants allocated to Group 1, with their consent, will have all their telehealth sessions audio recorded. The dietitian log and audio recordings will be reviewed by an independent researcher to ensure the intervention was delivered as intended. Regular supervisory meetings will be conducted with the dietitians and program coordinator led by the principal investigator. Participant adherence to the intervention will be assessed by a session attendance checklist completed by a member of the research team. Dietitians administering the telehealth sessions will monitor completion of homework tasks and workbook activities at the start of telehealth sessions 2 to 5. Assistance will be provided by the dietitian at this time

For peer review only - http://bmjopen.bmj.com/site/about/guidelines.xhtml

if participants experienced any difficulties completing the homework tasks/activities. Additionally, to assist with adherence, on completion of each telehealth session the dietitian will email a personalised 'Addictive Eating Action Plan', completed on a standardised template, to the participant.

TRACE Passive Intervention (Group 2): Participants will receive the intervention via self-guided approach, with access to the five-module workbook and website (as described above), but without the telehealth consults. The content of the workbook modules mirrors the content of the five telehealth sessions. In addition to the written materials provided, the workbook contains spaces for reflective activities, documenting goals and monitoring progress. Participants will be asked, on receipt of the workbook, to complete the workbook within a 3-month period. The proportion of the workbook completed by participants in the passive intervention arm will not be monitored. Following the 3-month self-guided learning period, participants will be invited to join the closed Facebook group as described above.

Control (Group 3): Participants will receive personalised dietary feedback on baseline surveys, provided by an automated report, generated from the Australian Eating Survey. This is consistent with standard dietary feedback from a dietitian. Participants in the control group will be offered access to the participant workbook and study website after the completion of the 6-month assessment.

Patient and public involvement

Consumer (i.e., individuals with lived experience of addictive eating who participated in the pilot study) input was received on the pilot version of the intervention (FoodFix process evaluation[32]) that directly guided the enhancement of the TRACE telehealth sessions. The TRACE program workbook and website for the current study was developed following the pilot study. A sample of consumer representatives (individuals with lived experience of addictive eating and healthcare experts including clinicians and managers), independent of those

For peer review only - http://bmjopen.bmj.com/site/about/guidelines.xhtml

involved in the pilot study, were involved in the review of the program and program materials.[52]

Consumer representatives were interviewed to:

- Identify what individuals with addictive eating need and want more accurately
- Gather information about what works well and what needs improving, first-hand from consumers who may use them
- Openly consider different or opposing views about aspects of the research project
- Test resources during development and refine resources making sure they will work well in practice
- Detect any unforeseen consequences of a particular decision or direction that has been made regarding the project
- Gain support of consumers to implement changes to the research project

The opinion of consumers has been considered to create a program that:

- Aligns to the needs of the people it is trying to help i.e., individuals with addictive eating
- Is beneficial in terms of delivering meaningful outcomes for individuals with addictive eating
- Is conducted in a way that is sensitive to peoples' needs

Consumers were not involved in the design of the current study, the selection of outcome measurements, research questions or the recruitment of additional participants. However, consumers were involved in the overall concepts employed in the study and may be called upon at the dissemination stage. For example, to review plain language summaries of the results, provide advice on ways to communicate/translate our findings, or present our findings to the community. Participants of the current study can request a plain English summary of the study outcomes on its completion.

For peer review only - http://bmjopen.bmj.com/site/about/guidelines.xhtml

Outcome measures

All outcome measures are completed at baseline, 3 months (immediate post-active intervention period) and 6 months (follow-up) via online surveys. The same survey tools will be used at each time point. Participants will receive assessment reminders by email. (Reference to where data collection forms can be found is included in Supplementary Material 2)

Primary outcomes

Addictive eating symptoms and severity: The Yale Food Addiction Scale (YFAS 2.0)[11] will be used to assess the change in addictive eating symptomatology and severity. The YFAS 2.0 is a validated self-report 35-item questionnaire. The YFAS 2.0 asks participants to think of specific foods they have had difficulty controlling the consumption of within the past 3 months (e.g., ice cream, chocolate, chips, hamburgers). The YFAS 2.0 provides an addictive eating symptom score ranging from zero to 11. Additionally, two items assess clinically significant impairment or distress from eating. A 'food addiction diagnosis' can be given when ≥2 symptoms are endorsed, and clinically significant impairment or distress is present. However, for the purpose of this study a 'food addiction diagnosis' will not be given, and severity of addictive eating will be classified in accordance with YFAS scoring instructions as follows: "mild" = 3 symptoms, "moderate" = 4-5 symptoms or "severe" ≥6 symptoms. The YFAS 2.0 has been found to be a robust and psychometrically sound measure of addictive eating symptomatology in non-clinical[2, 82] and clinical populations with good test/retest validity.[83] Preliminary evidence[31, 32] suggests that YFAS scores are sensitive to change and are decreased after intervention.

Secondary outcomes

Dietary intake and quality: Changes in dietary intake and quality will be measured using the Australian Eating Survey (AES).[69] The following dietary outcomes will be measured: (1) core foods and non-core foods percentage contribution to total energy intake; (2) average daily

For peer review only - http://bmjopen.bmj.com/site/about/guidelines.xhtml

energy intake, proportion of total energy intake contributed by macronutrients, micronutrient intakes; and (3) overall diet quality. The AES is a validated 120-item semi-quantitative Food Frequency Questionnaire that assesses usual food and nutrient intakes over the previous 3-6 months. The AES includes a comprehensive list of foods, including drinks, milk and dairy foods, breads and cereals, sweet and savoury snacks, main meals, other foods, vegetables and fruit. Frequency response options for each food, or food type, range from 'never' to '≥7 times per day'. The AES has been assessed for comparative validity relative to weighed food records and for fruit and vegetable intakes using plasma carotenoids.[69, 70] Standard portion sizes for adult men and women have been determined for each AES item in the survey, using data from the most recent Australian National Nutrition Survey. The food and beverage weight per serving, used in the calculation of food group servings (as serves per day) is consistent with sizes specified in the Australian Guide to Healthy Eating.[69, 70, 84] Nutrient intakes from the AES Food Frequency Questionnaire were computed using data in the AUSNUT 2011–13 database.[85] The AES also provides an Australian Recommended Food Score (ARFS), derived from a subset of 70 AES questions, as a measure of diet quality that reflects the overall healthiness and nutritional quality of an individual's usual eating pattern.[70] The ARFS is based on the frequency of consumption of core foods, recommended in the Australian Dietary Guidelines,[86] with foods awarded one point for a consumption frequency of ≥once per week. The total score is calculated by summing the points for each item and scores can range from zero to 73, with higher scores awarded for greater dietary variety.[70]

Depression, anxiety and stress: Changes in symptom scores for depression, anxiety and stress will be measured using the Patient Health Questionnaire (PHQ-8),[68] the Generalized Anxiety Disorder 7 (GAD-7)[65] and the Perceived Stress Scale (PSS-4),[66] respectively. The PHQ-8 is a validated self-report 8-item tool that asks the individual to rate the severity of depressive symptoms over the past two weeks from 0 ('not at all') to 3 ('nearly every day'). Total scores for the 8 items range from 0 to 24, and severity will be determined using the following cut-offs: 0-4 = minimal, 5-9 = mild, 10-14 = moderate, 15-19 = moderately severe,

For peer review only - http://bmjopen.bmj.com/site/about/guidelines.xhtml

and 20-24 = severe.[68] The GAD-7 is a validated self-report 7-item tool that asks the individual to rate the severity of symptoms over the past two weeks from 0 (‘not at all sure’) to 3 (‘nearly every day’). GAD-7 total scores range from 0 to 21, and severity is determined using the following cut-offs: 0-5 = mild, 6-10 = moderate, 11-15 = moderately severe, and 15-21 = severe.[65] The PSS-4 is a validated self-report 4-item tool that assesses the degree to which a person perceives life as stressful.[66] The questions have been designed to assess how unpredictable, uncontrollable, and overloaded a person feels their life to be. Frequency over the previous month is rated on a five-point Likert scale ranging from ‘Never’ to ‘Very often’. PSS-4 total scores range from 0 to 16, and higher scores indicate greater stress.[66] Currently, there is no established cut-off for the PSS-4 score to indicate adverse levels of stress.

Other outcomes

A selection of other outcomes was chosen based on co-occurring health conditions (see Table 1 for schedule of measurements).

Quality of Life: Changes in subjective quality of life will be measured using the EQ-5D-5L.[76] The EQ5D-5L is a validated self-report 5-item tool to assess health-related quality of life. A descriptive system comprising five dimensions: mobility, self-care, usual activities, pain/discomfort, and anxiety/depression. Each dimension has 3 levels: no problems, some problems, and extreme problems (labelled 1–3). Participants are asked to indicate their health state by ticking the box next to the most appropriate statement in each of the five dimensions. This decision results into a 1-digit number that expresses the level selected for that dimension. The digits for the five dimensions can be combined into a 5-digit number that describes respondent’s health state. The EQ-5D-5 L will be analysed to produce an index score between 0 (state of death) and 1 (perfect health).

Physical activity level: Changes in physical activity level will be measured using the Active Australia Survey (AAS).[77] The AAS is a validated self-report tool containing eight core

For peer review only - http://bmjopen.bmj.com/site/about/guidelines.xhtml

questions to assess participation (hours/mins per week) in moderate and vigorous intensity physical activity and walking for recreation, over the previous week.

Sleep hygiene behaviours: Changes in sleep hygiene behaviours will be measured using the Pittsburgh Sleep Quality Index (PSQI).[78] The PSQI is a validated self-report survey with 19 self-rated items and 5 items rated by the bed partner or roommate (if applicable). The tool assesses seven components of sleep to provide one global score. Components measured include 1) Subjective sleep quality, 2) Sleep latency, 3) Sleep duration, 4) Habitual sleep, 5) Sleep disturbances, 6) Use of sleeping medication, and 7) Daytime dysfunction. The overall global score of sleep quality will be calculated, and the subcomponents reported.

Health care utilisation: For the purpose of conducting a cost analysis the Consumer Services Receipt Inventory (CSRI)[79] will be completed by participants at each time point. The CSRI is an adaptable tool that ensures the format, language, scope and content is compatible with the research aims, context, participants' likely circumstances, and the quantity and precision of information required.[87] Health care utilisation is captured through self-report and includes information on the number of appointments and type of health care services used in the preceding 3 months.

Cost analysis: A cost-consequence analysis will be conducted including calculating the cost of each intervention (i.e., active, passive and control) and reporting intervention costs alongside mean change outcomes. Intervention costs will be recorded in terms of cost of intervention development, intervention delivery and the operating costs of the RCT. Outcomes to be reported as part of the cost analysis will include mean change in addictive eating symptom scores assessed using the YFAS (i.e. the primary outcome), as well as mean change in the number of health care appointments in the past 3-months assessed using the CSRI, and mean change in Quality Adjusted Life Years (QALYs) assessed using the EQ-5D-5L. This

For peer review only - http://bmjopen.bmj.com/site/about/guidelines.xhtml

approach was selected to provide a comprehensive and transparent overview of intervention costs, given the lack of cost analysis data in this area of research.[88, 89]

Mediators/Moderators

The following potential mediators and moderators of intervention efficacy will be examined:

Trait/s associated with personality and risk of addictive behaviour: Participant's will complete the Substance Use Risk Profile Scale (SURPS)[71] at baseline to determine their dominant trait/s. The SURPS is a validated self-report 23-item survey that assess four traits associated with increased risk for addictive behaviours (Impulsivity proneness, Sensation proneness, Depression proneness, and Anxiety proneness).

Eating behaviours: Eating behaviours that have been shown to have overlap with addictive eating will be measured. This includes eating disorders, binge eating, grazing behaviours and reward driven eating. Eating disorders will be measured using the Eating Disorder Examination Questionnaire 6.0 (EDEQ-6.0)[67] The EDEQ-6.0 is a validated self-report 28-item questionnaire that assesses the occurrence and frequencies of key eating disorder behaviours with cognitive subscales related to eating disorders (restraint, eating concern, shape concern, and weight concern) and behavioural symptoms related to these concerns (e.g. frequency of binge eating, vomiting, use of laxatives or diuretics, and overexercise). Subscale and global scores reflect the severity of eating disorder psychopathology. Binge eating will be measured using the Binge Eating Scale (BES).[72] The BES is a validated self-report 16-item questionnaire to assess the presence of certain binge eating behaviours, over the past 28 days, which may be indicative of an eating disorder. Each item contains 3-4 statements about behaviours, thoughts, and emotional states. Grazing behaviours will be measured using the Short Inventory of Grazing (SIG).[73] The SIG is a validated self-report 2-item measure to assess 1) the presence and frequency of grazing in general, and 2) the presence and frequency of grazing accompanied by a sense of loss of control. Reward driven

For peer review only

eating will be measured using the Reward-Based Eating Drive Scale (REDX-5).[74] The REDX-5 is a validated self-report 5-item questionnaire, in 5-point Likert scale format from 1 (strongly disagree) to 5 (strongly agree), that assesses reward-driven eating (loss of control over eating, lack of satiety, and preoccupation with food).

Participant Activation Level: Participant's underlying knowledge, skills and confidence in managing their addictive eating behaviours and overall health will be measured using the Patient Activation Measure (PAM-13).[90] The PAM-13 is a validated self-report 13-item scale that draws on concepts such as health locus of control, self-efficacy in managing health behaviours and readiness to change health behaviours.[75, 91] Higher PAM-13 scores indicate that individuals have higher levels of activation, and understand their role in the self-management process and feel capable of fulfilling that role.[92] Research has demonstrated that a single point change in PAM score is meaningful.[93]

Engagement and use of the program website and Facebook group: Interaction with the website will be objectively tracked throughout the study (baseline to 6 months i.e., timepoints 1 to 3) using Google Analytics (Google LLC). Measures of engagement and usage will include number of website visits, website visit duration, number of page views and links accessed/resources downloaded.

Interaction with the Facebook group will be measured throughout the post-intervention period (3 to 6 months from baseline i.e., timepoints 2 to 3). Measures of engagement and usage will include number of participants to join the Facebook group, and number of views, likes and comments per post.

Study sample characteristics

Sociodemographic data will be collected by online questionnaire at baseline. Participants will provide information on their age, sex, marital status, postal code, years of education,

For peer review only - http://bmjopen.bmj.com/site/about/guidelines.xhtml

employment status and current living situation. Index of Relative Socio-Economic Disadvantage (IRSD) score,[94] based on the Australian Bureau of Statistics census data and reflecting a proxy index of socioeconomic status, will be determined by postal code of residence. Current smoking and substance use will be measured using the Alcohol, Smoking and Substance Involvement Screening Test - Version 3.0.[64] Additionally, previous treatment sought for overeating from health professionals or products used to treat overeating will be collected.

Anthropometric data (self-reported height and weight) will be collected by online questionnaire at baseline. BMI will be calculated using standardised techniques and categorised according to the World Health Organization adult cut-off points.[95]

Sample size

The sample size for the study was calculated based on data from the feasibility study,[32] given the absence of other intervention studies. Through guidance with statisticians, a large effect size was chosen and needed to enable the possibility of a clinically meaningful result. A clinically meaningful difference in symptoms of addictive eating was selected as a decrease of 2 symptoms, given this would correspond to a change in severity classification on the YFAS 2.0 tool. To detect a mean 2-unit difference (SD = 2.2) in the YFAS symptoms between the active intervention group and the passive intervention group or control group and using a standardised effect size of d=0.91, a sample size of 32 individuals per group (total sample size n=96) is required to detect this change with a power of 0.90 and a type 1 error rate set at 0.025 to account for multiple testing. However, allowing for a 30% dropout rate from the pilot, a sample size of 46 individuals per group (total sample size n=138) would be required. Therefore, a total sample size of 150 individuals, with 50 per group, was chosen to remain conservative.

For peer review only - http://bmjopen.bmj.com/site/about/guidelines.xhtml

Statistical analysis plan

Data analysis will be conducted by a researcher blinded to the intervention conditions. Descriptive statistics of sample characteristics will be presented. For the primary YFAS outcome a Linear Mixed Model (LMM) will be based on a model with main effects for group (active intervention, passive intervention, control) and time (treated as categorical at levels baseline, 3 and 6 months), and the group-by-time interaction. An unstructured residual covariance structure will be used to allow for correlation between the repeated measurements for a subject. The primary outcome effect will be reported as the difference between means at baseline and 3 months, with a 95% CI for the difference. Mental health condition and BMI will be examined for possible moderating effects on the effect size, and if so adjustment for them will be carried out. Secondary descriptive analysis will be carried out to identify whether specific symptoms were predominantly associated with reductions in YFAS score.

A secondary outcome will be a categorical variable, clinically significant change from baseline to 3 months, where significant requires a reduction of 2 or more symptoms in the YFAS. This will be analysed using logistic regression with group being the only factor. Additional secondary outcomes will include dietary outcomes (average daily energy intake, proportion of total energy intake contributed by core foods and non-core foods intakes, macronutrients intakes, micronutrient intakes; and overall diet quality) and mental health status (depression, anxiety and stress scores). These will also be analysed using LMMs as per the approach above. All available data will be used with no imputation of missing values at 3 and 6 months, however baseline scores will be kept. The participants will be analysed according to their allocated randomisation group consistent with an intention-to-treat analysis. Statistical significance will be set at 0.05.

Data management and monitoring

Online survey data will be managed using REDCap electronic data capture tools[96, 97] hosted at the University of Newcastle. REDCap (Research Electronic Data Capture) is a

For peer review only - http://bmjopen.bmj.com/site/about/guidelines.xhtml

secure, web-based software platform designed to support data capture for research studies, providing 1) an intuitive interface for validated data capture; 2) audit trails for tracking data manipulation and export procedures; 3) automated export procedures for seamless data downloads to common statistical packages; and 4) procedures for data integration and interoperability with external sources.

All data captured from the study website will be encrypted and stored securely on the server. All other data collected will be entered into a password protected central database which is hosted on secure university-based servers, which comply with robust security standards for clinical data and are subject to daily backups and regular offsite backups. Only authorised members of the research team will have access to the database. Research staff handling study data are trained in procedures for handling sensitive information, accurate data entry, surveillance and intervention-specific data storage and data archive. Facilitators of the telehealth sessions are responsible for the electronic storage of study forms on the central database. All completed forms will be checked for completeness and accuracy, first by data collectors and later by a member of the research team responsible for data management. Throughout the study period (at 25% and 50% of required participants) approximately 5% of records will be randomly selected for data quality checks of accuracy and completeness by an independent reviewer.

A Data Safety Monitoring Board will not be established for this study as all elements of the intervention have been previously explored and used in interventions. To monitor for potential risks, the study co-ordinator managing the day-to-day conduct of the trial, and facilitators of the telehealth sessions, will report weekly to the Chief Investigator. Oversight concerning the overall conduct of the trial will be provided by our multi-disciplinary research team. This will include regular meetings to review protocol adherence, participant retention rates and safety reports. For the entire study period, any adverse events, of any kind, that might be related to either the trial intervention or trial procedures will be logged in an adverse event log and

For peer review only - http://bmjopen.bmj.com/site/about/guidelines.xhtml

reported to the Human Research Ethics Committee by the Chief Investigator. To maintain the welfare of participants, with their consent, relevant survey results from the GAD-7[65] and PHQ-8[68] will be sent to the participant's nominated General Practitioner/ health professional if they score in the severe category for either anxiety (GAD-7 scores ≥16) or depression (PHQ-8 scores ≥20) if participants consent to this disclosure.

Study sponsorship and organisation

The sponsor of the trial is the University of Newcastle, and funding was provided by the National Health and Medical Research Council (NHMRC). The trial will be conducted and evaluated independent of the study sponsor and funder. The study is coordinated independently of the study sponsor and funder, by researchers at the University of Newcastle, Australia with the study overseen by the trial management committee comprising the chief investigators.

Ethics and dissemination

The trial will be undertaken in compliance with the Declaration of Helsinki and approval to conduct the study was received from the University of Newcastle Human Research Ethics Committee (H-2021-0100). This trial adheres to the SPIRIT guidelines for randomised trials protocols[56] and the results will be reported in accordance with CONSORT guidelines (TIDieR checklist and guide[53]). Protocol modifications will be registered with the Ethics Committee and trial register. All participants will provide electronic consent to participate prior to completing the eligibility and baseline surveys. Results of the study will be disseminated via peer-reviewed publications and presentations at national and international conferences and will also form part of student dissertations. Data from the TRACE study may be made available in the future for collaborative research questions. Such requests must be authorised by the principal investigators and the appropriate Human Research Ethics Committees.

For peer review only - http://bmjopen.bmj.com/site/about/guidelines.xhtml

Limitations

Limitations of the study include the level of experience required of the dietitians administering the telehealth sessions, which may impact the scalability of the intervention. However, dietitians are highly trained professionals in behaviour change and extra care was taken given the uniqueness of the intervention. The fidelity outcomes assessed as part of the trial will provide important information regarding future implementation. Additional limitations include the exclusion of individuals with severe mental illnesses or complex health conditions. The current intervention is not designed for complex co-morbidities. It is envisaged that for these individuals a more complex care model is required where the TRACE program could be implemented alongside other approaches or treatments.

The TRACE program is designed to raise awareness, and support behaviour change, of addictive eating. If successful, our study will provide essential evidence regarding the efficacy of behavioural and dietary improvement in the management of addictive eating, thus allowing for the implementation of management strategies for addictive eating into community and clinical healthcare services. Further, if both the active and passive interventions are found to be effective it will provide evidence of different levels of care that could be utilised within these services.

Author Contributions: TLB conceptualised the study, and TLB, JAS, MW, ML, RC, KMP, AVG, PJH, ALB, LH, SJP, LGW, KC and CEC contributed to the study protocol. TLB, JAS, MW, ML, RC, KMP, AVG, PJH, ALB, LH, SJP, and CEC contributed to the intervention development and design, intervention resources and assessment methodology. JAS wrote the initial manuscript draft. TLB, JAS, MW, ML, RC, KMP, AVG, PJH, ALB, LH, SJP, LGW, KC and CEC contributed to the writing of the final manuscript and/or provided critical comments during revisions. All authors approved the final version prior to submission. TLB, JAS, MW, ML and RC will be responsible for recruitment, data collection and intervention delivery.

For peer review only - http://bmjopen.bmj.com/site/about/guidelines.xhtml

Competing interests: None declared.

Funding: This work was supported by the National Health and Medical Research Council (NHMRC) grant number [G1801414].

References

1. Davis C. From Passive Overeating to ‘‘Food Addiction’’: A Spectrum of Compulsion and Severity. *International Scholarly Research Notices Obesity.* 2013; 435027: 1-20. http://dx.doi.org/10.1155/2013/435027.
2. Gearhardt AN, Corbin WR, and Brownell KD. Preliminary validation of the Yale Food Addiction Scale. *Appetite.* 2009; 52(2): 430-436. https://doi.org/10.1016/j.appet.2008.12.003.
3. Gearhardt AN, Davis C, Kuschner R, et al. The Addiction Potential of Hyperpalatable Foods. *Current Drug Abuse Reviews.* 2011; 4: 140-145. doi: 10.2174/1874473711104030140.
4. Ruddock HK, Christiansen P, Halford JCG, et al. The development and validation of the Addiction-like Eating Behaviour Scale. *International Journal of Obesity (London).* 2017; 41(11): 1710-1717. https://doi.org/10.1038/ijo.2017.158.
5. Burrows T, Verdejo-Garcia A, Carter A, et al. Health Professionals’ and Health Professional Trainees’ Views on Addictive Eating Behaviours: A Cross-Sectional Survey. *Nutrients.* 2020; 12(9): E2860. https://doi.org/10.3390/nu12092860.
6. Whatnall M, Skinner J, Verdejo-Garcia A, et al. Symptoms of Addictive Eating: What Do Different Health Professions Think? *Behavioral Sciences.* 2021; 11(5): 60. https://doi.org/10.3390/bs11050060.
7. Gearhardt AN and Hebebrand J. The concept of “food addiction” helps inform the understanding of overeating and obesity: Debate Consensus. *The American Journal of Clinical Nutrition.* 2021; 113(2): 274–276. https://doi.org/10.1093/ajcn/nqaa345.

For peer review only

For peer review only - http://bmjopen.bmj.com/site/about/guidelines.xhtml

8. Hauck C, Cook B, and Ellrott T. Food addiction, eating addiction and eating disorders. *The Proceedings of the Nutrition Society.* 2020; 79(1): 103–112. https://doi.org/10.1017/S0029665119001162.

9. American Psychiatric Association, *Diagnostic and statistical manual of mental disorders*. 5th ed. 2013, Arlington: VA American Psychiatric Publishing.

10. World Health Organization. International statistical classification of diseases and related health problems (11th ed.). 2019. https://icd.who.int/.

11. Gearhardt AN, Corbin WR, and Brownell KD. Development of the Yale Food Addiction Scale Version 2.0. *Psychology of Addictive Behaviors.* 2016; 30(1): 113-121. https://doi.org/10.1037/adb0000136.

12. Burrows T, Kay-Lambkin F, Pursey K, et al. Food addiction and associations with mental health symptoms: a systematic review with meta-analysis. *Journal of Human Nutrition and Dietetics.* 2018; 31(4): 544-572. https://doi.org/10.1111/jhn.12532.

13. Pursey K, Stanwell P, Gearhardt A, et al. The prevalence of food addiction as assessed by the Yale Food Addiction Scale: a systematic review. *Nutrients* 2014; 6(10): 4552–4590. https://doi.org/10.3390/nu6104552.

14. Pursey KM, Collins CE, Stanwell P, et al. Foods and dietary profiles associated with 'food addiction' in young adults. *Addictive Behaviors Reports* 2015; 2: 41–48. http://dx.doi.org/10.1016/j.abrep.2015.05.007.

15. Skinner J, Jebeile H, and Burrows T. Food addiction and mental health in adolescents: a systematic review. *The Lancet. Child and adolescent health.* . 2021; 5(10): 751-766. https://doi.org/10.1016/S2352-4642(21)00126-7.

16. Sanchez I, Lucas I, Munguía I, et al. Food addiction in anorexia nervosa: Implications for the understanding of crossover diagnosis. *European Eating Disorders Review.* 2022; 30(3): 278–288. https://doi.org/10.1002/erv.2897.

17. Schulte EM and Gearhardt AN. Associations of Food Addiction in a Sample Recruited to Be Nationally Representative of the United States. *European Eating Disorders Review.* 2018; 26(2): 112–119. https://doi.org/10.1002/erv.2575.

For peer review only

For peer review only - http://bmjopen.bmj.com/site/about/guidelines.xhtml

18. Pursey KM, Skinner J, Leary M, et al. The Relationship between Addictive Eating and Dietary Intake: A Systematic Review. *Nutrients.* 2021(1): 164. https://doi.org/10.3390/nu14010164.

19. Burrows T, Hides L, Brown R, et al. Differences in Dietary Preferences, Personality and Mental Health in Australian Adults with and without Food Addiction. *Nutrients* 2017; 9 (3): E285. https://doi.org/10.3390/nu9030285.

20. GBD 2017 Diet Collaborators. Health effects of dietary risks in 195 countries, 1990-2017: a systematic analysis for the Global Burden of Disease Study 2017. . *Lancet (London, England),*. 2019; 393(10184): 1958–1972. https://doi.org/10.1016/S0140-6736(19)30041-8.

21. Treasure J, Leslie M, Chami R, et al. Are trans diagnostic models of eating disorders fit for purpose? A consideration of the evidence for food addiction. *European Eating Disorder Review.* 2018; 26(2): 83-91. doi: 10.1002/erv.2578.

22. Wiss DA and Brewerton TD. Incorporating food addiction into disordered eating: the disordered eating food addiction nutrition guide (DEFANG). *Eating and Weight Disorders.* 2017; 22(1): 49-59. doi: 10.1007/s40519-016-0344-y.

23. Burrows T, Skinner J, McKenna R, et al. Food Addiction, Binge Eating Disorder, and Obesity: Is There a Relationship? *Behavioral Sciences.* 2017; 7(3): pii: E54. https://doi.org/10.3390/bs7030054.

24. Bao K, French EN, Schleyer B, et al. Food addiction is associated with greater objective binge eating and eating disorder psychopathology, and higher body mass index in youth, a meta-analysis*. Psychiatry Research Communications.* 2022; 2(3): 100067. https://doi.org/10.1016/j.psycom.2022.100067.

25. Saunders R. "Grazing": A High-Risk Behavior*. Obesity Surgery.* 2004; 14(1): 98-102. doi: 10.1381/096089204772787374.

26. Dingemans A, Bruna M, and van Furth E. Binge eating disorder: a review. *International Journal of Obesity.* 2002; 26: 299–307. https://doi.org/10.1038/sj.ijo.0801949.

For peer review only

27. Gearhardt A, White M, and Potenza M. Binge Eating Disorder and Food Addiction. *Current drug abuse reviews.* 2011; 4(3): 210-207. doi: 10.2174/1874473711104030201.

28. Food Addicts Anonymous. [Homepage]. [cited 2022 24 March]; Available from: https://faacanhelp.org/.

29. Overeaters Anonymous. [Homepage]. [cited 2022 24 March]; Available from: https://oa.org/.

30. McKenna RA, Rollo ME, Skinner JA, et al. Food Addiction Support: Website Content Analysis. *JMIR Cardio.* 2018; 2(1): e10. https://doi.org/10.2196/cardio.8718.

31. Leary M, Pursey KM, Verdejo-Garcia A, et al. Current Intervention Treatments for Food Addiction: A Systematic Review. *Behavioral Sciences* 2021; 11(6): 80. https://doi.org/10.3390/bs11060080.

32. Burrows T, Collins R, Rollo M, et al. The feasibility of a personality targeted intervention for addictive overeating: FoodFix. *Appetite.* 2021; 156(104974). https://doi.org/10.1016/j.appet.2020.104974.

33. Carbone EA, Caroleo M, Rania M, et al. An open-label trial on the efficacy and tolerability of naltrexone/bupropion SR for treating altered eating behaviours and weight loss in binge eating disorder. *Eating and Weight Disorders.* 2021; 26(3): 779–788. https://doi.org/10.1007/s40519-020-00910-x.

34. Epstein DH, Kennedy AP, Furnari M, et al. Effect of the CRF1-receptor antagonist pexacerfont on stress-induced eating and food craving. *Psychopharmacology.* 2016; 233(23-24): 3921–3932. https://doi.org/10.1007/s00213-016-4424-5.

35. Sevinçer GM, Konuk N, Bozkurt S, et al. Food addiction and the outcome of bariatric surgery at 1-year: Prospective observational study. *Psychiatry Research.* 2016; 244 159–164. https://doi.org/10.1016/j.psychres.2016.07.022.

36. Murray SM, Tweardy S, Geliebter A, et al. A Longitudinal Preliminary Study of Addiction-Like Responses to Food and Alcohol Consumption Among Individuals

For peer review only

For peer review only - http://bmjopen.bmj.com/site/about/guidelines.xhtml

Undergoing Weight Loss Surgery. *Obesity Surgery.* 2019; 29(8): 2700–2703. https://doi.org/10.1007/s11695-019-03915-3.

37. Chao AM, Wadden TA, Tronieri JS, et al. Effects of addictive-like eating behaviors on weight loss with behavioral obesity treatment. *Journal of Behavioral Medicine.* 2019; 42(2): 246–255. https://doi.org/10.1007/s10865-018-9958-z.

38. Gordon EL, Merlo LJ, Durning PE, et al. Longitudinal Changes in Food Addiction Symptoms and Body Weight among Adults in a Behavioral Weight-Loss Program. *Nutrients.* 2020; 12(12): 3687. https://doi.org/10.3390/nu12123687.

39. Cassin S, Leung S, Hawa R, et al. Food Addiction Is Associated with Binge Eating and Psychiatric Distress among Post-Operative Bariatric Surgery Patients and May Improve in Response to Cognitive Behavioural Therapy. *Nutrients.* 2020; 12(10): 2905. https://doi.org/10.3390/nu12102905.

40. Unwin J, Delon C, Giæver H, et al. Low carbohydrate and psychoeducational programs show promise for the treatment of ultra-processed food addiction. *Frontiers in Psychiatry.* 2022; 13: 1005523. https://doi.org/10.3389/fpsyt.2022.1005523.

41. Carlos LO, Ramos MRZ, Wagner NRF, et al. Probiotic Supplementation attenuates binge eating and food addiction 1 year after roux-en-y gastric bypass: a randomized, double-blind, placebo controlled trial. *Arquivos brasileiros de cirurgia digestiva.* 35: e1659. https://doi.org/10.1590/0102-672020210002e1659.

42. Cassin SE, Sijercic I, and Montemarano V. Psychosocial Interventions for Food Addiction: A Systematic Review *Current Addiction Reports.* 2020; 7: 9–19. https://doi.org/10.1007/s40429-020-00295-y.

43. Hides L, Kavanagh DJ, Daglish M, et al. The Quik Fix study: a randomised controlled trial of brief interventions for young people with alcohol-related injuries and illnesses accessing emergency department and crisis support care. *BMC Emergency Medicine.* 2014; 14(19). https://doi.org/10.1186/1471-227X-14-19.

For peer review only

For peer review only - http://bmjopen.bmj.com/site/about/guidelines.xhtml

44. Hides L, Wilson H, Quinn C, et al. QuikFix: enhanced motivational interviewing interventions for youth substance use. *Advances in Dual Diagnosis.* 2016; 9(2/3): 53-65. https://doi.org/10.1108/ADD-03-2016-0008.

45. Murphy CM, Stojek MK, and MacKillop J. Interrelationships among impulsive personality traits, food addiction, and body mass index. *Appetite.* 2014; 73: 45–50. https://doi.org/10.1016/j.appet.2013.10.008.

46. Davis C. A narrative review of binge eating and addictive behaviors: shared associations with seasonality and personality factors. *Frontiers in Psychiatry.* 2013; 4: 183. https://doi.org/10.3389/fpsyt.2013.00183.

47. Brunault P, Ducluzeau PH, Courtois R, et al. Food Addiction is Associated with Higher Neuroticism, Lower Conscientiousness, Higher Impulsivity, but Lower Extraversion in Obese Patient Candidates for Bariatric Surgery. *Substance Use and Misuse.* 2018; 53(11): 1919–1923. https://doi.org/10.1080/10826084.2018.1433212.

48. Ouellette AS, Rodrigue C, Lemieux S, et al. An examination of the mechanisms and personality traits underlying food addiction among individuals with severe obesity awaiting bariatric surgery. *Eating and Weight Disorders.* 22(4): 633–640. https://doi.org/10.1007/s40519-017-0440-7.

49. Jiménez-Murcia S, Agüera Z, Paslakis G, et al. Food Addiction in Eating Disorders and Obesity: Analysis of Clusters and Implications for Treatment. *Nutrients.* 2019; 11(11): 2633. https://doi.org/10.3390/nu11112633.

50. Bunio LK, Battles JA, and Loverich TM. The nuances of emotion regulation difficulties and mindfulness in food addiction. *Addiction Research and Theory.* 2021; 29(1): 11-17. https://doi.org/10.1080/16066359.2020.1714038.

51. Miller W and Rollnick S. Motivational Interviewing. *3rd ed. New York, NY: Guilford Press.* 2013.

52. Leary M, Pursey KM, Verdejo-Garcia A, et al. Designing an online intervention for adults with addictive eating: A qualitative Integrated Knowledge Translation

For peer review only

For peer review only - http://bmjopen.bmj.com/site/about/guidelines.xhtml

approach. *BMJ Open.* 2022; 12(6): e060196. https://doi.org/10.1136/bmjopen-2021-060196.

53. Hoffmann T, Glasziou P, Boutron I, et al. Better reporting of interventions: template for intervention description and replication (TIDieR) checklist and guide. *BMJ.* 2014; 348: g1687. https://doi.org/10.1136/bmj.g1687.

54. Kelly JT, Allman-Farinelli M, Chen J, et al. Dietitians Australia position statement on telehealth. *Nutrition and Dietetics: the journal of the Dietitians Association of Australia.* 2020; 77(4): 406-415. https://doi.org/10.1111/1747-0080.12619.

55. Reynolds DJ Jr, Stiles WB, Bailer AJ, et al. Impact of exchanges and client-therapist alliance in online-text psychotherapy. *Cyberpsychology, Behavior and Social Networking.* 2013; 16(5): 370–377. https://doi.org/10.1089/cyber.2012.0195.

56. Chan A-W, Tetzlaff JM, Altman DG, et al. SPIRIT 2013 Statement: Defining standard protocol items for clinical trials. *Annals of Internal Medicine.* 2013; 158(3): 200-207. https://doi.org/10.7326/0003-4819-158-3-201302050-00583.

57. Schulz KF, Altman DG, Moher D, et al. CONSORT 2010 statement: updated guidelines for reporting parallel group randomised trials. *BMJ.* 2010; 340(c332). https://doi.org/10.1136/bmj.c332.

58. Purna Singh A, Vadakedath S, and Kandi V. Clinical Research: A Review of Study Designs, Hypotheses, Errors, Sampling Types, Ethics, and Informed Consent. *Cureus.* 2023; 5(1): e33374. https://doi.org/10.7759/cureus.33374.

59. Prnjak K, Mitchison D, Griffiths S, et al. Further development of the 12-item EDE-QS: identifying a cut-off for screening purposes. *BMC Psychiatry.* 2020; 20(1): 146. https://doi.org/10.1186/s12888-020-02565-5.

60. Australian Government Department of Health. Body mass index (BMI) and waist measurement. 2021 [cited 2022 8 March]; Available from: https://www.health.gov.au/health-topics/overweight-and-obesity/bmi-and-waist.

For peer review only

For peer review only - http://bmjopen.bmj.com/site/about/guidelines.xhtml

61. Centers for Disease Control and Prevention (CDC). Healthy Weight, Nutrition, and Physical Activity: Assessing Your Weight. 2021 [cited 2022 8 March]; Available from: https://www.cdc.gov/healthyweight/assessing/index.html.

62. Mond J, Hay P, Rodgers B, et al. Use of extreme weight control behaviors with and without binge eating in a community sample: implications for the classification of bulimic-type eating disorders*. International Journal of Eating Disorders.* 2006; 39(4): 294-302. https://doi.org/10.1002/eat.20265.

63. Mond JJ, Hay PJ, Rodgers B, et al. Correlates of the use of purging and non-purging methods of weight control in a community sample of women*. The Australian and New Zealand Journal of Psychiatry.* 2006; 40(2): 136-142. https://doi.org/10.1080/j.1440-1614.2006.01760.x.

64. WHO ASSIST Working Group. The Alcohol, Smoking and Substance Involvement Screening Test (ASSIST): development, reliability and feasibility*. Addiction.* 2002; 97(9): 1183-1194. https://doi.org/10.1046/j.1360-0443.2002.00185.x.

65. Spitzer RL, Kroenke K, Williams JBW, et al. A Brief Measure for Assessing Generalized Anxiety Disorder (The GAD-7)*. Archives of Internal Medicine.* 2006; 166(10): 1092–1097 https://doi.org/10.1001/archinte.166.10.1092.

66. Ingram PB 4th, Clarke E, and Lichtenberg JW. Confirmatory Factor Analysis of the Perceived Stress Scale-4 in a Community Sample*. Stress Health.* 2016; 32(2): 173–176. https://doi.org/10.1002/smi.2592.

67. Fairburn C, Cooper Z, and O'Connor M, *Eating disorders examination (16.0D)* in *In: Cognitive behavior therapy and eating disorders*, Fairburn C (Ed.), Editor. 2008, Guilford Press: New York.

68. Kroenke K, Strine TW, Spitzer RL, et al. The PHQ-8 as a measure of current depression in the general population*. Journal of affective disorders.* 2009; 114(1-3): 163-173. https://doi.org/10.1016/j.jad.2008.06.026.

For peer review only - http://bmjopen.bmj.com/site/about/guidelines.xhtml

69. Collins CE, Boggess MM, Watson JF, et al. Reproducibility and comparative validity of a food frequency questionnaire for Australian adults. *Clinical Nutrition.* 2014; 33(5): 906-914. https://doi.org/10.1016/j.clnu.2013.09.015.

70. Ashton L, Williams R, Wood L, et al. Comparison of Australian Recommended Food Score (ARFS) and Plasma Carotenoid Concentrations: A Validation Study in Adults. *Nutrients.* 2017; 9(8): 888. http://doi.org/10.3390/nu9080888.

71. Woicik PA, Stewart SH, Pihl RO, et al. The Substance Use Risk Profile Scale: a scale measuring traits linked to reinforcement-specific substance use profiles. *Addictive Behaviors.* 2009; 34(12): 1042-1055. https://doi.org/10.1016/j.addbeh.2009.07.001.

72. Gormally J, Black S, Daston S, et al. The assessment of binge eating severity among obese persons. *Addictive Behaviors.* 1982; 7(1): 47-55. https://doi.org/10.1016/0306-4603(82)90024-7.

73. Heriseanu AI, Hay P, and Touyz S. The short inventory of grazing (SIG): development and validation of a new brief measure of a common eating behaviour with a compulsive dimension. *Journal of Eating Disorders.* 2019; 7(4). https://doi.org/10.1186/s40337-019-0234-6.

74. Vainik U, Han C, Epel ES, et al. Rapidly assessing reward-related eating: The RED-X5. *Obesity.* 2019; 27(2): 325–331. https://doi.org/10.1002/oby.22374.

75. Hibbard JH, Stockard J, Mahoney ER, et al. Development of the Patient Activation Measure (PAM): conceptualizing and measuring activation in patients and consumers. *Health Services Research.* 2004; 39(4 Pt 1): 1005–1026. https://doi.org/10.1111/j.1475-6773.2004.00269.x.

76. Rabin R and de Charro F. EQ-5D: a measure of health status from the EuroQol Group. *Annals of Medicine.* 2001; 33(5): 337-343. https://doi.org/10.3109/07853890109002087.

For peer review only

For peer review only - http://bmjopen.bmj.com/site/about/guidelines.xhtml

77. Australian Institute of Health and Welfare (AIHW). The Active Australia Survey: a guide and manual for implementation, analysis and reporting. 2003; Available from: https://www.aihw.gov.au/reports/physical-activity/active-australia-survey/summary.

78. Buysse DJ, Reynolds CF 3rd, Monk TH, et al. The Pittsburgh Sleep Quality Index: a new instrument for psychiatric practice and research. *Psychiatry Research.* 1989; 28(2): 193–213. https://doi.org/10.1016/0165-1781(89)90047-4.

79. Beecham J and Knapp M, *Costing psychiatric interventions*, in *Measuring Mental Health Needs (2nd edition)*, Thornicroft G, Editor. 2001, Gaskell: London. p. 200-224.

80. Pursey KM, Collins R, Skinner J, et al. Characteristics of individuals seeking addictive eating treatment. *Eating and Weight Disorders.* 2021; 26(8): 2779-2786. https://doi.org/10.1007/s40519-021-01147-y.

81. Collins R, Haracz K, Leary M, et al. No control and overwhelming cravings: Australian adults' perspectives on the experience of food addiction. *Appetite.* 2021; 59(105054). https://doi.org/10.1016/j.appet.2020.105054.

82. Pedram P, Wadden D, Amini P, et al. Food addiction: its prevalence and significant association with obesity in the general population. *PLOS One.* 2013; 8(9): e74832. https://doi.org/10.1371/journal.pone.0074832.

83. Lemeshow AR, Gearhardt AN, Genkinger JM, et al. Assessing the psychometric properties of two food addiction scales *Eating Behaviors.* 2016; 23: 110–114. https://doi.org/10.1016/j.eatbeh.2016.08.005.

84. National Health and Medical Research Council (NHMRC). Australian Guide to Healthy Eating. 2017; Available from: https://www.eatforhealth.gov.au/guidelines/australian-guide-healthy-eating.

85. Food Standards Australia New Zealand (FSANZ). AUSNUT 2011–13–Australian Food Composition Database. 2014 [cited 2021 31 May]; Available from: http://foodstandards.gov.au/.

86. National Health and Medical Research Council. Australian Dietary Guidelines. Canberra. 2013; Available from: www.nhmrc.gov.au/guidelines-publications/n55.

For peer review only

For peer review only - http://bmjopen.bmj.com/site/about/guidelines.xhtml

87. Personal Social Services Research Unit (PSSRU). 2022 [cited 2022 14 April]; Available from: https://www.pssru.ac.uk/csri/client-service-receipt-inventory/.

88. Mauskopf JA, Paul JE, Grant DM, et al. The role of cost-consequence analysis in healthcare decision-making. *Pharmacoeconomics.* 1998; 13(3): 277-288. https://doi.org/10.2165/00019053-199813030-00002.

89. Hunter R and Shearer J. Cost-consequences analysis - an underused method of economic evaluation. [cited 2022 8 March]; Available from: https://www.rds-london.nihr.ac.uk/wpcms/wp-content/uploads/2018/09/Cost-consequences-analysis-an-underused-method.pdf.

90. Hibbard JH, Mahoney ER, Stockard J, et al. Development and testing of a short form of the patient activation measure. *Health Services Research.* 2005; 40(6 Pt 1): 1918-1930. https://doi.org/10.1111/j.1475-6773.2005.00438.x.

91. Greene J and Hibbard JH. Why does patient activation matter? An examination of the relationships between patient activation and health-related outcomes. *Journal of General Internal Medicine.* 2012; 27(5): 520–526. https://doi.org/10.1007/s11606-011-1931-2.

92. Insigina Health, *Patient activation measure (PAM) 13 TM Licence Materials copyright*. 2011, LLC: Insigna Health.

93. Remmers C, Hibbard J, Mosen DM, et al. Is patient activation associated with future health outcomes and healthcare utilization among patients with diabetes? *The Journal of Ambulatory Care Management.* 2009; 32(4): 320-327. https://doi.org/10.1097/JAC.0b013e3181ba6e77.

94. Australian Bureau of Statistics (ABS). 2033.0.55.001 - Socio-Economic Indexes for Areas (SEIFA). 2016 [cited 2021 14 September]; Available from: www.abs.gov.au.

95. World Health Organization (WHO). Body mass index - BMI. c2018 [cited 2021 September 1]; Available from: http://www.euro.who.int/en/health-topics/disease-prevention/nutrition/a-healthy-lifestyle/body-mass-index-bmi.

For peer review only

96. Harris PA, Taylor R, Minor BL, et al. REDCap Consortium. The REDCap consortium: Building an international community of software platform partners. *Journal of Biomedical Informatics.* 2019; 95(103208). https://doi.org/10.1016/j.jbi.2019.103208.

97. Harris PA, Taylor R, Thielke R, et al. Research electronic data capture (REDCap) – A metadata-driven methodology and workflow process for providing translational research informatics support. *Journal of Biomedical Informatics.* 2009; 42(2): 377-381. https://doi.org/10.1016/j.jbi.2008.08.010.

For peer review only

For peer review only - http://bmjopen.bmj.com/site/about/guidelines.xhtml

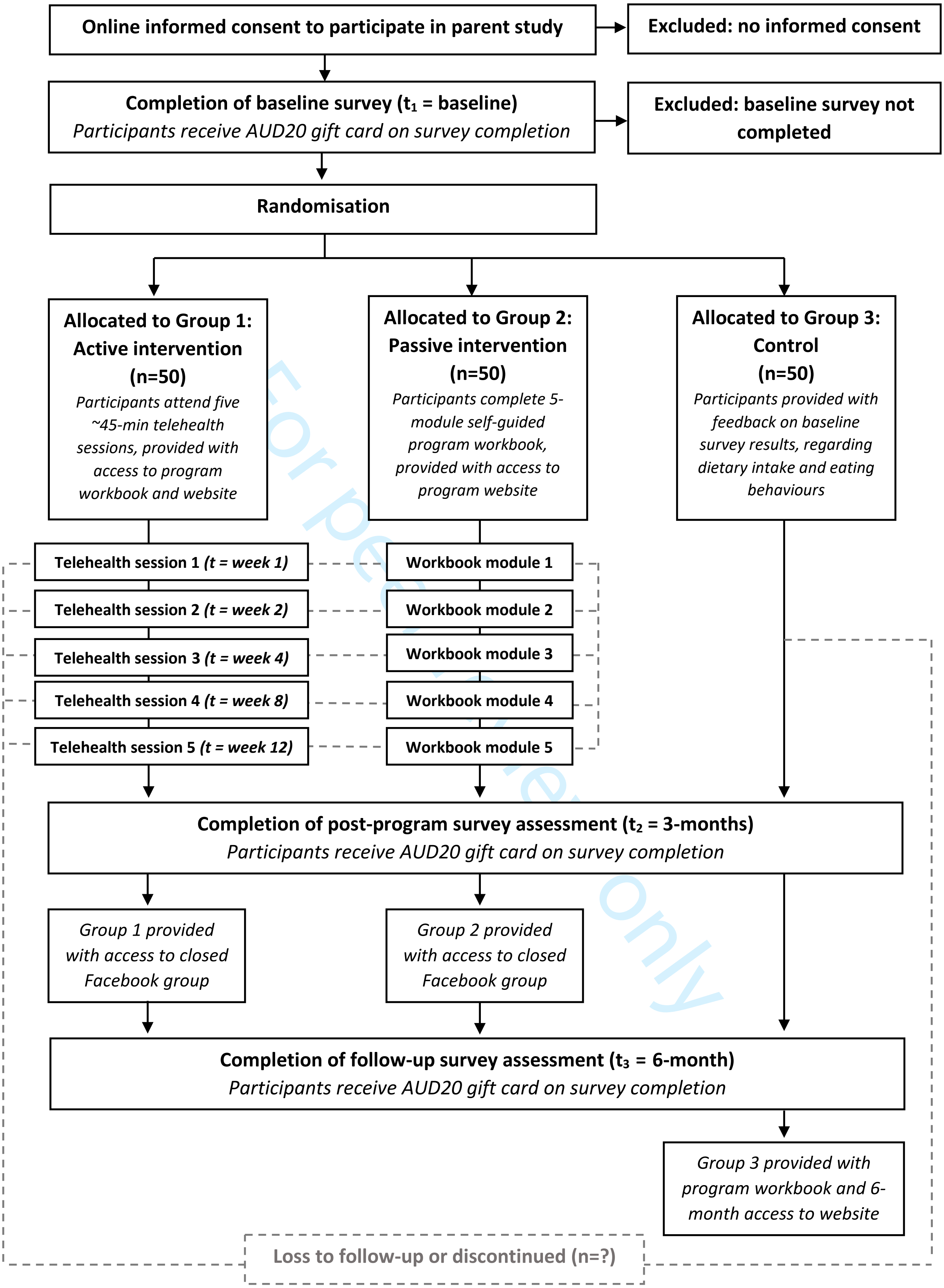

For peer review only

WELCOME BACK!As you have been found eligible to participate in the personality based intervention for addictive eating, it is important that you carefullly read the following information and give consent at the bottom if you wish to continue to the baseline surveys.

I have read the previous Participant Information Statement and give my consent to participate in this study?

◯ Yes
◯ No

I understand that it is possible that some questionnaires may identify potential health issues that may require follow-up with my GP. I give consent for a copy of the relevant results to be sent to my local doctor/GP or other Health Professional

◯ Yes
◯ No

Please provide Health Professional details (e.g Name, Phone number)

____

I agree to participate in the Personality based intervention for Addictive Eating Behaviours study and give my consent freely.

I understand that the project will be conducted as described in the previous Participant Information Statement, a copy of which I have had the opportunity to download.

I understand I can withdraw from the project at any time and do not have to give any reason for withdrawing.
I am aware I have an equal chance of being allocated into one of three intervention groups. If allocated to:

• Group 1, I consent to completing online questionnaires at the beginning of the study and after 3 and 6 months, and to participating in five telehealth/phone consultations of 30-45mins with an Accredited Practising Dietitian.

• Group 2, I consent to completing online questionnaires at the beginning of the study and after 3 and 6 months, and to complete the self-guided workbook and access the study website.

• Group 3, I consent to completing online questionnaires at the beginning of the study and after 3 and 6 months, and to follow my usual dietary intake for the study duration. I understand that after 6 months I will have access to complete the self-guided workbook and access to the study website.

If allocated to group 1, I consent for my five sessions with the dietitian to be recorded for quality and training purposes.

◯ Yes
◯ No

Please provide your First Name:

____

Please provide your Last Name:

____

Please sign:

____

Do you wish to continue to the Baseline Surveys?

◯ Yes
◯ No

For peer review only

Title: Examining the efficacy of a telehealth intervention targeting addictive eating in Australian adults (the TRACE program): a randomised controlled trial protocol

Reference to where data collection forms can be found

Survey	Assessment tool	Reference	Available from
**AAS**	**Active Australia Survey**	Australian Institute of Health and Welfare (AIHW) 2003. The Active Australia Survey: a guide and manual for implementation, analysis and reporting. Canberra: AIHW.	https://www.aihw.gov.au/reports/physical-activity/active-australia-survey/summary
**AES**	**Australian Eating Survey**	Ashton L, Williams R, Wood L, Schumacher T, Burrows T, Rollo M, et al. Comparison of Australian Recommended Food Score (ARFS) and Plasma Carotenoid Concentrations: A Validation Study in Adults. Nutrients. 2017;9(8):888. http://doi.org/10.3390/nu9080888<br><br>Collins CE, Boggess MM, Watson JF, Guest M, Duncanson K, Pezdirc K, et al. Reproducibility and comparative validity of a food frequency questionnaire for Australian adults. Clinical Nutrition. 2014;33(5):906-14. doi: 10.1016/j.clnu.2013.09.015	https://australianeatingsurvey.com.au/
**ASSIST**	**Alcohol, Smoking and Substance Involvement Screening Test - Version 3.0**	WHO ASSIST Working Group (2002) The Alcohol, Smoking and Substance Involvement Screening Test (ASSIST): development, reliability and feasibility. Addiction, 97:1183-1194. doi: 10.1046/j.1360-0443.2002.00185.x	https://www.who.int/publications/i/item/978924159938-2
**BES**	**Binge Eating Scale**	Gormally J, Black S, Daston S, Rardin D. The assessment of binge eating severity among obese persons. Addictive Behaviors. 1982;7(1):47-55. https://doi.org/10.1016/0306-4603(82)90024-7	Available in the publication
**CSRI**	**Consumer Services Receipt Inventory**	Beecham J and Knapp M. (2001) Costing psychiatric interventions, in G. Thornicroft (ed.) Measuring Mental Health Needs, Gaskell, 2nd edition, 200-224.	https://www.pssru.ac.uk/csri/what-is-the-csri/
**EDE-Q 6.0**	**Eating Disorder**	Fairburn C, Cooper Z, O'Connor M. Eating disorders examination (16.0D) In: Fairburn C (Ed.), editor. In:	https://nedc.com.au/assets/Medicare-related-forms/Eating-Disorder-Examination-Questionaire-Smart-PDF.pdf

For peer review only

	**Examination Questionnaire 6.0**	Cognitive behavior therapy and eating disorders. New York: Guilford Press; 2008.	
**EDE-QS**	**Eating Disorder Examination Questionnaire Short Form**	Prnjak K, Mitchison D, Griffiths S, Mond J, Gideon N, Serpell L, Hay P. Further development of the 12-item EDE-QS: identifying a cut-off for screening purposes. BMC Psychiatry. 2020;20:146. https://doi.org/10.1186/s12888-020-02565-5	Available as Supporting Information accompanying the publication
**EQ5D-5L**	**EQ5D-5L**	Brazier J, Ratcliffe J, Tsuchiya A, Salomon J. Measuring and Valuing Health Benefits for Economic Evaluation. 2nd ed. Oxford: Oxford University Press; 2016. doi: 10.1093/med/9780198725923.001.0001.	https://aci.health.nsw.gov.au/__data/assets/pdf_file/0003/632847/EuroQol-5-Dimension.pdf
**GAD-7**	**Generalized Anxiety Disorder 7**	Spitzer RL, Kroenke K, Williams JBW, Löwe B. A Brief Measure for Assessing Generalized Anxiety Disorder (The GAD-7). Archives of Internal Medicine. 2006;166(10):1092–7 https://doi.org/10.1001/archinte.166.10.1092	https://adaa.org/sites/default/files/GAD-7_Anxiety-updated_0.pdf
**PAM-13**	**Patient Activation Measure 13-item**	Hibbard JH, Stockard J, Mahoney ER, Tusler M. (2004). Development of the Patient Activation Measure (PAM): conceptualizing and measuring activation in patients and consumers. Health services research. 2004;39(4 Pt 1): 1005–1026. https://doi.org/10.1111/j.1475-6773.2004.00269.x	https://www.insigniahealth.com/products/pam
**PQH-8**	**Patient Health Questionnaire**	Kroenke K, et al., The PHQ-8 as a measure of current depression in the general population. Journal of affective disorders, 2009. 114(1-3): p. 163-173 https://doi.org/10.1016/j.jad.2008.06.026	https://www.psychologywizard.net/uploads/2/6/6/4/26640833/kroenke_phq8.pdf
**PSQI**	**Pittsburgh Sleep Quality Index**	Buysse DJ, Reynolds CF 3rd, Monk TH, Berman SR, Kupfer DJ. The Pittsburgh Sleep Quality Index: a new instrument for psychiatric practice and research. Psychiatry Research. 1989;28(2): 193–213. https://doi.org/10.1016/0165-1781(89)90047-4	https://www.med.upenn.edu/cbti/assets/user-content/documents/Pittsburgh%20Sleep%20Quality%20Index%20(PSQI).pdf
**PSS-4**	**Perceived Stress Scale**	Ingram PB 4th, Clarke E, Lichtenberg JW. Confirmatory Factor Analysis of the Perceived Stress Scale-4 in a Community Sample. Stress Health. 2016; 32(2): 173–176. https://doi.org/10.1002/smi.2592	https://scholar.harvard.edu/files/bettina.hoeppner/files/pss-4.pdf

For peer review only

**REDX-5**	**Reward-Based Eating Drive Scale**	Vainik U, Han C, Epel ES, Dagher A, Mason AE. Rapid assessment of reward-related eating: The RED-X5. Obesity. 2019;27(2):325–31. doi:10.1002/oby.22374	https://www.ncbi.nlm.nih.gov/pmc/articles/PMC6352904/
**SURPS**	**Substance Use Risk Profile Scale**	Woicik PA, Stewart SH, Pihl RO, Conrod PJ. The Substance Use Risk Profile Scale: a scale measuring traits linked to reinforcement-specific substance use profiles. Addictive Behaviors. 2009;34(12): 1042-55. doi: 10.1016/j.addbeh.2009.07.001	Available on request from the corresponding author: Woicik can be contacted at Neuropsychoimaging Group, Brookhaven National Laboratory, Medical Department, Building 490, Upton, New York, 11973, United States. Tel.: +1 631 344 4472. Conrod, NIHR Biomedical Research Centre, Section of Addiction, Department of Psychological Medicine and Psychiatry, King's College London, 4 Windsor Walk, Denmark Hill, London, SE5 8BB, United Kingdom. Tel.: +44 207 848 0836; fax: +44 207 701 8584.p.conrod@iop.kcl.ac.uk
**YFAS 2.0**	**Yale Food Addiction Scale 2.0**	Gearhardt AN, Corbin WR, Brownell KD. Development of the Yale Food Addiction Scale Version 2.0. Psychology of Addictive Behaviors. 2016;30(1):113-21. doi: 10.1037/adb0000136	https://sites.lsa.umich.edu/fastlab/yale-food-addiction-scale/

For peer review only

SPIRIT — STANDARD PROTOCOL ITEMS: RECOMMENDATIONS FOR INTERVENTIONAL TRIALS

SPIRIT 2013 Checklist: Recommended items to address in a clinical trial protocol and related documents*

Section/item	Item No	Description	Addressed on page number
**Administrative information**			
Title	1	Descriptive title identifying the study design, population, interventions, and, if applicable, trial acronym	2
Trial registration	2a	Trial identifier and registry name. If not yet registered, name of intended registry	3, 8
	2b	All items from the World Health Organization Trial Registration Data Set	NA
Protocol version	3	Date and version identifier	-
Funding	4	Sources and types of financial, material, and other support	32
Roles and responsibilities	5a	Names, affiliations, and roles of protocol contributors	1, 31
	5b	Name and contact information for the trial sponsor	1
	5c	Role of study sponsor and funders, if any, in study design; collection, management, analysis, and interpretation of data; writing of the report; and the decision to submit the report for publication, including whether they will have ultimate authority over any of these activities	30
	5d	Composition, roles, and responsibilities of the coordinating centre, steering committee, endpoint adjudication committee, data management team, and other individuals or groups overseeing the trial, if applicable (see Item 21a for data monitoring committee)	NA

For peer review only

For peer review only - http://bmjopen.bmj.com/site/about/guidelines.xhtml

Introduction

Background and rationale	6a	Description of research question and justification for undertaking the trial, including summary of relevant studies (published and unpublished) examining benefits and harms for each intervention	4 – 8
	6b	Explanation for choice of comparators	14, 15
Objectives	7	Specific objectives or hypotheses	8
Trial design	8	Description of trial design including type of trial (eg, parallel group, crossover, factorial, single group), allocation ratio, and framework (eg, superiority, equivalence, noninferiority, exploratory)	8

Methods: Participants, interventions, and outcomes

Study setting	9	Description of study settings (eg, community clinic, academic hospital) and list of countries where data will be collected. Reference to where list of study sites can be obtained	8
Eligibility criteria	10	Inclusion and exclusion criteria for participants. If applicable, eligibility criteria for study centres and individuals who will perform the interventions (eg, surgeons, psychotherapists)	9, 10
Interventions	11a	Interventions for each group with sufficient detail to allow replication, including how and when they will be administered	10 - 20
	11b	Criteria for discontinuing or modifying allocated interventions for a given trial participant (eg, drug dose change in response to harms, participant request, or improving/worsening disease)	8
	11c	Strategies to improve adherence to intervention protocols, and any procedures for monitoring adherence (eg, drug tablet return, laboratory tests)	19, 20
	11d	Relevant concomitant care and interventions that are permitted or prohibited during the trial	NA
Outcomes	12	Primary, secondary, and other outcomes, including the specific measurement variable (eg, systolic blood pressure), analysis metric (eg, change from baseline, final value, time to event), method of aggregation (eg, median, proportion), and time point for each outcome. Explanation of the clinical relevance of chosen efficacy and harm outcomes is strongly recommended	20 - 27
Participant timeline	13	Time schedule of enrolment, interventions (including any run-ins and washouts), assessments, and visits for participants. A schematic diagram is highly recommended (see Figure)	11 (Table 1)

For peer review only - http://bmjopen.bmj.com/site/about/guidelines.xhtml

Sample size	14	Estimated number of participants needed to achieve study objectives and how it was determined, including clinical and statistical assumptions supporting any sample size calculations	27
Recruitment	15	Strategies for achieving adequate participant enrolment to reach target sample size	9
**Methods: Assignment of interventions (for controlled trials)**			
Allocation:			
Sequence generation	16a	Method of generating the allocation sequence (eg, computer-generated random numbers), and list of any factors for stratification. To reduce predictability of a random sequence, details of any planned restriction (eg, blocking) should be provided in a separate document that is unavailable to those who enrol participants or assign interventions	13, 14
Allocation concealment mechanism	16b	Mechanism of implementing the allocation sequence (eg, central telephone; sequentially numbered, opaque, sealed envelopes), describing any steps to conceal the sequence until interventions are assigned	13, 14
Implementation	16c	Who will generate the allocation sequence, who will enrol participants, and who will assign participants to interventions	13, 14
Blinding (masking)	17a	Who will be blinded after assignment to interventions (eg, trial participants, care providers, outcome assessors, data analysts), and how	13, 14
	17b	If blinded, circumstances under which unblinding is permissible, and procedure for revealing a participant's allocated intervention during the trial	NA
**Methods: Data collection, management, and analysis**			
Data collection methods	18a	Plans for assessment and collection of outcome, baseline, and other trial data, including any related processes to promote data quality (eg, duplicate measurements, training of assessors) and a description of study instruments (eg, questionnaires, laboratory tests) along with their reliability and validity, if known. Reference to where data collection forms can be found, if not in the protocol	19, 20, 21 - 29, suppl
	18b	Plans to promote participant retention and complete follow-up, including list of any outcome data to be collected for participants who discontinue or deviate from intervention protocols	13

Data management	19	Plans for data entry, coding, security, and storage, including any related processes to promote data quality (eg, double data entry; range checks for data values). Reference to where details of data management procedures can be found, if not in the protocol	28, 29
Statistical methods	20a	Statistical methods for analysing primary and secondary outcomes. Reference to where other details of the statistical analysis plan can be found, if not in the protocol	27, 28
	20b	Methods for any additional analyses (eg, subgroup and adjusted analyses)	27, 28
	20c	Definition of analysis population relating to protocol non-adherence (eg, as randomised analysis), and any statistical methods to handle missing data (eg, multiple imputation)	28
**Methods: Monitoring**			
Data monitoring	21a	Composition of data monitoring committee (DMC); summary of its role and reporting structure; statement of whether it is independent from the sponsor and competing interests; and reference to where further details about its charter can be found, if not in the protocol. Alternatively, an explanation of why a DMC is not needed	29
	21b	Description of any interim analyses and stopping guidelines, including who will have access to these interim results and make the final decision to terminate the trial	NA
Harms	22	Plans for collecting, assessing, reporting, and managing solicited and spontaneously reported adverse events and other unintended effects of trial interventions or trial conduct	29
Auditing	23	Frequency and procedures for auditing trial conduct, if any, and whether the process will be independent from investigators and the sponsor	29
**Ethics and dissemination**			
Research ethics approval	24	Plans for seeking research ethics committee/institutional review board (REC/IRB) approval	8, 31
Protocol amendments	25	Plans for communicating important protocol modifications (eg, changes to eligibility criteria, outcomes, analyses) to relevant parties (eg, investigators, REC/IRBs, trial participants, trial registries, journals, regulators)	NA

Consent or assent	26a	Who will obtain informed consent or assent from potential trial participants or authorised surrogates, and how (see Item 32)	9, 10, 12
	26b	Additional consent provisions for collection and use of participant data and biological specimens in ancillary studies, if applicable	NA
Confidentiality	27	How personal information about potential and enrolled participants will be collected, shared, and maintained in order to protect confidentiality before, during, and after the trial	28, 29
Declaration of interests	28	Financial and other competing interests for principal investigators for the overall trial and each study site	33
Access to data	29	Statement of who will have access to the final trial dataset, and disclosure of contractual agreements that limit such access for investigators	28, 29
Ancillary and post-trial care	30	Provisions, if any, for ancillary and post-trial care, and for compensation to those who suffer harm from trial participation	NA
Dissemination policy	31a	Plans for investigators and sponsor to communicate trial results to participants, healthcare professionals, the public, and other relevant groups (eg, via publication, reporting in results databases, or other data sharing arrangements), including any publication restrictions	31
	31b	Authorship eligibility guidelines and any intended use of professional writers	NA
	31c	Plans, if any, for granting public access to the full protocol, participant-level dataset, and statistical code	31
**Appendices**			
Informed consent materials	32	Model consent form and other related documentation given to participants and authorised surrogates	Suppl.
Biological specimens	33	Plans for collection, laboratory evaluation, and storage of biological specimens for genetic or molecular analysis in the current trial and for future use in ancillary studies, if applicable	NA

*It is strongly recommended that this checklist be read in conjunction with the SPIRIT 2013 Explanation & Elaboration for important clarification on the items. Amendments to the protocol should be tracked and dated. The SPIRIT checklist is copyrighted by the SPIRIT Group under the Creative Commons "Attribution-NonCommercial-NoDerivs 3.0 Unported" license.

BMJ Open

Examining the efficacy of a telehealth intervention targeting addictive eating in Australian adults (the TRACE program): a randomised controlled trial protocol

Journal:	*BMJ Open*
Manuscript ID	bmjopen-2022-064151.R2
Article Type:	Protocol
Date Submitted by the Author:	04-May-2023

Complete List of Authors:	Skinner, Janelle; The University of Newcastle, School of Health<br>Whatnall, Megan; The University of Newcastle, School of Health<br>Leary, Mark; The University of Newcastle,<br>Collins, Rebecca; The University of Newcastle, School of Health<br>Pursey, Kirrilly; The University of Newcastle, School of Health<br>Verdejo-García, Antonio; Monash University<br>Hay, Phillipa ; Western Sydney University<br>Baker, Amanda; The University of Newcastle,<br>Hides, Leanne; University of Queensland,<br>Paxton, Susan; La Trobe University<br>Wood, Lisa; The University of Newcastle, Respiratory Medicine<br>Colyvas, Kim; The University of Newcastle, School of Mathematical and Physical Sciences<br>Collins, Clare; The University of Newcastle<br>Burrows, Tracy; The University of Newcastle; The University of Newcastle Hunter Medical Research Institute

**Primary Subject Heading**:	Addiction
Secondary Subject Heading:	Mental health
Keywords:	NUTRITION & DIETETICS, PUBLIC HEALTH, Eating disorders < PSYCHIATRY

SCHOLARONE™
Manuscripts

Article type: Protocol

Title: Examining the efficacy of a telehealth intervention targeting addictive eating in Australian adults (the TRACE program): a randomised controlled trial protocol

Authors: Janelle A Skinner[1,2], Megan Whatnall[1,2], Mark Leary[1,2], Rebecca A Collins[1,2], Kirrilly M Pursey[1,2], Antonio Verdejo-Garcia[3], Phillipa J Hay[4], Amanda L Baker[2,5], Leanne Hides[6], Susan J Paxton[7], Lisa G Wood[2,8], Kim Colyvas[1], Clare E Collins[1,2], Tracy L Burrows[1,2*]

Institutions:

[1]School of Health Sciences, College of Health, Medicine and Wellbeing, University of Newcastle, Callaghan NSW 2308, Australia

[2]Hunter Medical Research Institute, University of Newcastle, New Lambton Heights, NSW, 2305, Australia

[3]School of Psychological Sciences and Turner Institute for Brain and Mental Health, Monash University, Clayton, VIC 3800, Australia

[4]Translational Health Research Institute, Western Sydney University, Campbelltown, NSW, 2751, Australia

[5]School of Medicine and Public Health, College of Health, Medicine and Wellbeing, University of Newcastle, Callaghan, NSW 2308, Australia

[6]School of Psychology, University of Queensland, St Lucia, QLD, 4072, Australia

[7]Department of Psychology and Counselling, Latrobe University, Melbourne, VIC, 3086

[8]School of Biomedical Sciences and Pharmacy, College of Health, Medicine and Wellbeing, University of Newcastle, Callaghan NSW 2308, Australia

***Corresponding Author:** Professor Tracy Burrows. Mailing address: School of Health Sciences, University Drive, University of Newcastle, Callaghan, NSW, Australia, 2308. Ph: +610249215514. Email: tracy.burrows@newcastle.edu.au

Word count: 7843

Key words: addictive eating, food addiction, Yale Food Addiction Scale

For peer review only

For peer review only - http://bmjopen.bmj.com/site/about/guidelines.xhtml

ARTICLE SUMMARY

Title: Examining the efficacy of a telehealth intervention targeting addictive eating in Australian adults (the TRACE program): a randomised controlled trial protocol

Abstract

Introduction *A*pproximately 15-20% of the adult population self-report symptoms of addictive eating. There are currently limited options for management. Motivational interviewing-based interventions, containing personalised coping skills training, have been found to be effective for behaviour change in addictive disorders (e.g. alcohol). This project builds upon foundations of an addictive eating feasibility study previously conducted, and co-design process involving consumers. The primary aim of this study is to examine the efficacy of a telehealth intervention targeting addictive eating symptoms in Australian adults compared to passive intervention and control groups. **Methods and analysis** This three-arm randomised controlled trial will recruit participants, 18-85 years, endorsing ≥3 symptoms on the Yale Food Addiction Scale 2.0, with BMI >18.5kg/m^2. Addictive eating symptoms are assessed at baseline (pre-intervention), 3-months (post-intervention) and 6-months. Other outcomes include dietary intake and quality, depression, anxiety, stress, quality of life, physical activity, and sleep hygiene. Using a multicomponent clinician-led approach, the active intervention consists of five telehealth sessions (15-45min each) delivered by a dietitian over 3-months. The intervention uses personalised feedback, skill-building exercises, reflective activities, and goal setting. Participants are provided with a workbook and website access. The passive intervention group receive the intervention via a self-guided approach with access to the workbook and website (no telehealth). The control group receive personalised written dietary feedback at baseline and participants advised to follow their usual dietary pattern for 6-months. The control group will be offered the passive intervention after 6-months. The primary endpoint is YFAS symptom scores at 3-months. A cost consequence analysis will determine intervention costs alongside mean change outcomes. **Ethics and dissemination** Human Research Ethics Committee of

For peer review only - http://bmjopen.bmj.com/site/about/guidelines.xhtml

University of Newcastle Australia provided approval: H-2021-0100. Findings will be disseminated via publication in peer-reviewed journals, conference presentations, community presentations and student theses. Trial registration: Australia New Zealand Clinical Trial Registry (ANZCTR) ACTRN12621001079831.

Strengths and Limitations of this study

- Targeting addictive risk factors through personalised tailoring of coping strategies and use of motivational interviewing for management of symptoms of addictive eating
- Co-design approach taken, with both consumers and multidisciplinary health professionals, to inform program development
- Detailed assessment of eating behaviours, mental health and lifestyle factors with personalised feedback provided to participants during the telehealth intervention
- Fidelity outcomes will be assessed, and cost consequence analysis conducted regarding implementation
- Limitations include participants being excluded with severe mental illnesses or complex health conditions

INTRODUCTION

For peer review only - http://bmjopen.bmj.com/site/about/guidelines.xhtml

Research in addictive eating has increased rapidly in recent years. Addictive eating, theorised as being on the severe end of a spectrum of overeating,[1] is a phenotype of eating behaviour marked by the chronic excessive and dysregulated consumption of food.[2, 3] Addictive eating, not categorised as a distinct disorder in the Diagnostic and Statistical Manual of Mental Disorders (DSM)[4] or the International Classification of Disease[5] systems, is most commonly assessed using the Yale Food Addiction Scale (YFAS).[6] The YFAS adapts the DSM criteria for 'substance-related and addictive disorders' to specific foods.[4] Developed in 2009[2] and revised in 2016 (YFAS 2.0)[6] according to the DSM-5 criteria, this psychometric tool assesses the presence of 11 symptoms of addictive eating. Symptoms include craving, loss of control, tolerance and withdrawal associated with eating behaviours, the repeated unsuccessful attempts to reduce the consumption of specific foods and maintenance of these behaviours despite adverse physical/emotional/social/interpersonal consequences.[6] The YFAS 2.0 provides two scoring options: a continuous symptom score, reflecting the number of endorsed addiction-like symptoms; and a dichotomous diagnosis of 'food addiction'.[6] Using this self-report survey, approximately 15-20% of the adult population endorse ≥ three YFAS symptoms for addictive eating. [7-9]

Higher prevalence rates of addictive eating have been reported in individuals with higher Body Mass Indexes (BMI) classified as overweight or obese compared to lower BMIs.[8, 10] Although addictive eating is not exclusive to those with higher weight status.[11] It has been suggested that addictive eating in those with underweight may be related to dietary restriction practices (e.g., consuming more than intended that breaches self-imposed dietary rules, intense craving resulting from extreme dieting practices).[11, 12] Irrespective of weight status, results from recent research indicate that individuals with addictive eating have significantly lower diet quality and higher intakes of highly processed foods.[9, 13, 14] Poor diet is a significant contributor to early death globally[15] and addressing addictive eating may contribute to the prevention or management of adverse health outcomes.

For peer review only

Addictive eating is a complex issue often overlapping with other health conditions, and likely transdiagnostic.[16, 17] There is evidence that addictive eating commonly co-occurs with mental health co-morbidities, particularly depression and anxiety, as well as overlapping with eating disorders, specifically binge eating disorder (BED).[7, 14] Approximately 50% of individuals with BED meet criteria for 'food addiction' according to the YFAS.[7] The present state of the literature demonstrates there is considerable overlap between BED and addictive eating.[18, 19] Commonalities include the loss of control over consumption, continued overuse despite negative consequences, and repeated failed attempts to reduce consumption.[19] At this time, it is unclear if addictive eating will emerge as a severe subtype of BED or be regarded as a distinct form of an addiction disorder. This distinction will be important to allow for targeted treatment and prevention strategies in susceptible individuals.

Current treatment options for addictive eating largely stem from online self-help groups such as Food Addicts Anonymous[20] and Overeaters Anonymous.[21] Originating in the United States, they now have 10 000+ members worldwide and have been in existence for many years, demonstrating a need for services.[22] A 2021 systematic review[23] found there is limited evidence supporting implementation of feasible and effective dietary interventions run by clinicians, for the management of addictive eating.[24] Of the nine studies reviewed, five studies that included lifestyle modification,[25] medication,[26, 27] or bariatric surgery[28, 29] were found to improve symptoms of addictive eating.[23] Since publication of this review, a further four intervention studies (a behavioural weight loss program, [30] a brief telephone-based cognitive behavioural therapy intervention,[31] a low carbohydrate dietary program,[32] and a probiotic supplement placebo-controlled trial[33]) have been trialled with improvement in YFAS addictive eating symptomatology immediately following the intervention. To date, most studies have been limited in sample size and therefore not been powered to detect a change in addictive eating symptoms.[23] Given the limited number of treatment options for addictive eating, there is a clear need for services, and development and testing of

For peer review only - http://bmjopen.bmj.com/site/about/guidelines.xhtml

interventions. It has been suggested that interventions based on substance use addiction models may be effective at facilitating changes in eating behaviour.[34]

Motivational interviewing (MI) based interventions for addictive disorders, such as alcohol use, in combination with coping skills training for traits associated with risk of addictive behaviour, have found to be effective.[35, 36] The traits that have been linked to addictive eating include impulsivity, sensation seeking, and anxiety and depression proneness.[37-41] Findings suggest that individuals with addictive eating may be highly aware of emotions, but lack the skills needed to cope with negative affect.[42] Using personalised coping skills for traits associated with personality and the risk of addictive behaviour in combination with MI, a communication approach used to identify and resolve ambivalence between desired behaviors and actual behaviors to increase motivation,[43] may be affective to facilitate behaviour change in individuals with addictive eating.

Telehealth has been shown to be a strategy to increase reach, with virtual sessions being comparable to face-to-face programs, and to increase access to services without compromising effectiveness.[44] Telehealth will allow participants from anywhere in Australia to participate from home, and will reduce the demands on time and cost of travel.[44] Additionally, telehealth may overcome client-centred barriers by allowing a safe atmosphere for some participants to better engage and discuss more sensitive topics that they would not normally raise.[45] Although the effectiveness of telehealth has not been explicitly explored in populations with addictive eating, recent research demonstrates that telehealth can be as effective as in-person care for the management of mental health conditions,[46] including substance use disorders[47] and eating disorders.[48]

This project builds on a program of work that included an initial feasibility study for the management of addictive eating in adults (Australia New Zealand Clinical Trial Registry ACTRN12619001540101).[24] Results from the initial study indicated that the program was

For peer review only

For peer review only - http://bmjopen.bmj.com/site/about/guidelines.xhtml

feasible in the target population. Feedback, received from program participants and facilitators, identified a need for a greater number of program sessions and improved strategies for increasing retention. As a result, the program was further refined with consumers using an integrated knowledge translation (iKT) framework.[49] This co-design phase included consumers with lived experience, as well as health professionals from a range of disciplines to ensure the culmination of multidisciplinary evidence-based strategies were included. This was unique as previous reports omit this co-design step or are siloed in their approach.[49] The co-design process used a series of interviews and workshops to gain input into the program overview, aims, content and materials. Subsequent changes were made to the program content, language used, and materials were created or refined to improve acceptability. The resultant behaviour change intervention, the TRACE (Targeted Research on Addictive and Compulsive Eating) program, is a complex intervention and previously described using the Medical Research Council TiDier (Template for Intervention Description and Replication) checklist for complex interventions.[50] (See [49] for the TIDieR checklist of the intervention).

To the authors knowledge, the TRACE program is the first MI-based telehealth intervention used in combination with personalised coping skills training for the management of addictive eating in adults. The aim of the current study is to determine the efficacy of a telehealth intervention (active intervention) to reduce symptoms of addictive eating in adults, relative to passive intervention and control (no intervention) groups. It is hypothesised that both the active and passive intervention groups will achieve a reduction in addictive eating symptoms relative to the control group. Potential moderators (e.g., participant sociodemographics) and mediators (e.g., physical activity, diet, and sleep) of intervention efficacy will also be evaluated.

METHODS

Study trial design

For peer review only - http://bmjopen.bmj.com/site/about/guidelines.xhtml

The TRACE program is a randomised controlled trial with three parallel arms. Assessments will be carried out at three timepoints: 1) baseline (pre-intervention), 2) 3-month post-baseline (primary time point), and 3) 6-month post-baseline follow-up assessment. This project was approved by The University of Newcastle Human Research Ethics Committee (H-2021-0100) and prospectively registered with the Australian New Zealand Clinical Trials Registry (ACTRN12621001079831). The study protocol was developed in accordance with the Standard Protocol Items: Recommendations for Interventional Trials (SPIRIT) guidelines.[51] The design, conduct and reporting of the studies will adhere to the CONSORT (Consolidated Standards of Reporting Trials) guidelines.[52] All participants will provide informed electronic consent (see Supplementary Material 1 for a copy of the consent form) to participate and can withdraw at any time for any reason. The funding bodies had no role in the design, conduct or reporting of the study.

Setting

The active intervention will be delivered via telehealth sessions, conducted in Australia, and supported by a program workbook, and website containing materials relevant to the intervention.

Recruitment

Participants will be recruited using a range of strategies including media releases, advertising via local and national newspapers, and social-media releases. Informed by our iKT process, a range of recruitment videos (tailored for gender) were also created in addition to written material which will be released via Twitter and Facebook. A non-probability sampling technique (voluntary response sampling)[53] will be used, and recruitment will continue until the desired number of participants is achieved. Recruitment commenced in August 2021 and was completed in April 2022. Recruitment materials will direct individuals to the study information sheet and eligibility survey. The eligibility survey takes approximately 15 mins to

For peer review only

For peer review only - http://bmjopen.bmj.com/site/about/guidelines.xhtml

complete (Table 1). Online informed consent will be obtained prior to completing the eligibility survey.

Eligibility

To be eligible for inclusion in the study individuals must:

1. Be aged between 18 years and 85 years
2. Endorse ≥ 3 symptoms on the Yale Food Addiction Scale 2.0 (i.e. exhibiting mild to severe addictive eating)[6]
3. Have a self-reported weight and height consistent with a body mass index (BMI) ≥ 18.5 kg/m^2
4. Be competent in the English language
5. Live in Australia
6. Have access to the internet

Individuals will be excluded from participating in the study if they:

1. Are pregnant or lactating
2. Report having a severe mental illness (including schizophrenia or bipolar disorder) or have a health condition that necessitates taking medications which affect dietary intake or weight status
3. Report purging behaviours as identified by the Eating Disorder Examination Questionnaire – Short form (EDE-QS)[54]

Methodological considerations for eligibility criteria: The eligibility screener excludes individuals with a BMI below 18.5kg/m^2. This measure was put in place to reduce the likelihood of recruiting participants with at-risk restrictive eating practices that may be influencing a relatively low weight status. The value of <18.5 kg/m^2 was chosen as this is below the current healthy weight range in national guidelines for Australians[55] and The Centre of Disease Control and Prevention (CDC) in the USA.[56] Additionally, the eligibility screener includes the

For peer review only - http://bmjopen.bmj.com/site/about/guidelines.xhtml

Eating Disorder Examination Questionnaire Short Form (EDE-QS).[54] This 12-item validated tool is commonly used to identify potential eating disorders. Based on the research team consensus, individuals who have compensatory behaviours such as bingeing/purging (specifically asked in question 7 on the EDE-QS), who may be at risk of an eating disorder and are medically compromised, will be deemed not eligible for the current study. Purging is related to higher levels of appearance dissatisfaction, anxiety and depressive symptoms and self-concept instability.[57, 58] As per the ethics protocol, participants endorsing any response to this question, indicating these compensatory behaviours will be excluded from the study. The tools for eating disorders and psychological health[54, 59-63] used in the study have been widely used in research in the areas of eating disorders, dietary interventions, substance use and mental health and are considered standard tools for their specific measures. Study information as well as at completion of surveys participants are provided with contact information if they experience or further assistance with health behaviours.

Table 1. Schedule of measurements

**Variable**	**Instrument**	**Enrolment**	**Timepoint post allocation**		
**Primary study**		Eligibility Screening	t_1 Baseline	t_2 3-months	t_3 6-months
**Sample characteristics**					
Demographics	Age, sex, postcode, mental health status	✓			
Socioeconomic factors	Education, income, marital status, employment status, occupation and living/accommodation status	✓			
Anthropometrics	Self-report height and weight	✓		✓	✓
Smoking and substance use	Alcohol, Smoking and Substance Involvement Screening Test - Version 3.0[59]		✓	✓	✓
Purging behaviours	Eating Disorder Examination Questionnaire Short form (EDE-QS)[54]	✓			
**Primary Outcomes**					
Addictive eating symptoms and severity	Yale Food Addiction Scale 2.0[6]	✓		✓	✓
**Secondary Outcomes**					
Dietary intake and quality	Australian Eating Survey[64, 65]		✓	✓	✓
Depression, anxiety and stress	Patient Health Questionnaire-8,[63] Generalized Anxiety Disorder 7,[60] Perceived Stress Scale[61]	✓		✓	✓
**Mediators/moderators**					
Trait/s associated with risk of addictive behaviour	Substance Use Risk Profile Scale[66]		✓		
Eating Behaviours	Eating Disorder Examination Questionnaire 6.0,[62] Binge Eating		✓	✓	✓

For peer review only - http://bmjopen.bmj.com/site/about/guidelines.xhtml

	Scale,[67] Short Inventory of Grazing,[68] Reward-Based Eating Drive Scale[69]				
Participant activation level	Patient Activation Measure 13[70]	✓		✓	✓
Usage and engagement with program website	Google Analytics (Google LLC) to record number of site visits, visit durations, number of page views, and links accessed/resources downloaded		◆—————	—————	—————◆
Usage and engagement with Facebook group	Number of participants to join group; number of views, likes and comments per post manually recorded			◆—————	—————◆
**Other outcomes**					
Quality of life	EQ-5D-5L[71]		✓	✓	✓
Physical activity level	Active Australia Survey[72]		✓	✓	✓
Sleep hygiene behaviours	Pittsburgh Sleep Quality Index[73]		✓	✓	✓
Health care utilisation	Consumer Services Receipt Inventory[74]		✓	✓	✓
'Control' and 'Compulsion' associated with addictive eating	Qualitative analysis of a segment of the first telehealth session		✓		

Eligibility Screening = assessment of inclusion/exclusion criteria, Baseline = pre-intervention, 3-months = immediate post-intervention, 6-months = 3-months post-intervention.

Study procedure

Prospective participants will complete the eligibility survey. This will include demographic questions (e.g., sex, postcode, marital status, level of education, employment status); the Yale Food Addiction Scale 2.0[6] to confirm endorsement of ≥ 3 addictive eating symptoms; the EDE-QS[31] to confirm the absence of purging behaviours. While not necessary to determine eligibility, the Patient Health Questionnaire-8 (PHQ-8),[63] Generalized Anxiety Disorder-7 (GAD-7),[60] Perceived Stress Scale-4,[61] Patient Activation Measure 13,[70] and two questions relating to previous treatments sought for addictive eating, will also be completed by potential participants. These questions have been specifically added to extend our previously reported research[75] regarding the types of individuals recruited into interventions for addictive eating.

Participants deemed eligible will proceed to the online consent form (Figure 1. Overview of study schedule). Participants will be given a two-week period to consider participation. After this time, a member of the research team will contact any individuals via email who have not

For peer review only - http://bmjopen.bmj.com/site/about/guidelines.xhtml

completed the consent form to determine their interest in participating. Following this, no other contact will be made. Participants who provide electronic written consent will complete the baseline assessment surveys measuring dietary intake and eating habits, traits associated with personality and risk of addictive behaviour, quality of life and healthcare service utilisation (Table 1. Schedule of measurements). The surveys take approximately 40 minutes to complete. On completion of baseline surveys, participants will be randomly allocated to one of three groups (Group 1: active intervention; Group 2: passive intervention; or Group 3: control; see *Intervention* description) and informed of their group allocation via email.

Figure 1. Overview of the study schedule

Following randomisation (see *Randomisation and Blinding*), a member of the research team will contact participants in Group 1 via telephone or email to arrange an appointment time for their initial telehealth session. Groups 1 and 2 will be emailed a copy of the program workbook (printable and fillable PDF versions); a hard copy is available for participants on request; and be provided with password protected access to the program website at this time. Telehealth sessions 2 – 5, for participants allocated to the active intervention group (Group 1), will be arranged during their first telehealth session.

Participants from all three groups will receive results from the eligibility and/or baseline surveys by the research team via email. On survey completion, Groups 1 and 2 will receive feedback on dominant trait/s that may be associated with increased risk for addictive behaviours (e.g., anxiety-proneness, impulsivity-proneness); symptoms of addictive eating; dietary, caffeine and alcohol intake; sleep hygiene and physical activity levels. At this timepoint, Group 3 will only receive feedback on dietary intake via email. At 6-months post study commencement, Group 3 will be provided with feedback on trait/s associated with personality and risk of addictive behaviour; symptoms of addictive eating; sleep hygiene and physical activity levels, along with access to the workbook and website (the passive intervention that Group 2 received

For peer review only - http://bmjopen.bmj.com/site/about/guidelines.xhtml

at baseline). To ensure consistency across participants, email templates and standardised reports will be used by the research team. Group 2 will be guided with written instructions in their workbook on how to utilise their survey results to allow personalised goal setting regarding their dietary intake and eating patterns.

The primary and secondary outcomes will be assessed at 3-months (primary endpoint, immediate post-active intervention period) and 6-months (follow-up) where participants will complete post-program surveys (Table 1. Schedule of measurements). Participants will be sent reminder emails to complete their surveys. They will be reminded a maximum of three times at each time point. If no contact is received after such time, no further contact will be made. Participants will be remunerated with a gift voucher to the value of AUD20 at the completion of baseline, 3-month and 6-month surveys, corresponding to a maximum of AUD60 per participant over the course of the study.

Randomisation and Blinding

Following completion of baseline assessments, participants will be stratified into 4 groups by sex and mental health status (presence or absence, based on either depression, scale PHQ-8 scores ≥15 or below 15, or anxiety scale GAD-7 scores ≥11 or below 11). Participants within each of these four groups will be randomised to one of the three study groups in equal ratios using permuted block randomisation, with block sizes of six. Randomisation will promote group balance on these variables shown to be important in past cross-sectional research (for example, [7, 8, 10, 75]). The randomisation sequence will be generated by an independent statistician and implemented by a designated study co-ordinator. The allocation list will be stored in a password protected computer file and accessed only by the study co-ordinator.

Due to the telehealth nature of the active intervention, blinding of participants and dietitians to intervention group allocation in this study will not be possible. However, several strategies will be employed to reduce the risk of bias. First, participants will only be provided with partial

For peer review only - http://bmjopen.bmj.com/site/about/guidelines.xhtml

information on the study hypotheses. Second, all communication between participants and research staff during the period of intervention (i.e., scheduling concerns, questions regarding the intervention) will be done directly between participants and the 'study co-ordinator' or their respective 'telehealth clinician'. Lastly, statistical analyses will be conducted by researchers who are blind to group allocation prior to analysis.

Intervention

The intervention study arms are:

Group 1. Active intervention: targeting change in addictive eating behaviours using a multicomponent clinician led approach (telehealth sessions, program workbook and program website)

Group 2. Passive intervention: targeting change in addictive eating behaviours using a multicomponent self-guided approach (program workbook and program website)

Group 3. Control: dietary feedback, via paper-based report, provided at baseline and participants follow their usual dietary pattern for six months.

The comparator groups were chosen to provide a passive delivery option of the program which would be consistent with a self-guided Cognitive Behaviour Therapy approach (Group 2), and a control group consistent with a standard version of dietary feedback (Group 3). The control group is not a wait list control, however participants in this group will be offered access to the passive intervention (i.e., program workbook and program website) after the completion of the 6-month assessment.

TRACE Active Intervention (Group 1): Participants will receive five standardised one-on-one telehealth/phone sessions with an Accredited Practising Dietitian, with training in behaviour change and eating disorders, over a 3-month period (i.e., weeks 1, 2, 4, 8 and 12). Additionally, dietitians leading the intervention delivery will have extensive experience in private practice work and working with clients including those with disordered eating and those

For peer review only

with mental health conditions. Sessions will range from 15-45 mins. Telehealth sessions will be provided via the VSee platform (www.vsee.com). The active intervention uses personalised feedback, skill-building exercises, and goal setting to help individuals reduce their symptoms of addictive eating and improve their dietary intake, and relationship with food (see Table 2 for *Overview of intervention sessions*). The intervention is personalised based on an individual's dominant trait/s associated with personality and risk of addictive behaviour (i.e., the traits: depression proneness, anxiety proneness, sensation proneness and/or impulsivity proneness; measured via The Substance Use Risk Profile Scale[37] which the individual scores the most highly for) and addresses a range of factors that influence behaviour, both internal and external. Further, dominant trait/s associated with personality and risk of addictive behaviour are mapped to specific coping skill strategies which are in turn incorporated into the goal setting process. As part of session 1, the first 15 mins of the consultation will be audio recorded to enable qualitative analysis of responses to standardised questions regarding two elements of 'control' and 'compulsion' around the participant's food intake. These two themes were previously identified, through thematic analysis of the feasibility study data,[76] as having an influential relationship with addictive eating behaviours. On completion of the five telehealth sessions, participants will be invited to join a closed Facebook group from 3-months post commencement of the intervention until the 6-month outcome survey measures are conducted. Joining the Facebook group is voluntary.

For peer review only

Table 2. Overview of intervention sessions

Session	Session aims
**1) Personality (Week 1: 45 mins)**	• Introduce the intervention<br>• Determine participant's main concerns with their food intake<br>• Provide feedback on baseline scores of addictive eating<br>• Discuss what this means when attempting and preparing to make changes<br>• Provide feedback on traits associated with personality and risk of addictive behaviour

For peer review only - http://bmjopen.bmj.com/site/about/guidelines.xhtml

Session	Session aims
	• Discuss how personality traits may relate to food intake and addictive eating, and what this means for the individual<br>• Discuss coping strategies based on personality traits and complete '*Urge Surfing*' activity<br>• Introduce '*Distraction List*'<br>• Set homework task: choose and practice 2 coping strategy exercises<br>• Provide session summary
**2) Food (Week 2: 45 min)**	• Review session 1<br>• Check in for episodes of overeating<br>• Discuss progress with homework task - coping strategies<br>• Provide feedback on dietary intake<br>• Discuss core vs non-core food intake (Optional: discuss alcohol intake)<br>• Develop 3 nutrition goals using *SMARTER Goal Checklist*<br>1) Positive – increase core foods<br>2) Reduction – decrease non-core foods<br>3) 'Eating awareness' – using strategies to delay or halt overeating<br>• Discuss enablers/barriers when making changes to eating habits<br>• Discuss '*No Money No Time*' website (www.nomoneynotime.com.au)<br>• Discuss '*Practical Strategies to Achieve Goals*'<br>• Set homework task: complete '*Triggers for Overeating Checklist*'<br>• Provide session summary
**3) Skills (Week 4: 30 min)**	• Review session 2<br>• Assess progress with SMARTER goals<br>• Check in for episodes of overeating<br>• Discuss homework task - '*Triggers for Overeating*'<br>• Explore strategies to overcome triggers, building on previous coping strategies and '*Practical Strategies to Achieve Goals*'<br>• Discuss and determine a '*food line*' to identify when eating is no longer enjoyable or not tasting food<br>• Discuss strategies to stay below the 'food line'<br>• Set homework task: complete '*Mood Monitor*' worksheet

For peer review only - http://bmjopen.bmj.com/site/about/guidelines.xhtml

Session	Session aims
	• Provide session summary
**4) Confidence (Week 8: 30 min)**	• Review session 3<br>• Discuss progress with plan to stay below 'food line' and for episodes of overeating<br>• Explore enablers/barriers to achieving goals<br>• Discuss homework task - '*Mood Monitor*', and explore emotions that participant has difficulty coping with<br>• Discuss seeing emotions differently<br>• Explore coping strategies for difficult emotions<br>• Discuss importance of sleep, physical activity, and responsible intake of alcohol for emotional health<br>• Discuss implementing coping skills plan to achieve SMARTER goals (i.e. consolidate information from sessions 1 – 4)<br>• Set homework task: practice implementation of coping skills plan to achieve goals<br>• Provide session summary
**5) Moving forward (Week 12: 20 mins)**	• Review session 4<br>• Check in/briefly problem solve and encourage participant to continue with goals and strategies<br>• Discuss topics from previous sessions (participant led)<br>• Reassess confidence to achieve goals<br>• Provide final *Addictive Eating Action Plan*<br>• Discuss how support group on Facebook works and encourage sign up

Participant Workbook and Program Website: Participants will have access to a participant workbook and password protected access to a study specific website, both built for the study to support the materials discussed in the intervention sessions. To further facilitate the co-design process, the workbook and website content was piloted with end users (n=2) with lived experience of addictive eating, who participated in the iKT interviews/workshops. The end users reported the workbook and website to be highly usable in terms of the content, and the language used throughout as appropriate with only minor modifications made. Additionally, the piloting process allowed the estimated time to complete each workbook module to be calculated.

For peer review only

Program Workbook: The workbook consists of five modules: 1) Personality; 2) Food; 3) Skills; 4) Confidence; and 5) Moving forward. The content of the five modules mirrors that of the telehealth sessions. The workbook also contains reflective activities/worksheets, discussed during the telehealth sessions, for the participants to complete. These elements were deemed important during the iKT process. The amount of time spent completing activities in the workbook each week, between telehealth sessions, will take approximately 30 - 60 minutes. However, the time to complete each module may vary from person to person, and participants are advised to work through the workbook at a pace that is right for them.

Program Website: The website includes the following pages: 1) Home/Landing page: brief information about the program and login; 2) Dashboard: navigation page to access each of the program's module pages; 3) Module pages: each of the five modules within the intervention has a separate page on the website. This includes additional resources to complement the telehealth sessions and workbook; and 4) About us: brief information about the research/clinician team behind the program, including contact information (email). The website will be available for a period of 12 months from study commencement. All data captured from the website will be encrypted and stored securely on a server.

Program Facebook Group: This is a voluntary part of the study which aims to further support participants with behaviour change. The Facebook forum is set up as a private Facebook group. Participants can use their standard Facebook login, or alternatively, create a new login (a pseudo account) that does not identify them if they wish to remain anonymous. Participants will be prompted with information related to the intervention for the 3-month duration in the form of short posts, blogs, and polls. The Facebook group will allow participants to engage with other participants from the program, as well as serve as a communication method to remind participants about assessments for the study.

For peer review only - http://bmjopen.bmj.com/site/about/guidelines.xhtml

The Facebook forum has the following restrictions: 1) Membership will be by invitation only; 2) The group will not appear in search results or the participants Facebook profile; and 3) Only group members will be able to see the group information and group posts. Participants will be advised of the appropriate use of language and etiquette for using the social media/discussion forum in the workbook and reminded at the final telehealth session. The Facebook group will be moderated by a member of the research team via the TRACE research Facebook account.

Intervention fidelity: A detailed clinician manual will be used by the dietitian for all telehealth sessions to maintain treatment fidelity. Dietitians administering the intervention will be trained by the principal investigator prior to study implementation. Dietitians will also follow each session as outlined in the manual and keep a dietitian log of participants telehealth sessions. Further, five participants allocated to Group 1, with their consent, will have all their telehealth sessions audio recorded. The dietitian log and audio recordings will be reviewed by an independent researcher to ensure the intervention was delivered as intended. Regular supervisory meetings will be conducted with the dietitians and program coordinator led by the principal investigator. Participant adherence to the intervention will be assessed by a session attendance checklist completed by a member of the research team. Dietitians administering the telehealth sessions will monitor completion of homework tasks and workbook activities at the start of telehealth sessions 2 to 5. Assistance will be provided by the dietitian at this time if participants experienced any difficulties completing the homework tasks/activities. Additionally, to assist with adherence, on completion of each telehealth session the dietitian will email a personalised 'Addictive Eating Action Plan', completed on a standardised template, to the participant.

TRACE Passive Intervention (Group 2): Participants will receive the intervention via self-guided approach, with access to the five-module workbook and website (as described above), but without the telehealth consults. The content of the workbook modules mirrors the content of the five telehealth sessions. In addition to the written materials provided, the workbook

For peer review only - http://bmjopen.bmj.com/site/about/guidelines.xhtml

contains spaces for reflective activities, documenting goals and monitoring progress. Participants will be asked, on receipt of the workbook, to complete the workbook within a 3-month period. The proportion of the workbook completed by participants in the passive intervention arm will not be monitored. Following the 3-month self-guided learning period, participants will be invited to join the closed Facebook group as described above.

Control (Group 3): Participants will receive personalised dietary feedback on baseline surveys, provided by an automated report, generated from the Australian Eating Survey. This is consistent with standard dietary feedback from a dietitian. Participants in the control group will be offered access to the participant workbook and study website after the completion of the 6-month assessment.

Patient and public involvement

Consumer (i.e., individuals with lived experience of addictive eating who participated in the pilot study) input was received on the pilot version of the intervention (FoodFix process evaluation[24]) that directly guided the enhancement of the TRACE telehealth sessions. The TRACE program workbook and website for the current study was developed following the pilot study. A sample of consumer representatives (individuals with lived experience of addictive eating and healthcare experts including clinicians and managers), independent of those involved in the pilot study, were involved in the review of the program and program materials.[49]

Consumer representatives were interviewed to:

- Identify what individuals with addictive eating need and want more accurately
- Gather information about what works well and what needs improving, first-hand from consumers who may use them
- Openly consider different or opposing views about aspects of the research project

For peer review only - http://bmjopen.bmj.com/site/about/guidelines.xhtml

- Test resources during development and refine resources making sure they will work well in practice
- Detect any unforeseen consequences of a particular decision or direction that has been made regarding the project
- Gain support of consumers to implement changes to the research project

The opinion of consumers has been considered to create a program that:

- Aligns to the needs of the people it is trying to help i.e., individuals with addictive eating
- Is beneficial in terms of delivering meaningful outcomes for individuals with addictive eating
- Is conducted in a way that is sensitive to peoples' needs

Consumers were not involved in the design of the current study, the selection of outcome measurements, research questions or the recruitment of additional participants. However, consumers were involved in the overall concepts employed in the study and may be called upon at the dissemination stage. For example, to review plain language summaries of the results, provide advice on ways to communicate/translate our findings, or present our findings to the community. Participants of the current study can request a plain English summary of the study outcomes on its completion.

Outcome measures

All outcome measures are completed at baseline, 3 months (immediate post-active intervention period) and 6 months (follow-up) via online surveys. The same survey tools will be used at each time point. Participants will receive assessment reminders by email. (Reference to where data collection forms can be found is included in Supplementary Material 2)

For peer review only

Primary outcomes

Addictive eating symptoms and severity: The Yale Food Addiction Scale (YFAS 2.0)[6] will be used to assess the change in addictive eating symptomatology and severity. The YFAS 2.0 is a validated self-report 35-item questionnaire. The YFAS 2.0 asks participants to think of specific foods they have had difficulty controlling the consumption of within the past 3 months (e.g., ice cream, chocolate, chips, hamburgers). The YFAS 2.0 provides an addictive eating symptom score ranging from zero to 11. Additionally, two items assess clinically significant impairment or distress from eating. A 'food addiction diagnosis' can be given when ≥2 symptoms are endorsed, and clinically significant impairment or distress is present. However, for the purpose of this study a 'food addiction diagnosis' will not be given, and severity of addictive eating will be classified in accordance with YFAS scoring instructions as follows: "mild" = 3 symptoms, "moderate" = 4-5 symptoms or "severe" ≥6 symptoms. The YFAS 2.0 has been found to be a robust and psychometrically sound measure of addictive eating symptomatology in non-clinical[2, 77] and clinical populations with good test/retest validity.[78] Preliminary evidence[23, 24] suggests that YFAS scores are sensitive to change and are decreased after intervention.

Secondary outcomes

Dietary intake and quality: Changes in dietary intake and quality will be measured using the Australian Eating Survey (AES).[64] The following dietary outcomes will be measured: (1) core foods and non-core foods percentage contribution to total energy intake; (2) average daily energy intake, proportion of total energy intake contributed by macronutrients, micronutrient intakes; and (3) overall diet quality. The AES is a validated 120-item semi-quantitative Food Frequency Questionnaire that assesses usual food and nutrient intakes over the previous 3-6 months. The AES includes a comprehensive list of foods, including drinks, milk and dairy foods, breads and cereals, sweet and savoury snacks, main meals, other foods, vegetables and fruit. Frequency response options for each food, or food type, range from 'never' to '≥7 times per day'. The AES has been assessed for comparative validity relative to weighed food

For peer review only - http://bmjopen.bmj.com/site/about/guidelines.xhtml

records and for fruit and vegetable intakes using plasma carotenoids.[64, 65] Standard portion sizes for adult men and women have been determined for each AES item in the survey, using data from the most recent Australian National Nutrition Survey. The food and beverage weight per serving, used in the calculation of food group servings (as serves per day) is consistent with sizes specified in the Australian Guide to Healthy Eating.[64, 65, 79] Nutrient intakes from the AES Food Frequency Questionnaire were computed using data in the AUSNUT 2011–13 database.[80] The AES also provides an Australian Recommended Food Score (ARFS), derived from a subset of 70 AES questions, as a measure of diet quality that reflects the overall healthiness and nutritional quality of an individual's usual eating pattern.[65] The ARFS is based on the frequency of consumption of core foods, recommended in the Australian Dietary Guidelines,[81] with foods awarded one point for a consumption frequency of ≥once per week. The total score is calculated by summing the points for each item and scores can range from zero to 73, with higher scores awarded for greater dietary variety.[65]

Depression, anxiety and stress: Changes in symptom scores for depression, anxiety and stress will be measured using the Patient Health Questionnaire (PHQ-8),[63] the Generalized Anxiety Disorder 7 (GAD-7)[60] and the Perceived Stress Scale (PSS-4),[61] respectively. The PHQ-8 is a validated self-report 8-item tool that asks the individual to rate the severity of depressive symptoms over the past two weeks from 0 (‘not at all’) to 3 (‘nearly every day’). Total scores for the 8 items range from 0 to 24, and severity will be determined using the following cut-offs: 0-4 = minimal, 5-9 = mild, 10-14 = moderate, 15-19 = moderately severe, and 20-24 = severe.[63] The GAD-7 is a validated self-report 7-item tool that asks the individual to rate the severity of symptoms over the past two weeks from 0 (‘not at all sure’) to 3 (‘nearly every day’). GAD-7 total scores range from 0 to 21, and severity is determined using the following cut-offs: 0-5 = mild, 6-10 = moderate, 11-15 = moderately severe, and 15-21 = severe.[60] The PSS-4 is a validated self-report 4-item tool that assesses the degree to which a person perceives life as stressful.[61] The questions have been designed to assess how unpredictable, uncontrollable, and overloaded a person feels their life to be. Frequency over

For peer review only - http://bmjopen.bmj.com/site/about/guidelines.xhtml

the previous month is rated on a five-point Likert scale ranging from ‘Never’ to ‘Very often’. PSS-4 total scores range from 0 to 16, and higher scores indicate greater stress.[61] Currently, there is no established cut-off for the PSS-4 score to indicate adverse levels of stress.

Other outcomes

A selection of other outcomes was chosen based on co-occurring health conditions (see Table 1 for schedule of measurements).

Quality of Life: Changes in subjective quality of life will be measured using the EQ-5D-5L.[71] The EQ5D-5L is a validated self-report 5-item tool to assess health-related quality of life. A descriptive system comprising five dimensions: mobility, self-care, usual activities, pain/discomfort, and anxiety/depression. Each dimension has 3 levels: no problems, some problems, and extreme problems (labelled 1–3). Participants are asked to indicate their health state by ticking the box next to the most appropriate statement in each of the five dimensions. This decision results into a 1-digit number that expresses the level selected for that dimension. The digits for the five dimensions can be combined into a 5-digit number that describes respondent’s health state. The EQ-5D-5 L will be analysed to produce an index score between 0 (state of death) and 1 (perfect health).

Physical activity level: Changes in physical activity level will be measured using the Active Australia Survey (AAS).[72] The AAS is a validated self-report tool containing eight core questions to assess participation (hours/mins per week) in moderate and vigorous intensity physical activity and walking for recreation, over the previous week.

Sleep hygiene behaviours: Changes in sleep hygiene behaviours will be measured using the Pittsburgh Sleep Quality Index (PSQI).[73] The PSQI is a validated self-report survey with 19 self-rated items and 5 items rated by the bed partner or roommate (if applicable). The tool assesses seven components of sleep to provide one global score. Components measured

For peer review only - http://bmjopen.bmj.com/site/about/guidelines.xhtml

include 1) Subjective sleep quality, 2) Sleep latency, 3) Sleep duration, 4) Habitual sleep, 5) Sleep disturbances, 6) Use of sleeping medication, and 7) Daytime dysfunction. The overall global score of sleep quality will be calculated, and the subcomponents reported.

Health care utilisation: For the purpose of conducting a cost analysis the Consumer Services Receipt Inventory (CSRI)[74] will be completed by participants at each time point. The CSRI is an adaptable tool that ensures the format, language, scope and content is compatible with the research aims, context, participants' likely circumstances, and the quantity and precision of information required.[82] Health care utilisation is captured through self-report and includes information on the number of appointments and type of health care services used in the preceding 3 months.

Cost analysis: A cost-consequence analysis will be conducted including calculating the cost of each intervention (i.e., active, passive and control) and reporting intervention costs alongside mean change outcomes. Intervention costs will be recorded in terms of cost of intervention development, intervention delivery and the operating costs of the RCT. Outcomes to be reported as part of the cost analysis will include mean change in addictive eating symptom scores assessed using the YFAS (i.e. the primary outcome), as well as mean change in the number of health care appointments in the past 3-months assessed using the CSRI, and mean change in Quality Adjusted Life Years (QALYs) assessed using the EQ-5D-5L. This approach was selected to provide a comprehensive and transparent overview of intervention costs, given the lack of cost analysis data in this area of research.[83, 84]

Mediators/Moderators

The following potential mediators and moderators of intervention efficacy will be examined:

Trait/s associated with personality and risk of addictive behaviour: Participant's will complete the Substance Use Risk Profile Scale (SURPS)[66] at baseline to determine their

For peer review only - http://bmjopen.bmj.com/site/about/guidelines.xhtml

dominant trait/s. The SURPS is a validated self-report 23-item survey that assess four traits associated with increased risk for addictive behaviours (Impulsivity proneness, Sensation proneness, Depression proneness, and Anxiety proneness).

Eating behaviours: Eating behaviours that have been shown to have overlap with addictive eating will be measured. This includes eating disorders, binge eating, grazing behaviours and reward driven eating. Eating disorders will be measured using the Eating Disorder Examination Questionnaire 6.0 (EDEQ-6.0)[62] The EDEQ-6.0 is a validated self-report 28-item questionnaire that assesses the occurrence and frequencies of key eating disorder behaviours with cognitive subscales related to eating disorders (restraint, eating concern, shape concern, and weight concern) and behavioural symptoms related to these concerns (e.g. frequency of binge eating, vomiting, use of laxatives or diuretics, and overexercise). Subscale and global scores reflect the severity of eating disorder psychopathology. Binge eating will be measured using the Binge Eating Scale (BES).[67] The BES is a validated self-report 16-item questionnaire to assess the presence of certain binge eating behaviours, over the past 28 days, which may be indicative of an eating disorder. Each item contains 3-4 statements about behaviours, thoughts, and emotional states. Grazing behaviours will be measured using the Short Inventory of Grazing (SIG).[68] The SIG is a validated self-report 2-item measure to assess 1) the presence and frequency of grazing in general, and 2) the presence and frequency of grazing accompanied by a sense of loss of control. Reward driven eating will be measured using the Reward-Based Eating Drive Scale (REDX-5).[69] The REDX-5 is a validated self-report 5-item questionnaire, in 5-point Likert scale format from 1 (strongly disagree) to 5 (strongly agree), that assesses reward-driven eating (loss of control over eating, lack of satiety, and preoccupation with food).

Participant Activation Level: Participant's underlying knowledge, skills and confidence in managing their addictive eating behaviours and overall health will be measured using the Patient Activation Measure (PAM-13).[85] The PAM-13 is a validated self-report 13-item scale

For peer review only - http://bmjopen.bmj.com/site/about/guidelines.xhtml

that draws on concepts such as health locus of control, self-efficacy in managing health behaviours and readiness to change health behaviours.[70, 86] Higher PAM-13 scores indicate that individuals have higher levels of activation, and understand their role in the self-management process and feel capable of fulfilling that role.[87] Research has demonstrated that a single point change in PAM score is meaningful.[88]

Engagement and use of the program website and Facebook group: Interaction with the website will be objectively tracked throughout the study (baseline to 6 months i.e., timepoints 1 to 3) using Google Analytics (Google LLC). Measures of engagement and usage will include number of website visits, website visit duration, number of page views and links accessed/resources downloaded.

Interaction with the Facebook group will be measured throughout the post-intervention period (3 to 6 months from baseline i.e., timepoints 2 to 3). Measures of engagement and usage will include number of participants to join the Facebook group, and number of views, likes and comments per post.

Study sample characteristics

Sociodemographic data will be collected by online questionnaire at baseline. Participants will provide information on their age, sex, marital status, postal code, years of education, employment status and current living situation. Index of Relative Socio-Economic Disadvantage (IRSD) score,[89] based on the Australian Bureau of Statistics census data and reflecting a proxy index of socioeconomic status, will be determined by postal code of residence. Current smoking and substance use will be measured using the Alcohol, Smoking and Substance Involvement Screening Test - Version 3.0.[59] Additionally, previous treatment sought for overeating from health professionals or products used to treat overeating will be collected.

For peer review only - http://bmjopen.bmj.com/site/about/guidelines.xhtml

Anthropometric data (self-reported height and weight) will be collected by online questionnaire at baseline. BMI will be calculated using standardised techniques and categorised according to the World Health Organization adult cut-off points.[90]

Sample size

The sample size for the study was calculated based on data from the feasibility study,[24] given the absence of other intervention studies. Through guidance with statisticians, a large effect size was chosen and needed to enable the possibility of a clinically meaningful result. A clinically meaningful difference in symptoms of addictive eating was selected as a decrease of 2 symptoms, given this would correspond to a change in severity classification on the YFAS 2.0 tool. To detect a mean 2-unit difference (SD = 2.2) in the YFAS symptoms between the active intervention group and the passive intervention group or control group and using a standardised effect size of d=0.91, a sample size of 32 individuals per group (total sample size n=96) is required to detect this change with a power of 0.90 and a type 1 error rate set at 0.025 to account for multiple testing. However, allowing for a 30% dropout rate from the pilot, a sample size of 46 individuals per group (total sample size n=138) would be required. Therefore, a total sample size of 150 individuals, with 50 per group, was chosen to remain conservative.

Statistical analysis plan

Data analysis will be conducted by a researcher blinded to the intervention conditions. Descriptive statistics of sample characteristics will be presented. For the primary YFAS outcome a Linear Mixed Model (LMM) will be based on a model with main effects for group (active intervention, passive intervention, control) and time (treated as categorical at levels baseline, 3 and 6 months), and the group-by-time interaction. An unstructured residual covariance structure will be used to allow for correlation between the repeated measurements for a subject. The primary outcome effect will be reported as the difference between means at baseline and 3 months, with a 95% CI for the difference. Mental health condition and BMI will

For peer review only - http://bmjopen.bmj.com/site/about/guidelines.xhtml

be examined for possible moderating effects on the effect size, and if so adjustment for them will be carried out. Secondary descriptive analysis will be carried out to identify whether specific symptoms were predominantly associated with reductions in YFAS score.

A secondary outcome will be a categorical variable, clinically significant change from baseline to 3 months, where significant requires a reduction of 2 or more symptoms in the YFAS. This will be analysed using logistic regression with group being the only factor. Additional secondary outcomes will include dietary outcomes (average daily energy intake, proportion of total energy intake contributed by core foods and non-core foods intakes, macronutrients intakes, micronutrient intakes; and overall diet quality) and mental health status (depression, anxiety and stress scores). These will also be analysed using LMMs as per the approach above. All available data will be used with no imputation of missing values at 3 and 6 months, however baseline scores will be kept. The participants will be analysed according to their allocated randomisation group consistent with an intention-to-treat analysis. Statistical significance will be set at 0.05.

Data management and monitoring

Online survey data will be managed using REDCap electronic data capture tools[91, 92] hosted at the University of Newcastle. REDCap (Research Electronic Data Capture) is a secure, web-based software platform designed to support data capture for research studies, providing 1) an intuitive interface for validated data capture; 2) audit trails for tracking data manipulation and export procedures; 3) automated export procedures for seamless data downloads to common statistical packages; and 4) procedures for data integration and interoperability with external sources.

All data captured from the study website will be encrypted and stored securely on the server. All other data collected will be entered into a password protected central database which is hosted on secure university-based servers, which comply with robust security standards for

For peer review only - http://bmjopen.bmj.com/site/about/guidelines.xhtml

clinical data and are subject to daily backups and regular offsite backups. Only authorised members of the research team will have access to the database. Research staff handling study data are trained in procedures for handling sensitive information, accurate data entry, surveillance and intervention-specific data storage and data archive. Facilitators of the telehealth sessions are responsible for the electronic storage of study forms on the central database. All completed forms will be checked for completeness and accuracy, first by data collectors and later by a member of the research team responsible for data management. Throughout the study period (at 25% and 50% of required participants) approximately 5% of records will be randomly selected for data quality checks of accuracy and completeness by an independent reviewer.

A Data Safety Monitoring Board will not be established for this study as all elements of the intervention have been previously explored and used in interventions. To monitor for potential risks, the study co-ordinator managing the day-to-day conduct of the trial, and facilitators of the telehealth sessions, will report weekly to the Chief Investigator. Oversight concerning the overall conduct of the trial will be provided by our multi-disciplinary research team. This will include regular meetings to review protocol adherence, participant retention rates and safety reports. For the entire study period, any adverse events, of any kind, that might be related to either the trial intervention or trial procedures will be logged in an adverse event log and reported to the Human Research Ethics Committee by the Chief Investigator. To maintain the welfare of participants, with their consent, relevant survey results from the GAD-7[60] and PHQ-8[63] will be sent to the participant's nominated General Practitioner/ health professional if they score in the severe category for either anxiety (GAD-7 scores ≥16) or depression (PHQ-8 scores ≥20) if participants consent to this disclosure.

Study sponsorship and organisation

For peer review only

For peer review only - http://bmjopen.bmj.com/site/about/guidelines.xhtml

The sponsor of the trial is the University of Newcastle, and funding was provided by the National Health and Medical Research Council (NHMRC). The trial will be conducted and evaluated independent of the study sponsor and funder. The study is coordinated independently of the study sponsor and funder, by researchers at the University of Newcastle, Australia with the study overseen by the trial management committee comprising the chief investigators.

Ethics and dissemination

The trial will be undertaken in compliance with the Declaration of Helsinki and approval to conduct the study was received from the University of Newcastle Human Research Ethics Committee (H-2021-0100). This trial adheres to the SPIRIT guidelines for randomised trials protocols[51] and the results will be reported in accordance with CONSORT guidelines (TIDieR checklist and guide[50]). Protocol modifications will be registered with the Ethics Committee and trial register. All participants will provide electronic consent to participate prior to completing the eligibility and baseline surveys. Results of the study will be disseminated via peer-reviewed publications and presentations at national and international conferences and will also form part of student dissertations. Data from the TRACE study may be made available in the future for collaborative research questions. Such requests must be authorised by the principal investigators and the appropriate Human Research Ethics Committees.

Limitations

Limitations of the study include the level of experience required of the dietitians administering the telehealth sessions, which may impact the scalability of the intervention. However, dietitians are highly trained professionals in behaviour change and extra care was taken given the uniqueness of the intervention. The fidelity outcomes assessed as part of the trial will provide important information regarding future implementation. Additional limitations include the exclusion of individuals with severe mental illnesses or complex health conditions. The current intervention is not designed for complex co-morbidities. It is envisaged that for these

For peer review only

For peer review only - http://bmjopen.bmj.com/site/about/guidelines.xhtml

individuals a more complex care model is required where the TRACE program could be implemented alongside other approaches or treatments.

Currently there are few published interventions, run by dietitians and/or other health clinicians, for addictive eating demonstrating the clear need for services and trailing of interventions that may be effective at facilitating changes in addictive eating behaviours. The TRACE program was designed to raise awareness, and support behaviour change, of addictive eating. If successful, our study will provide essential evidence regarding the efficacy of behavioural and dietary improvement in the management of addictive eating, thus allowing for the implementation of management strategies for addictive eating into community and clinical healthcare services. Further, if both the active and passive interventions are found to be effective it will provide evidence of different levels of care that could be utilised within these services.

Author Contributions: TLB conceptualised the study, and TLB, JAS, MW, ML, RC, KMP, AVG, PJH, ALB, LH, SJP, LGW, KC and CEC contributed to the study protocol. TLB, JAS, MW, ML, RC, KMP, AVG, PJH, ALB, LH, SJP, and CEC contributed to the intervention development and design, intervention resources and assessment methodology. JAS wrote the initial manuscript draft. TLB, JAS, MW, ML, RC, KMP, AVG, PJH, ALB, LH, SJP, LGW, KC and CEC contributed to the writing of the final manuscript and/or provided critical comments during revisions. All authors approved the final version prior to submission. TLB, JAS, MW, ML and RC will be responsible for recruitment, data collection and intervention delivery.

Competing interests: None declared.

Funding: This work was supported by the National Health and Medical Research Council (NHMRC) grant number [G1801414].

For peer review only

For peer review only - http://bmjopen.bmj.com/site/about/guidelines.xhtml

References

1. Davis C. From Passive Overeating to ‘‘Food Addiction’’: A Spectrum of Compulsion and Severity. *International Scholarly Research Notices Obesity.* 2013; 435027: 1-20. http://dx.doi.org/10.1155/2013/435027.

2. Gearhardt AN, Corbin WR, and Brownell KD. Preliminary validation of the Yale Food Addiction Scale. *Appetite.* 2009; 52(2): 430-436. https://doi.org/10.1016/j.appet.2008.12.003.

3. Gearhardt AN, Davis C, Kuschner R, et al. The Addiction Potential of Hyperpalatable Foods. *Current Drug Abuse Reviews.* 2011; 4: 140-145. doi: 10.2174/1874473711104030140.

4. American Psychiatric Association, *Diagnostic and statistical manual of mental disorders*. 5th ed. 2013, Arlington: VA American Psychiatric Publishing.

5. World Health Organization. International statistical classification of diseases and related health problems (11th ed.). 2019. https://icd.who.int/.

6. Gearhardt AN, Corbin WR, and Brownell KD. Development of the Yale Food Addiction Scale Version 2.0. *Psychology of Addictive Behaviors.* 2016; 30(1): 113-121. https://doi.org/10.1037/adb0000136.

7. Burrows T, Kay-Lambkin F, Pursey K, et al. Food addiction and associations with mental health symptoms: a systematic review with meta-analysis. *Journal of Human Nutrition and Dietetics.* 2018; 31(4): 544-572. https://doi.org/10.1111/jhn.12532.

8. Pursey K, Stanwell P, Gearhardt A, et al. The prevalence of food addiction as assessed by the Yale Food Addiction Scale: a systematic review. *Nutrients* 2014; 6(10): 4552–4590. https://doi.org/10.3390/nu6104552.

9. Pursey KM, Collins CE, Stanwell P, et al. Foods and dietary profiles associated with ‘food addiction’ in young adults. *Addictive Behaviors Reports* 2015; 2: 41–48. http://dx.doi.org/10.1016/j.abrep.2015.05.007.

For peer review only

For peer review only - http://bmjopen.bmj.com/site/about/guidelines.xhtml

10. Skinner J, Jebeile H, and Burrows T. Food addiction and mental health in adolescents: a systematic review. *The Lancet. Child and adolescent health.* 2021; 5(10): 751-766. https://doi.org/10.1016/S2352-4642(21)00126-7.

11. Sanchez I, Lucas I, Munguía I, et al. Food addiction in anorexia nervosa: Implications for the understanding of crossover diagnosis. *European Eating Disorders Review.* 2022; 30(3): 278–288. https://doi.org/10.1002/erv.2897.

12. Schulte EM and Gearhardt AN. Associations of Food Addiction in a Sample Recruited to Be Nationally Representative of the United States. *European Eating Disorders Review.* 2018; 26(2): 112–119. https://doi.org/10.1002/erv.2575.

13. Pursey KM, Skinner J, Leary M, et al. The Relationship between Addictive Eating and Dietary Intake: A Systematic Review. *Nutrients.* 2021(1): 164. https://doi.org/10.3390/nu14010164.

14. Burrows T, Hides L, Brown R, et al. Differences in Dietary Preferences, Personality and Mental Health in Australian Adults with and without Food Addiction. *Nutrients* 2017; 9 (3): E285. https://doi.org/10.3390/nu9030285.

15. GBD 2017 Diet Collaborators. Health effects of dietary risks in 195 countries, 1990-2017: a systematic analysis for the Global Burden of Disease Study 2017. . *Lancet (London, England),*. 2019; 393(10184): 1958–1972. https://doi.org/10.1016/S0140-6736(19)30041-8.

16. Treasure J, Leslie M, Chami R, et al. Are trans diagnostic models of eating disorders fit for purpose? A consideration of the evidence for food addiction. *European Eating Disorder Review.* 2018; 26(2): 83-91. doi: 10.1002/erv.2578.

17. Wiss DA and Brewerton TD. Incorporating food addiction into disordered eating: the disordered eating food addiction nutrition guide (DEFANG). *Eating and Weight Disorders.* 2017; 22(1): 49-59. doi: 10.1007/s40519-016-0344-y.

18. Burrows T, Skinner J, McKenna R, et al. Food Addiction, Binge Eating Disorder, and Obesity: Is There a Relationship? *Behavioral Sciences.* 2017; 7(3): pii: E54. https://doi.org/10.3390/bs7030054.

For peer review only - http://bmjopen.bmj.com/site/about/guidelines.xhtml

19. Bao K, French EN, Schleyer B, et al. Food addiction is associated with greater objective binge eating and eating disorder psychopathology, and higher body mass index in youth, a meta-analysis. *Psychiatry Research Communications.* 2022; 2(3): 100067. https://doi.org/10.1016/j.psycom.2022.100067.

20. Food Addicts Anonymous. [Homepage]. [cited 2022 24 March]; Available from: https://faacanhelp.org/.

21. Overeaters Anonymous. [Homepage]. [cited 2022 24 March]; Available from: https://oa.org/.

22. McKenna RA, Rollo ME, Skinner JA, et al. Food Addiction Support: Website Content Analysis. *JMIR Cardio.* 2018; 2(1): e10. https://doi.org/10.2196/cardio.8718.

23. Leary M, Pursey KM, Verdejo-Garcia A, et al. Current Intervention Treatments for Food Addiction: A Systematic Review. *Behavioral Sciences* 2021; 11(6): 80. https://doi.org/10.3390/bs11060080.

24. Burrows T, Collins R, Rollo M, et al. The feasibility of a personality targeted intervention for addictive overeating: FoodFix. *Appetite.* 2021; 156(104974). https://doi.org/10.1016/j.appet.2020.104974.

25. Chao AM, Wadden TA, Tronieri JS, et al. Effects of addictive-like eating behaviors on weight loss with behavioral obesity treatment. *Journal of Behavioral Medicine.* 2019; 42(2): 246–255. https://doi.org/10.1007/s10865-018-9958-z.

26. Carbone EA, Caroleo M, Rania M, et al. An open-label trial on the efficacy and tolerability of naltrexone/bupropion SR for treating altered eating behaviours and weight loss in binge eating disorder. *Eating and Weight Disorders.* 2021; 26(3): 779–788. https://doi.org/10.1007/s40519-020-00910-x.

27. Epstein DH, Kennedy AP, Furnari M, et al. Effect of the CRF1-receptor antagonist pexacerfont on stress-induced eating and food craving. *Psychopharmacology.* 2016; 233(23-24): 3921–3932. https://doi.org/10.1007/s00213-016-4424-5.

For peer review only

28. Sevinçer GM, Konuk N, Bozkurt S, et al. Food addiction and the outcome of bariatric surgery at 1-year: Prospective observational study. *Psychiatry Research.* 2016; 244: 159–164. https://doi.org/10.1016/j.psychres.2016.07.022.

29. Murray SM, Tweardy S, Geliebter A, et al. A Longitudinal Preliminary Study of Addiction-Like Responses to Food and Alcohol Consumption Among Individuals Undergoing Weight Loss Surgery. *Obesity Surgery.* 2019; 29(8): 2700–2703. https://doi.org/10.1007/s11695-019-03915-3.

30. Gordon EL, Merlo LJ, Durning PE, et al. Longitudinal Changes in Food Addiction Symptoms and Body Weight among Adults in a Behavioral Weight-Loss Program. *Nutrients.* 2020; 12(12): 3687. https://doi.org/10.3390/nu12123687.

31. Cassin S, Leung S, Hawa R, et al. Food Addiction Is Associated with Binge Eating and Psychiatric Distress among Post-Operative Bariatric Surgery Patients and May Improve in Response to Cognitive Behavioural Therapy. *Nutrients.* 2020; 12(10): 2905. https://doi.org/10.3390/nu12102905.

32. Unwin J, Delon C, Giæver H, et al. Low carbohydrate and psychoeducational programs show promise for the treatment of ultra-processed food addiction. *Frontiers in Psychiatry.* 2022; 13: 1005523. https://doi.org/10.3389/fpsyt.2022.1005523.

33. Carlos LO, Ramos MRZ, Wagner NRF, et al. Probiotic Supplementation attenuates binge eating and food addiction 1 year after roux-en-y gastric bypass: a randomized, double-blind, placebo controlled trial. *Arquivos brasileiros de cirurgia digestiva.* 35: e1659. https://doi.org/10.1590/0102-672020210002e1659.

34. Cassin SE, Sijercic I, and Montemarano V. Psychosocial Interventions for Food Addiction: A Systematic Review *Current Addiction Reports.* 2020; 7: 9–19. https://doi.org/10.1007/s40429-020-00295-y.

35. Hides L, Kavanagh DJ, Daglish M, et al. The Quik Fix study: a randomised controlled trial of brief interventions for young people with alcohol-related injuries and illnesses accessing emergency department and crisis support care. *BMC Emergency Medicine.* 2014; 14(19). https://doi.org/10.1186/1471-227X-14-19.

For peer review only

For peer review only - http://bmjopen.bmj.com/site/about/guidelines.xhtml

36. Hides L, Wilson H, Quinn C, et al. QuikFix: enhanced motivational interviewing interventions for youth substance use. *Advances in Dual Diagnosis.* 2016; 9(2/3): 53-65. https://doi.org/10.1108/ADD-03-2016-0008.

37. Murphy CM, Stojek MK, and MacKillop J. Interrelationships among impulsive personality traits, food addiction, and body mass index. *Appetite.* 2014; 73: 45–50. https://doi.org/10.1016/j.appet.2013.10.008.

38. Davis C. A narrative review of binge eating and addictive behaviors: shared associations with seasonality and personality factors. *Frontiers in Psychiatry.* 2013; 4: 183. https://doi.org/10.3389/fpsyt.2013.00183.

39. Brunault P, Ducluzeau PH, Courtois R, et al. Food Addiction is Associated with Higher Neuroticism, Lower Conscientiousness, Higher Impulsivity, but Lower Extraversion in Obese Patient Candidates for Bariatric Surgery. *Substance Use and Misuse.* 2018; 53(11): 1919–1923. https://doi.org/10.1080/10826084.2018.1433212.

40. Ouellette AS, Rodrigue C, Lemieux S, et al. An examination of the mechanisms and personality traits underlying food addiction among individuals with severe obesity awaiting bariatric surgery. *Eating and Weight Disorders.* 22(4): 633–640. https://doi.org/10.1007/s40519-017-0440-7.

41. Jiménez-Murcia S, Agüera Z, Paslakis G, et al. Food Addiction in Eating Disorders and Obesity: Analysis of Clusters and Implications for Treatment. *Nutrients.* 2019; 11(11): 2633. https://doi.org/10.3390/nu11112633.

42. Bunio LK, Battles JA, and Loverich TM. The nuances of emotion regulation difficulties and mindfulness in food addiction. *Addiction Research and Theory.* 2021; 29(1): 11-17. https://doi.org/10.1080/16066359.2020.1714038.

43. Miller W and Rollnick S. Motivational Interviewing. *3rd ed. New York, NY: Guilford Press.* 2013.

44. Kelly JT, Allman-Farinelli M, Chen J, et al. Dietitians Australia position statement on telehealth. *Nutrition and Dietetics: the journal of the Dietitians Association of Australia.* 2020; 77(4): 406-415. https://doi.org/10.1111/1747-0080.12619.

For peer review only

For peer review only - http://bmjopen.bmj.com/site/about/guidelines.xhtml

45. Reynolds DJ Jr, Stiles WB, Bailer AJ, et al. Impact of exchanges and client-therapist alliance in online-text psychotherapy. *Cyberpsychology, Behavior and Social Networking.* 2013; 16(5): 370–377. https://doi.org/10.1089/cyber.2012.0195.

46. Scott AM, Clark J, Greenwood H, et al. Telehealth v. face-to-face provision of care to patients with depression: a systematic review and meta-analysis. *Psychological Medicine.* 2022; 52(14): 2852–2860. https://doi.org/10.1017/S0033291722002331.

47. Mark TL, Treiman K, Padwa H, et al. Addiction Treatment and Telehealth: Review of Efficacy and Provider Insights During the COVID-19 Pandemic. *Psychiatric Services.* 2022; 73(5): 481-598. https://doi.org/10.1176/appi.ps.202100088.

48. Gorrell S, Reilly EE, Brosof L, et al. Use of Telehealth in the Management of Adolescent Eating Disorders: Patient Perspectives and Future Directions Suggested from the COVID-19 Pandemic. *Adolescent health, medicine and therapeutics.* 2022; 13: 45–53. https://doi.org/10.2147/AHMT.S334977.

49. Leary M, Pursey KM, Verdejo-Garcia A, et al. Designing an online intervention for adults with addictive eating: A qualitative Integrated Knowledge Translation approach. *BMJ Open.* 2022; 12(6): e060196. https://doi.org/10.1136/bmjopen-2021-060196.

50. Hoffmann T, Glasziou P, Boutron I, et al. Better reporting of interventions: template for intervention description and replication (TIDieR) checklist and guide. *BMJ.* 2014; 348: g1687. https://doi.org/10.1136/bmj.g1687.

51. Chan A-W, Tetzlaff JM, Altman DG, et al. SPIRIT 2013 Statement: Defining standard protocol items for clinical trials. *Annals of Internal Medicine.* 2013; 158(3): 200-207. https://doi.org/10.7326/0003-4819-158-3-201302050-00583.

52. Schulz KF, Altman DG, Moher D, et al. CONSORT 2010 statement: updated guidelines for reporting parallel group randomised trials. *BMJ.* 2010; 340(c332). https://doi.org/10.1136/bmj.c332.

For peer review only

For peer review only - http://bmjopen.bmj.com/site/about/guidelines.xhtml

53. Purna Singh A, Vadakedath S, and Kandi V. Clinical Research: A Review of Study Designs, Hypotheses, Errors, Sampling Types, Ethics, and Informed Consent. *Cureus.* 2023; 5(1): e33374. https://doi.org/10.7759/cureus.33374.

54. Prnjak K, Mitchison D, Griffiths S, et al. Further development of the 12-item EDE-QS: identifying a cut-off for screening purposes*. BMC Psychiatry.* 2020; 20(1): 146. https://doi.org/10.1186/s12888-020-02565-5.

55. Australian Government Department of Health. Body mass index (BMI) and waist measurement. 2021 [cited 2022 8 March]; Available from: https://www.health.gov.au/health-topics/overweight-and-obesity/bmi-and-waist.

56. Centers for Disease Control and Prevention (CDC). Healthy Weight, Nutrition, and Physical Activity: Assessing Your Weight. 2021 [cited 2022 8 March]; Available from: https://www.cdc.gov/healthyweight/assessing/index.html.

57. Mond J, Hay P, Rodgers B, et al. Use of extreme weight control behaviors with and without binge eating in a community sample: implications for the classification of bulimic-type eating disorders*. International Journal of Eating Disorders.* 2006; 39(4): 294-302. https://doi.org/10.1002/eat.20265.

58. Mond JJ, Hay PJ, Rodgers B, et al. Correlates of the use of purging and non-purging methods of weight control in a community sample of women*. The Australian and New Zealand Journal of Psychiatry.* 2006; 40(2): 136-142. https://doi.org/10.1080/j.1440-1614.2006.01760.x.

59. WHO ASSIST Working Group. The Alcohol, Smoking and Substance Involvement Screening Test (ASSIST): development, reliability and feasibility*. Addiction.* 2002; 97(9): 1183-1194. https://doi.org/10.1046/j.1360-0443.2002.00185.x.

60. Spitzer RL, Kroenke K, Williams JBW, et al. A Brief Measure for Assessing Generalized Anxiety Disorder (The GAD-7)*. Archives of Internal Medicine.* 2006; 166(10): 1092–1097 https://doi.org/10.1001/archinte.166.10.1092.

For peer review only

For peer review only - http://bmjopen.bmj.com/site/about/guidelines.xhtml

61. Ingram PB 4th, Clarke E, and Lichtenberg JW. Confirmatory Factor Analysis of the Perceived Stress Scale-4 in a Community Sample. *Stress Health.* 2016; 32(2): 173–176. https://doi.org/10.1002/smi.2592.

62. Fairburn C, Cooper Z, and O'Connor M, *Eating disorders examination (16.0D)* in *In: Cognitive behavior therapy and eating disorders*, Fairburn C (Ed.), Editor. 2008, Guilford Press: New York.

63. Kroenke K, Strine TW, Spitzer RL, et al. The PHQ-8 as a measure of current depression in the general population. *Journal of affective disorders.* 2009; 114(1-3): 163-173. https://doi.org/10.1016/j.jad.2008.06.026.

64. Collins CE, Boggess MM, Watson JF, et al. Reproducibility and comparative validity of a food frequency questionnaire for Australian adults. *Clinical Nutrition.* 2014; 33(5): 906-914. https://doi.org/10.1016/j.clnu.2013.09.015.

65. Ashton L, Williams R, Wood L, et al. Comparison of Australian Recommended Food Score (ARFS) and Plasma Carotenoid Concentrations: A Validation Study in Adults. *Nutrients.* 2017; 9(8): 888. http://doi.org/10.3390/nu9080888.

66. Woicik PA, Stewart SH, Pihl RO, et al. The Substance Use Risk Profile Scale: a scale measuring traits linked to reinforcement-specific substance use profiles. *Addictive Behaviors.* 2009; 34(12): 1042-1055. https://doi.org/10.1016/j.addbeh.2009.07.001.

67. Gormally J, Black S, Daston S, et al. The assessment of binge eating severity among obese persons. *Addictive Behaviors.* 1982; 7(1): 47-55. https://doi.org/10.1016/0306-4603(82)90024-7.

68. Heriseanu AI, Hay P, and Touyz S. The short inventory of grazing (SIG): development and validation of a new brief measure of a common eating behaviour with a compulsive dimension. *Journal of Eating Disorders.* 2019; 7(4). https://doi.org/10.1186/s40337-019-0234-6.

69. Vainik U, Han C, Epel ES, et al. Rapidly assessing reward-related eating: The RED-X5. *Obesity.* 2019; 27(2): 325–331. https://doi.org/10.1002/oby.22374.

For peer review only

For peer review only - http://bmjopen.bmj.com/site/about/guidelines.xhtml

70. Hibbard JH, Stockard J, Mahoney ER, et al. Development of the Patient Activation Measure (PAM): conceptualizing and measuring activation in patients and consumers. *Health Services Research.* 2004; 39(4 Pt 1): 1005–1026. https://doi.org/10.1111/j.1475-6773.2004.00269.x.

71. Rabin R and de Charro F. EQ-5D: a measure of health status from the EuroQol Group. *Annals of Medicine.* 2001; 33(5): 337-343. https://doi.org/10.3109/07853890109002087.

72. Australian Institute of Health and Welfare (AIHW). The Active Australia Survey: a guide and manual for implementation, analysis and reporting. 2003; Available from: https://www.aihw.gov.au/reports/physical-activity/active-australia-survey/summary.

73. Buysse DJ, Reynolds CF 3rd, Monk TH, et al. The Pittsburgh Sleep Quality Index: a new instrument for psychiatric practice and research. *Psychiatry Research.* 1989; 28(2): 193–213. https://doi.org/10.1016/0165-1781(89)90047-4.

74. Beecham J and Knapp M, *Costing psychiatric interventions*, in *Measuring Mental Health Needs (2nd edition)*, Thornicroft G, Editor. 2001, Gaskell: London. p. 200-224.

75. Pursey KM, Collins R, Skinner J, et al. Characteristics of individuals seeking addictive eating treatment. *Eating and Weight Disorders.* 2021; 26(8): 2779-2786. https://doi.org/10.1007/s40519-021-01147-y.

76. Collins R, Haracz K, Leary M, et al. No control and overwhelming cravings: Australian adults' perspectives on the experience of food addiction. *Appetite.* 2021; 59(105054). https://doi.org/10.1016/j.appet.2020.105054.

77. Pedram P, Wadden D, Amini P, et al. Food addiction: its prevalence and significant association with obesity in the general population. *PLOS One.* 2013; 8(9): e74832. https://doi.org/10.1371/journal.pone.0074832.

78. Lemeshow AR, Gearhardt AN, Genkinger JM, et al. Assessing the psychometric properties of two food addiction scales *Eating Behaviors.* 2016; 23: 110–114. https://doi.org/10.1016/j.eatbeh.2016.08.005.

For peer review only

For peer review only - http://bmjopen.bmj.com/site/about/guidelines.xhtml

79. National Health and Medical Research Council (NHMRC). Australian Guide to Healthy Eating. 2017; Available from: https://www.eatforhealth.gov.au/guidelines/australian-guide-healthy-eating.

80. Food Standards Australia New Zealand (FSANZ). AUSNUT 2011–13–Australian Food Composition Database. 2014 [cited 2021 31 May]; Available from: http://foodstandards.gov.au/.

81. National Health and Medical Research Council. Australian Dietary Guidelines. Canberra. 2013; Available from: www.nhmrc.gov.au/guidelines-publications/n55.

82. Personal Social Services Research Unit (PSSRU). 2022 [cited 2022 14 April]; Available from: https://www.pssru.ac.uk/csri/client-service-receipt-inventory/.

83. Mauskopf JA, Paul JE, Grant DM, et al. The role of cost-consequence analysis in healthcare decision-making. *Pharmacoeconomics.* 1998; 13(3): 277-288. https://doi.org/10.2165/00019053-199813030-00002.

84. Hunter R and Shearer J. Cost-consequences analysis - an underused method of economic evaluation. [cited 2022 8 March]; Available from: https://www.rds-london.nihr.ac.uk/wpcms/wp-content/uploads/2018/09/Cost-consequences-analysis-an-underused-method.pdf.

85. Hibbard JH, Mahoney ER, Stockard J, et al. Development and testing of a short form of the patient activation measure. *Health Services Research.* 2005; 40(6 Pt 1): 1918-1930. https://doi.org/10.1111/j.1475-6773.2005.00438.x.

86. Greene J and Hibbard JH. Why does patient activation matter? An examination of the relationships between patient activation and health-related outcomes. *Journal of General Internal Medicine.* 2012; 27(5): 520–526. https://doi.org/10.1007/s11606-011-1931-2.

87. Insigina Health, *Patient activation measure (PAM) 13 TM Licence Materials copyright*. 2011, LLC: Insigna Health.

88. Remmers C, Hibbard J, Mosen DM, et al. Is patient activation associated with future health outcomes and healthcare utilization among patients with diabetes? *The*

For peer review only

For peer review only - http://bmjopen.bmj.com/site/about/guidelines.xhtml

Journal of Ambulatory Care Management. 2009; 32(4): 320-327. https://doi.org/10.1097/JAC.0b013e3181ba6e77.

89. Australian Bureau of Statistics (ABS). 2033.0.55.001 - Socio-Economic Indexes for Areas (SEIFA). 2016 [cited 2021 14 September]; Available from: www.abs.gov.au.

90. World Health Organization (WHO). Body mass index - BMI. c2018 [cited 2021 September 1]; Available from: http://www.euro.who.int/en/health-topics/disease-prevention/nutrition/a-healthy-lifestyle/body-mass-index-bmi.

91. Harris PA, Taylor R, Minor BL, et al. REDCap Consortium. The REDCap consortium: Building an international community of software platform partners. *Journal of Biomedical Informatics.* 2019; 95(103208). https://doi.org/10.1016/j.jbi.2019.103208.

92. Harris PA, Taylor R, Thielke R, et al. Research electronic data capture (REDCap) – A metadata-driven methodology and workflow process for providing translational research informatics support. *Journal of Biomedical Informatics.* 2009; 42(2): 377-381. https://doi.org/10.1016/j.jbi.2008.08.010.

For peer review only

For peer review only - http://bmjopen.bmj.com/site/about/guidelines.xhtml

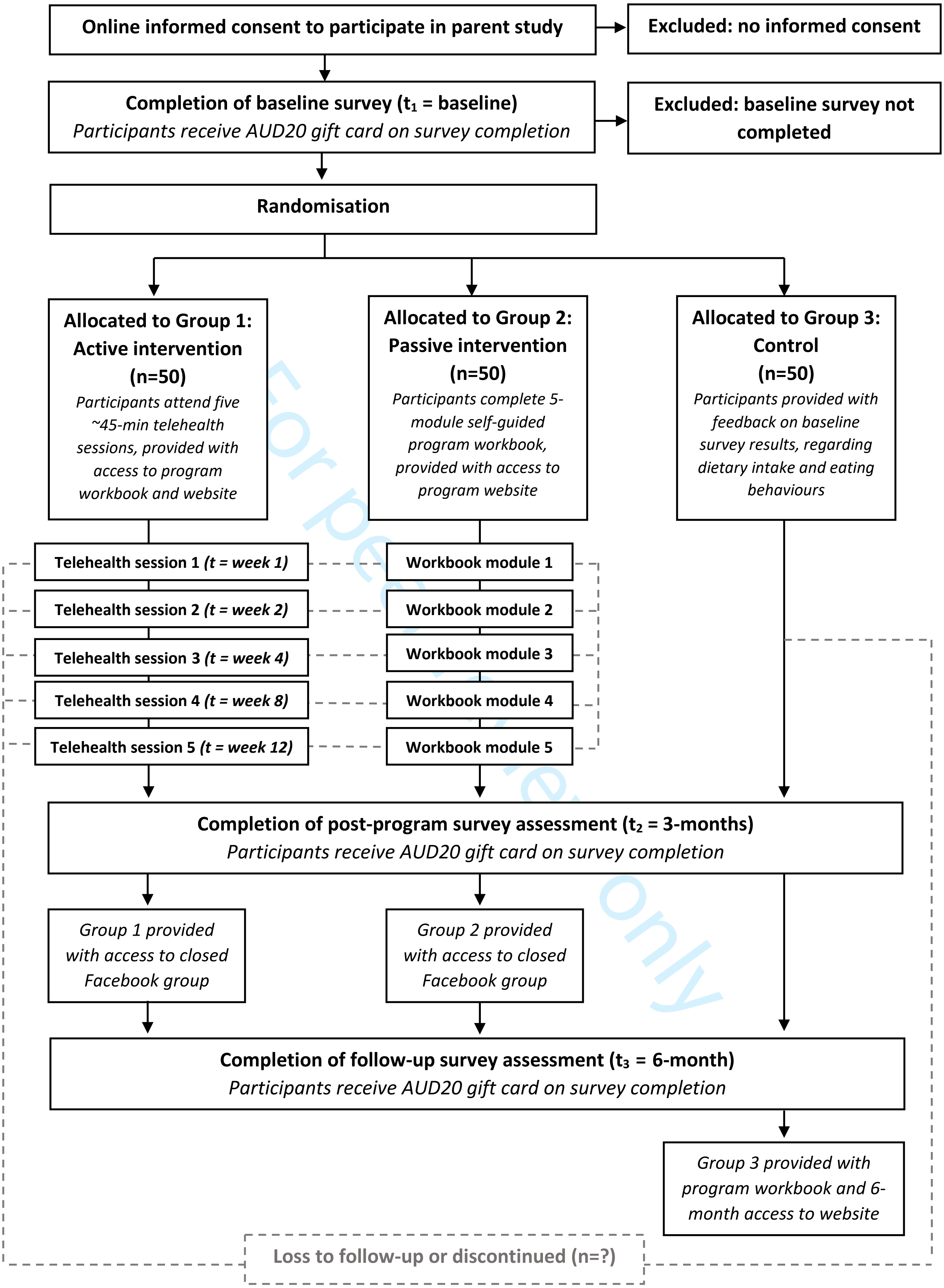
Online informed consent to participate in parent study
Excluded: no informed consent
Completion of baseline survey (t1 = baseline)
Participants receive AUD20 gift card on survey completion
Excluded: baseline survey not completed
Randomisation
Allocated to Group 1: Active intervention (n=50)
Participants attend five ~45-min telehealth sessions, provided with access to program workbook and website
Allocated to Group 2: Passive intervention (n=50)
Participants complete 5-module self-guided program workbook, provided with access to program website
Allocated to Group 3: Control (n=50)
Participants provided with feedback on baseline survey results, regarding dietary intake and eating behaviours
Telehealth session 1 (t = week 1)
Telehealth session 2 (t = week 2)
Telehealth session 3 (t = week 4)
Telehealth session 4 (t = week 8)
Telehealth session 5 (t = week 12)
Workbook module 1
Workbook module 2
Workbook module 3
Workbook module 4
Workbook module 5
Completion of post-program survey assessment (t2 = 3-months)
Participants receive AUD20 gift card on survey completion
Group 1 provided with access to closed Facebook group
Group 2 provided with access to closed Facebook group
Completion of follow-up survey assessment (t3 = 6-month)
Participants receive AUD20 gift card on survey completion
Group 3 provided with program workbook and 6-month access to website
Loss to follow-up or discontinued (n=?)

WELCOME BACK!As you have been found eligible to participate in the personality based intervention for addictive eating, it is important that you carefullly read the following information and give consent at the bottom if you wish to continue to the baseline surveys.

I have read the previous Participant Information Statement and give my consent to participate in this study?

◯ Yes
◯ No

I understand that it is possible that some questionnaires may identify potential health issues that may require follow-up with my GP. I give consent for a copy of the relevant results to be sent to my local doctor/GP or other Health Professional

◯ Yes
◯ No

Please provide Health Professional details (e.g Name, Phone number)

____

I agree to participate in the Personality based intervention for Addictive Eating Behaviours study and give my consent freely.

I understand that the project will be conducted as described in the previous Participant Information Statement, a copy of which I have had the opportunity to download.

I understand I can withdraw from the project at any time and do not have to give any reason for withdrawing.
I am aware I have an equal chance of being allocated into one of three intervention groups. If allocated to:

• Group 1, I consent to completing online questionnaires at the beginning of the study and after 3 and 6 months, and to participating in five telehealth/phone consultations of 30-45mins with an Accredited Practising Dietitian.

• Group 2, I consent to completing online questionnaires at the beginning of the study and after 3 and 6 months, and to complete the self-guided workbook and access the study website.

• Group 3, I consent to completing online questionnaires at the beginning of the study and after 3 and 6 months, and to follow my usual dietary intake for the study duration. I understand that after 6 months I will have access to complete the self-guided workbook and access to the study website.

If allocated to group 1, I consent for my five sessions with the dietitian to be recorded for quality and training purposes.

◯ Yes
◯ No

Please provide your First Name:

____

Please provide your Last Name:

____

Please sign:

____

Do you wish to continue to the Baseline Surveys?

◯ Yes
◯ No

For peer review only - http://bmjopen.bmj.com/site/about/guidelines.xhtml

Title: Examining the efficacy of a telehealth intervention targeting addictive eating in Australian adults (the TRACE program): a randomised controlled trial protocol

Reference to where data collection forms can be found

Survey	Assessment tool	Reference	Available from
**AAS**	**Active Australia Survey**	Australian Institute of Health and Welfare (AIHW) 2003. The Active Australia Survey: a guide and manual for implementation, analysis and reporting. Canberra: AIHW.	https://www.aihw.gov.au/reports/physical-activity/active-australia-survey/summary
**AES**	**Australian Eating Survey**	Ashton L, Williams R, Wood L, Schumacher T, Burrows T, Rollo M, et al. Comparison of Australian Recommended Food Score (ARFS) and Plasma Carotenoid Concentrations: A Validation Study in Adults. Nutrients. 2017;9(8):888. http://doi.org/10.3390/nu9080888<br><br>Collins CE, Boggess MM, Watson JF, Guest M, Duncanson K, Pezdirc K, et al. Reproducibility and comparative validity of a food frequency questionnaire for Australian adults. Clinical Nutrition. 2014;33(5):906-14. doi: 10.1016/j.clnu.2013.09.015	https://australianeatingsurvey.com.au/
**ASSIST**	**Alcohol, Smoking and Substance Involvement Screening Test - Version 3.0**	WHO ASSIST Working Group (2002) The Alcohol, Smoking and Substance Involvement Screening Test (ASSIST): development, reliability and feasibility. Addiction, 97:1183-1194. doi: 10.1046/j.1360-0443.2002.00185.x	https://www.who.int/publications/i/item/978924159938-2
**BES**	**Binge Eating Scale**	Gormally J, Black S, Daston S, Rardin D. The assessment of binge eating severity among obese persons. Addictive Behaviors. 1982;7(1):47-55. https://doi.org/10.1016/0306-4603(82)90024-7	Available in the publication
**CSRI**	**Consumer Services Receipt Inventory**	Beecham J and Knapp M. (2001) Costing psychiatric interventions, in G. Thornicroft (ed.) Measuring Mental Health Needs, Gaskell, 2nd edition, 200-224.	https://www.pssru.ac.uk/csri/what-is-the-csri/
**EDE-Q 6.0**	**Eating Disorder**	Fairburn C, Cooper Z, O'Connor M. Eating disorders examination (16.0D) In: Fairburn C (Ed.), editor. In:	https://nedc.com.au/assets/Medicare-related-forms/Eating-Disorder-Examination-Questionaire-Smart-PDF.pdf

	**Examination Questionnaire 6.0**	Cognitive behavior therapy and eating disorders. New York: Guilford Press; 2008.	
**EDE-QS**	**Eating Disorder Examination Questionnaire Short Form**	Prnjak K, Mitchison D, Griffiths S, Mond J, Gideon N, Serpell L, Hay P. Further development of the 12-item EDE-QS: identifying a cut-off for screening purposes. BMC Psychiatry. 2020;20:146. https://doi.org/10.1186/s12888-020-02565-5	Available as Supporting Information accompanying the publication
**EQ5D-5L**	**EQ5D-5L**	Brazier J, Ratcliffe J, Tsuchiya A, Salomon J. Measuring and Valuing Health Benefits for Economic Evaluation. 2nd ed. Oxford: Oxford University Press; 2016. doi: 10.1093/med/9780198725923.001.0001.	https://aci.health.nsw.gov.au/__data/assets/pdf_file/0003/632847/EuroQol-5-Dimension.pdf
**GAD-7**	**Generalized Anxiety Disorder 7**	Spitzer RL, Kroenke K, Williams JBW, Löwe B. A Brief Measure for Assessing Generalized Anxiety Disorder (The GAD-7). Archives of Internal Medicine. 2006;166(10):1092–7 https://doi.org/10.1001/archinte.166.10.1092	https://adaa.org/sites/default/files/GAD-7_Anxiety-updated_0.pdf
**PAM-13**	**Patient Activation Measure 13-item**	Hibbard JH, Stockard J, Mahoney ER, Tusler M. (2004). Development of the Patient Activation Measure (PAM): conceptualizing and measuring activation in patients and consumers. Health services research. 2004;39(4 Pt 1): 1005–1026. https://doi.org/10.1111/j.1475-6773.2004.00269.x	https://www.insigniahealth.com/products/pam
**PQH-8**	**Patient Health Questionnaire**	Kroenke K, et al., The PHQ-8 as a measure of current depression in the general population. Journal of affective disorders, 2009. 114(1-3): p. 163-173 https://doi.org/10.1016/j.jad.2008.06.026	https://www.psychologywizard.net/uploads/2/6/6/4/26640833/kroenke_phq8.pdf
**PSQI**	**Pittsburgh Sleep Quality Index**	Buysse DJ, Reynolds CF 3rd, Monk TH, Berman SR, Kupfer DJ. The Pittsburgh Sleep Quality Index: a new instrument for psychiatric practice and research. Psychiatry Research. 1989;28(2): 193–213. https://doi.org/10.1016/0165-1781(89)90047-4	https://www.med.upenn.edu/cbti/assets/user-content/documents/Pittsburgh%20Sleep%20Quality%20Index%20(PSQI).pdf
**PSS-4**	**Perceived Stress Scale**	Ingram PB 4th, Clarke E, Lichtenberg JW. Confirmatory Factor Analysis of the Perceived Stress Scale-4 in a Community Sample. Stress Health. 2016; 32(2): 173–176. https://doi.org/10.1002/smi.2592	https://scholar.harvard.edu/files/bettina.hoeppner/files/pss-4.pdf

For peer review only

**REDX-5**	**Reward-Based Eating Drive Scale**	Vainik U, Han C, Epel ES, Dagher A, Mason AE. Rapid assessment of reward-related eating: The RED-X5. Obesity. 2019;27(2):325–31. doi:10.1002/oby.22374	https://www.ncbi.nlm.nih.gov/pmc/articles/PMC6352904/
**SURPS**	**Substance Use Risk Profile Scale**	Woicik PA, Stewart SH, Pihl RO, Conrod PJ. The Substance Use Risk Profile Scale: a scale measuring traits linked to reinforcement-specific substance use profiles. Addictive Behaviors. 2009;34(12): 1042-55. doi: 10.1016/j.addbeh.2009.07.001	Available on request from the corresponding author: Woicik can be contacted at Neuropsychoimaging Group, Brookhaven National Laboratory, Medical Department, Building 490, Upton, New York, 11973, United States. Tel.: +1 631 344 4472. Conrod, NIHR Biomedical Research Centre, Section of Addiction, Department of Psychological Medicine and Psychiatry, King's College London, 4 Windsor Walk, Denmark Hill, London, SE5 8BB, United Kingdom. Tel.: +44 207 848 0836; fax: +44 207 701 8584.p.conrod@iop.kcl.ac.uk
**YFAS 2.0**	**Yale Food Addiction Scale 2.0**	Gearhardt AN, Corbin WR, Brownell KD. Development of the Yale Food Addiction Scale Version 2.0. Psychology of Addictive Behaviors. 2016;30(1):113-21. doi: 10.1037/adb0000136	https://sites.lsa.umich.edu/fastlab/yale-food-addiction-scale/

For peer review only

SPIRIT

STANDARD PROTOCOL ITEMS: RECOMMENDATIONS FOR INTERVENTIONAL TRIALS

SPIRIT 2013 Checklist: Recommended items to address in a clinical trial protocol and related documents*

Section/item	Item No	Description	Addressed on page number
**Administrative information**			
Title	1	Descriptive title identifying the study design, population, interventions, and, if applicable, trial acronym	2
Trial registration	2a	Trial identifier and registry name. If not yet registered, name of intended registry	3, 8
	2b	All items from the World Health Organization Trial Registration Data Set	NA
Protocol version	3	Date and version identifier	-
Funding	4	Sources and types of financial, material, and other support	32
Roles and responsibilities	5a	Names, affiliations, and roles of protocol contributors	1, 32
	5b	Name and contact information for the trial sponsor	1
	5c	Role of study sponsor and funders, if any, in study design; collection, management, analysis, and interpretation of data; writing of the report; and the decision to submit the report for publication, including whether they will have ultimate authority over any of these activities	32
	5d	Composition, roles, and responsibilities of the coordinating centre, steering committee, endpoint adjudication committee, data management team, and other individuals or groups overseeing the trial, if applicable (see Item 21a for data monitoring committee)	NA

For peer review only

For peer review only - http://bmjopen.bmj.com/site/about/guidelines.xhtml

Introduction

Background and rationale	6a	Description of research question and justification for undertaking the trial, including summary of relevant studies (published and unpublished) examining benefits and harms for each intervention	4 – 7
	6b	Explanation for choice of comparators	14
Objectives	7	Specific objectives or hypotheses	7
Trial design	8	Description of trial design including type of trial (eg, parallel group, crossover, factorial, single group), allocation ratio, and framework (eg, superiority, equivalence, noninferiority, exploratory)	8

Methods: Participants, interventions, and outcomes

Study setting	9	Description of study settings (eg, community clinic, academic hospital) and list of countries where data will be collected. Reference to where list of study sites can be obtained	8
Eligibility criteria	10	Inclusion and exclusion criteria for participants. If applicable, eligibility criteria for study centres and individuals who will perform the interventions (eg, surgeons, psychotherapists)	9, 10
Interventions	11a	Interventions for each group with sufficient detail to allow replication, including how and when they will be administered	14 - 20
	11b	Criteria for discontinuing or modifying allocated interventions for a given trial participant (eg, drug dose change in response to harms, participant request, or improving/worsening disease)	8
	11c	Strategies to improve adherence to intervention protocols, and any procedures for monitoring adherence (eg, drug tablet return, laboratory tests)	18, 19
	11d	Relevant concomitant care and interventions that are permitted or prohibited during the trial	NA
Outcomes	12	Primary, secondary, and other outcomes, including the specific measurement variable (eg, systolic blood pressure), analysis metric (eg, change from baseline, final value, time to event), method of aggregation (eg, median, proportion), and time point for each outcome. Explanation of the clinical relevance of chosen efficacy and harm outcomes is strongly recommended	21 - 27
Participant timeline	13	Time schedule of enrolment, interventions (including any run-ins and washouts), assessments, and visits for participants. A schematic diagram is highly recommended (see Figure)	10 - 11 (Table 1)

For peer review only - http://bmjopen.bmj.com/site/about/guidelines.xhtml

Sample size	14	Estimated number of participants needed to achieve study objectives and how it was determined, including clinical and statistical assumptions supporting any sample size calculations	28
Recruitment	15	Strategies for achieving adequate participant enrolment to reach target sample size	8 - 9
**Methods: Assignment of interventions (for controlled trials)**			
Allocation:			
Sequence generation	16a	Method of generating the allocation sequence (eg, computer-generated random numbers), and list of any factors for stratification. To reduce predictability of a random sequence, details of any planned restriction (eg, blocking) should be provided in a separate document that is unavailable to those who enrol participants or assign interventions	13, 14
Allocation concealment mechanism	16b	Mechanism of implementing the allocation sequence (eg, central telephone; sequentially numbered, opaque, sealed envelopes), describing any steps to conceal the sequence until interventions are assigned	13, 14
Implementation	16c	Who will generate the allocation sequence, who will enrol participants, and who will assign participants to interventions	13, 14
Blinding (masking)	17a	Who will be blinded after assignment to interventions (eg, trial participants, care providers, outcome assessors, data analysts), and how	13, 14
	17b	If blinded, circumstances under which unblinding is permissible, and procedure for revealing a participant's allocated intervention during the trial	NA
**Methods: Data collection, management, and analysis**			
Data collection methods	18a	Plans for assessment and collection of outcome, baseline, and other trial data, including any related processes to promote data quality (eg, duplicate measurements, training of assessors) and a description of study instruments (eg, questionnaires, laboratory tests) along with their reliability and validity, if known. Reference to where data collection forms can be found, if not in the protocol	21 - 30, suppl
	18b	Plans to promote participant retention and complete follow-up, including list of any outcome data to be collected for participants who discontinue or deviate from intervention protocols	11 - 13

Data management	19	Plans for data entry, coding, security, and storage, including any related processes to promote data quality (eg, double data entry; range checks for data values). Reference to where details of data management procedures can be found, if not in the protocol	29, 30
Statistical methods	20a	Statistical methods for analysing primary and secondary outcomes. Reference to where other details of the statistical analysis plan can be found, if not in the protocol	28, 29
	20b	Methods for any additional analyses (eg, subgroup and adjusted analyses)	28, 29
	20c	Definition of analysis population relating to protocol non-adherence (eg, as randomised analysis), and any statistical methods to handle missing data (eg, multiple imputation)	29
**Methods: Monitoring**			
Data monitoring	21a	Composition of data monitoring committee (DMC); summary of its role and reporting structure; statement of whether it is independent from the sponsor and competing interests; and reference to where further details about its charter can be found, if not in the protocol. Alternatively, an explanation of why a DMC is not needed	29, 30
	21b	Description of any interim analyses and stopping guidelines, including who will have access to these interim results and make the final decision to terminate the trial	NA
Harms	22	Plans for collecting, assessing, reporting, and managing solicited and spontaneously reported adverse events and other unintended effects of trial interventions or trial conduct	30
Auditing	23	Frequency and procedures for auditing trial conduct, if any, and whether the process will be independent from investigators and the sponsor	30
**Ethics and dissemination**			
Research ethics approval	24	Plans for seeking research ethics committee/institutional review board (REC/IRB) approval	8, 31
Protocol amendments	25	Plans for communicating important protocol modifications (eg, changes to eligibility criteria, outcomes, analyses) to relevant parties (eg, investigators, REC/IRBs, trial participants, trial registries, journals, regulators)	NA

Consent or assent	26a	Who will obtain informed consent or assent from potential trial participants or authorised surrogates, and how (see Item 32)	11, 12
	26b	Additional consent provisions for collection and use of participant data and biological specimens in ancillary studies, if applicable	NA
Confidentiality	27	How personal information about potential and enrolled participants will be collected, shared, and maintained in order to protect confidentiality before, during, and after the trial	29, 30
Declaration of interests	28	Financial and other competing interests for principal investigators for the overall trial and each study site	32
Access to data	29	Statement of who will have access to the final trial dataset, and disclosure of contractual agreements that limit such access for investigators	29, 30
Ancillary and post-trial care	30	Provisions, if any, for ancillary and post-trial care, and for compensation to those who suffer harm from trial participation	NA
Dissemination policy	31a	Plans for investigators and sponsor to communicate trial results to participants, healthcare professionals, the public, and other relevant groups (eg, via publication, reporting in results databases, or other data sharing arrangements), including any publication restrictions	31
	31b	Authorship eligibility guidelines and any intended use of professional writers	NA
	31c	Plans, if any, for granting public access to the full protocol, participant-level dataset, and statistical code	31
**Appendices**			
Informed consent materials	32	Model consent form and other related documentation given to participants and authorised surrogates	Suppl.
Biological specimens	33	Plans for collection, laboratory evaluation, and storage of biological specimens for genetic or molecular analysis in the current trial and for future use in ancillary studies, if applicable	NA

*It is strongly recommended that this checklist be read in conjunction with the SPIRIT 2013 Explanation & Elaboration for important clarification on the items. Amendments to the protocol should be tracked and dated. The SPIRIT checklist is copyrighted by the SPIRIT Group under the Creative Commons "Attribution-NonCommercial-NoDerivs 3.0 Unported" license.
