# Supplementary material for: Examining the efficacy of a telehealth intervention targeting addictive eating in Australian adults (the TRACE Programme): a randomised controlled trial protocol
Source: BMJ Open. 2023 Jun 6;13(6):e064151. doi: 10.1136/bmjopen-2022-064151 (PMC10255192; doi:10.1136/bmjopen-2022-064151)
Supplement: Reviewer comments [file bmjopen-2022-064151.reviewer_comments.pdf]

PEER REVIEW HISTORY

BMJ Open publishes all reviews undertaken for accepted manuscripts. Reviewers are asked to complete a checklist review form (**http://bmjopen.bmj.com/site/about/resources/checklist.pdf**) and are provided with free text boxes to elaborate on their assessment. These free text comments are reproduced below.

ARTICLE DETAILS

**TITLE (PROVISIONAL)**	Examining the efficacy of a telehealth intervention targeting addictive eating in Australian adults (the TRACE program): a randomised controlled trial protocol
**AUTHORS**	Skinner, Janelle; Whatnall, Megan; Leary, Mark; Collins, Rebecca; Pursey, Kirrilly; Verdejo-García, Antonio; Hay, Phillipa; Baker, Amanda; Hides, Leanne; Paxton, Susan; Wood, Lisa; Colyvas, Kim; Collins, Clare; Burrows, Tracy

VERSION 1 – REVIEW

**REVIEWER**	Greenwood, Hannah<br>Bond University, Institute for Evidence-Based Healthcare
**REVIEW RETURNED**	26-Jul-2022

**GENERAL COMMENTS**	Congratulations on your well written and reported randomised trial protocol. I enjoyed reading your work, and am interested in your idea of a telehealth intervention to reduce addictive eating. Although I have made a number of suggestions, most of these are aimed at further strengthening your already strong protocol that is deserving of publication. I have tried to provided concrete suggestions for how to make these changes. Well done, and all the best with your trial.

**REVIEWER**	Soundy, Andrew<br>University of Birmingham
**REVIEW RETURNED**	19-Jan-2023

**GENERAL COMMENTS**	Abstract<br>Could you have the aims at the end of the introduction and then methods being just methods. May be add in key eligibility criteria. Is it worth identifying primary and secondary outcome nearer each other?<br><br>Introduction<br>Do you want a definition of addictive eating upfront? Considering the readership of the journal?<br>Don't quite get the sentence endorse symptoms for active eating – what symptoms?<br>How much higher prevalence in a 'higher weight status' and what is this and who is it compared to aged and gender matched control? When you say people with addictive eating have lower diet quality and high intakes – so what? – again as I progress I think I could make similar comments but please consider these sort of comments below

	I think a clearer steer towards why MI and personality risk targeted coping skills needs to be considered – will the reader know what these are also?<br>I am less interested in your pilot work and more interested in the novelty of this work, I would like to know explicitly what gap is being filled, what is not known, why do what you have proposed based on other studies? Is this a world first? if so state it, if not summarise across past research.<br><br>Results<br>Discrimination of content and consider repetition early on my preference would be for the design and checklist information only<br>I'm not clear on eligibility – this is mentioned in the setting section I think that is confusing – setting is where this will occur?<br>In the setting section - When you say adults living in Australia – do you mean you can access potentially every adult?<br>In recruitment section tell me about your sampling technique – is it probabilistic?<br>In your eligibility section you say a BMI of >18.5 – but in the introduction you say the diet of this population group is bad – so its bad but no excess weight problem in the group?<br>Explain why endorse of >/= 3 on the Yale scale briefly<br>Can you provide a reference to support stratification by the selected groups<br>You mention Tidier – the Tidier article Table 1 gives sub-titles I like to use when describing different arms of a trial – could this be employed here?<br>PPI = not sure why consumers weren't involved in the development of this study or why you don't plan to have a steering group? May have missed that.<br><br>Discussion<br>Broader discussion if there is other literature out there rather than just reference to your feasibility work

**REVIEWER**	Hammerle, Florian<br>Universitätsmedizin Mainz, Klinik und Poliklinik für Kinder- und Jugendpsychiatrie und -psychotherapie
**REVIEW RETURNED**	06-Feb-2023

**GENERAL COMMENTS**	Thank you very much for the opportunity to review the manuscript: "Examining the efficacy of a telehealth intervention targeting addictive eating in Australian adults (the TRACE program): a randomised controlled trial protocol" describing a study protocol of a randomised controlled trial of a telehealth intervention.<br><br>The topic and the protocol is very interesting.<br><br>The RCT seems very carefully considered and I appreciate the stepped approach with a feasibility study first and the modification of the original program with the feedback of participants and facilitators very much! The manuscript is very comprehensive and the design with a three-arm comparison including several relevant outcome measures is very sound. Even more, I appreciate the adding of a cost-analysis very much.<br><br>I just have on main issue, which relates to suicidality (especially as inclusion/exclusion criteria and as adverse event/serious adverse event):

	Line 477: Please provide the rational to exclude the question relating to suicidal ideation within the PHQ-9. The psychometric properties could be biased when items are excluded. The cut-offs for severity ratings for this instrument are not provided in the referenced publication. Where have you found the cut-offs?<br><br>Exclusion criteria do not name suicidality. Is suicidality subsumed within severe mental illness? If so, please include it in the manuscript. Do you exclude patients who exhibit suicidality while participating in the trial? Please provide further information. Thank you!<br><br>A small informal point: I read that you included analysis based on data from google analytics and facebook. Maybe this is an issue in Germany, but I was wondering how you handle the data safety aspect with the participants?

VERSION 1 – AUTHOR RESPONSE

Response to Reviewer 1 comments

Major comments

1) The planned intervention will be delivered via telehealth. Please provide stronger rationale in the introduction for the decision to utilise telehealth for program delivery, rather than face-to-face. Consider including benefits/ease of access to participants, removal of geographical restriction issues with face-to-face delivery (i.e., participants can be from anywhere in Australia, rather than one geographic area), and appropriate references illustrating that telehealth is a viable alternative for delivering healthcare interventions.

Author response: Thank you for your suggestion. The following has been added to the introduction (lines 193 - 199) to provide a stronger rationale for the use of telehealth: “Telehealth has been shown to be a strategy to increase reach, with virtual sessions being comparable to face-to-face programs, and to increase access to services without compromising effectiveness.[53] Telehealth will allow participants from anywhere in Australia to participate from home, and will reduce the demands on time and cost of travel.[53] Additionally, telehealth may overcome client-centred barriers by allowing a safe atmosphere for some participants to better engage and discuss more sensitive topics that they would not normally raise.[54]”

2) In the introduction (line 98-99), it is mentioned that addictive eating commonly co-occurs with binge eating disorder. Since addictive eating remains a contentious concept (line 91) it will be crucial for readers to be able to understand the key differences and overlap between these seemingly similar issues. This may include further justification for why addictive eating behaviours are targeted, rather than binge eating behaviours.

Author response: Thank you for your suggestion. The following has been added to the introduction (lines 125 - 140) to provide an understanding of the key differences and overlap between food addiction and binge eating disorder: “Approximately 50% of individuals with BED meet criteria for ‘food addiction’ according to the YFAS.[12] The present state of the literature demonstrates there is considerable overlap between BED and addictive eating.[19, 23] Commonalities include the loss of control over consumption, continued overuse despite negative consequences, and repeated failed attempts to reduce consumption.[23] However, differences have been observed. In BED large amounts of food are consumed over discrete periods of time, and caloric intake can vary markedly from day to day depending on the number of binge episodes experienced.[24, 25] Whereas, addictive

eating behaviours in some can occur over the entirety of a day.[26] Anecdotally, individuals with addictive eating often report the quantity of food consumed remains consistent from day to day. Further, individuals with addictive eating report preferences for specific foods, typically those high in added fat, and/or refined carbohydrates,[18] and not on the consumption of all foods in general as may be presented in BED. At this time, it is unclear if addictive eating will emerge as a severe subtype of BED or be regarded as a distinct form of an addiction disorder. This distinction will be important to allow for targeted treatment and prevention strategies in susceptible individuals."

3) Lines 133, 171, 715: At several points, the authors say that the intervention is described using the TIDieR checklist. While the intervention is well-reported in text, given reference to this checklist specifically, readers may anticipate that a completed checklist would be available as an appendix, like SPIRIT checklist.

Author response: A TIDieR checklist of the intervention protocol (active intervention group) has previously been published (Please see https://pubmed.ncbi.nlm.nih.gov/35672064/). Readers are now directed to this on line 189 of the manuscript as follows (See [51] for the TIDieR checklist of the intervention)."

4) I have some concerns with the reliance on targeting of 'personality risk factors' in the intervention, given that personality is changeable overtime, difficult to measure, reliant on self-report, and contentious in the literature. The scale used to measure this (lines 540-544) and tailor the intervention is validated and seems to assess traits and behaviours related to addiction, rather than dubious 'personality'. I'm not requesting any changes here, but I urge the authors to consider the framing of the study and intervention around personality, perhaps instead using terms like 'traits associated with risk of addictive behaviour', as it could attract undue criticism of what is an otherwise robustly designed trial.

Author response: Thank you for raising this concern. We have changed some of the wording in the manuscript and softened some of the language to better reflect that the intervention will provide "coping skills training for traits associated with risk of addictive behaviour" rather than "personality risk targeted coping skills", for example lines 168 - 169. Where possible, the word "personality" has been replaced with "traits associated with personality and risk of addictive behaviour", see lines 169, 301, 324, 388, 392, and Tables 1 and 2.

5) Lines 606-610: I cannot find complete justification of the calculated sample size. The authors state 'to detect a mean 2-unit difference (SD = 2.2) in the YFAS symptoms between [groups], and using a standardised effect size of d=0.91, a sample size of 32 individuals per group [total 96] is required to detect this change with [0.90 power and accounting for multiple testing]. I have no issue with the authors definition for what is clinically-meaningful, the power or type 1 error rate applied, but I recommend the authors clarify the use of a standardised effect size of d = 0.91 (which is a large effect, according to Cohen's effect size estimates). Given that the adjusted effect difference from the feasibility study was 0.7, and no other justification is given for why the authors expect this intervention to produce a large effect, it is unclear why the authors chose to calculate sample size based on d=0.91. While I understand that recruitment is already finalised, this could create issues with the interpretation of the study results (i.e., if no difference is found between the interventions and control, only a large effect of the interventions can be excluded)

Author response: Thank you for the feedback. It is important to clarify differences between statistical significance and clinical significance as often these can be quite different in numbers and meaning. In

this instance, for the intervention to be clinically meaningful, and have relevance for future real-world application, it needs to achieve a clinically meaningful difference. In order to achieve this goal of the research a large effect size was required. Through guidance with statisticians, a large effect size was chosen and needed to enable the possibility of a clinically meaningful result.
To provide justification for our choice of effect size, the following sentence has been added to the manuscript on lines 696 - 697: “Through guidance with statisticians, a large effect size was chosen and needed to enable the possibility of a clinically meaningful result.”

Minor/specific comments

6) Although the protocol is well reported using the SPIRIT checklist, there are a couple of points that could be improved.
- Item number 18a: include a copy of the data collection forms in an appendix or link to where they may be found
- Item number 20c: clarify whether intention to treat analysis will be conducted (lines 636/637 says allocated randomisation group)
- Item number 21a: if a data monitoring committee is not being use, provide an explanation for why not.
- Item number 31c: include plan for public access to protocol/dataset/code, even if the plan is to not make this publicly available.
- Item number 32: attached the consent form used as an appendix

Author response:
Item 18a: Links to where data collection forms can be found has now been included as Supplementary Material and readers are directed to this on line 519 of the manuscript.
Item 20c: To provide clarification, lines 731 - 732 have been amended and now reads “The participants will be analysed according to their allocated randomisation group consistent with an intention-to-treat analysis.”
Item 21a: The following statement has been added to manuscript (lines 758 - 764) “A Data Safety Monitoring Board will not be established for this study as all elements of the intervention have been previously explored and used in interventions. To monitor for potential risks, the study co-ordinator managing the day-to-day conduct of the trial, and facilitators of the telehealth sessions, will report weekly to the Chief Investigator. Oversight concerning the overall conduct of the trial will be provided by our multi-disciplinary research team. This will include regular meetings to review protocol adherence, participant retention rates and safety reports.”
Item 31c: The following statement has been added to manuscript (lines 789 - 791) “Data from the TRACE study will be made available in the future for collaborative research questions. Such requests must be authorised by the principal investigators and the appropriate Human Research Ethics Committees.
Item 32: A pdf copy of the electronic consent form has now been included as Supplementary Material and readers are directed to this on lines 219 – 220 of the manuscript “(see Supplementary Material for a copy of the consent form)”.
The SPIRIT checklist has been updated to reflect the above changes.
7) Line 93: Please provide a definition of ‘addictive eating’ in the introduction. It says that 15-20% of the adult population endorse symptoms of addictive eating, but there is no explanation of what these symptoms are (or how they might be different to binge eating disorder – see comment number 2).

Author response: We have amended the language throughout the manuscript to improve clarity, i.e. ‘food addiction’ has been replaced with the term ‘addictive eating’, with the exception of three

occasions where the information we report refers specifically to a ‘food addiction diagnosis’ defined by the YFAS tool (lines 92,107 and 126).

8) Addictive eating and food addiction are used interchangeably. Please choose one term to use throughout for clarity. e.g. line 107

Author response: Thank you for your feedback. A definition of ‘addictive eating’, symptoms, and the tool used to measure symptoms has now been included in the introduction and reads as follows,
Lines 89 – 91: “Addictive eating, theorised as being on the severe end of a spectrum of overeating,[1] is a phenotype of eating behaviour marked by the chronic excessive and dysregulated consumption of food.[2, 3]”
Lines 93 – 107: “… with ongoing debate regarding terms and definitions.[7] Specifically, whether the construct should be described as a behavioural disorder or as a substance-related addiction, whereby certain foods or components in foods are capable of activating an addictive-like process in susceptible individuals.[8] Addictive eating, not categorised as a distinct disorder in the Diagnostic and Statistical Manual of Mental Disorders (DSM)[9] or the International Classification of Disease[10] systems, is most commonly assessed using the Yale Food Addiction Scale (YFAS).[11] The YFAS adapts the DSM criteria for ‘substance-related and addictive disorders’ to specific foods.[9] Developed in 2009[2] and revised in 2016 (YFAS 2.0)[11] according to the DSM-5 criteria, this psychometric tool assesses the presence of 11 symptoms of addictive eating. Symptoms include craving, loss of control, tolerance and withdrawal associated with eating behaviours, the repeated unsuccessful attempts to reduce the consumption of specific foods and maintenance of these behaviours despite adverse physical/emotional/social/interpersonal consequences.[11] The YFAS 2.0 provides two scoring options: a continuous symptom score, reflecting the number of endorsed addiction-like symptoms; and a dichotomous diagnosis of ‘food addiction’.[11] “
See also response to comment 2. A description of the difference between addictive eating and binge eating disorder has been included in the introduction on lines 125 - 140.

9) Lines 130-131: Provide a reference to support that addictive eating is likely ‘transdiagnostic’. This sentence seems out of place here and may be better integrated elsewhere in the introduction.

Author response: As suggested this sentence has been moved to lines 122 -123 of the introduction, and the following references have been added to support that addictive eating is likely ‘transdiagnostic’:
1) Treasure J, et al. Are trans diagnostic models of eating disorders fit for purpose? A consideration of the evidence for food addiction. European Eating Disorder Review, 2018. 26(2): p. 83-91.
2) Wiss DA and Brewerton TD. Incorporating food addiction into disordered eating: the disordered eating food addiction nutrition guide (DEFANG). Eating and Weight Disorders, 2017. 22(1): p. 49-59.

10) Line 452: A definition for the acronym FFQ is not provided in the manuscript

Author response: Thank you for pointing this out. The abbreviation has now been replaced with the full phrase “Food Frequency Questionnaire” on lines 544 and 555.

11) Lines 136-138: It is a bit unusual to hypothesise about the results reaching statistical significance, since this is just the benchmark for determining whether there is a difference between

groups (along with clinical significance, which the authors also include). Perhaps this could be expressed simply as: 'It is hypothesised that both the active and passive intervention groups will achieve reduced addictive eating symptoms relative to the control group'

Author response: Thank you for the feedback. This sentence has been amended as suggested 'It is hypothesised that both the active and passive intervention groups will achieve a reduction in addictive eating symptoms relative to the control group' (lines 203 - 205).

12) Line 188: If available, please include a link to the tailored recruitment videos

Author response: Presently, public links to our recruitment videos are not available.

13) Line 217: Specify which specific physical health conditions were excluded

Author response: The manuscript has been updated with additional information to be more transparent and align with our Participant Information Statement. Lines 253 - 254 now reads "… have a health condition that necessitates taking medications which affect dietary intake or weight status"

14) Line 311: State why blinding of participants and dietician is not possible (i.e., due to the nature of the active intervention)

Author response: Thank you for the suggestion. This sentence has been amended and now reads 'Due to the telehealth nature of the active intervention, blinding of participants and dietitians to intervention group allocation in this study will not be possible.' (Lines 352 -353)

Other suggestions

15) Limit use of abbreviations, especially if used less than 3 times and not commonly known

Author response: Abbreviations throughout the manuscript have been checked. The abbreviation FFQ having been used less than 3 times has now been replaced with the full phrase i.e. Food frequency Questionnaire.

16) It is unusual to see a discussion in a protocol, and not expected with the SPIRIT checklist. Consider merging the important points in the discussion section into the introduction to reduce overall length of manuscript and avoid unnecessary repetition.

Author response: Thank you for your suggestion. We have merged the important points of the discussion section into the introduction, and the limitations previously described in the discussion section are now included under the heading 'Limitations' on line 794 of the manuscript.

17) Similarly, some details under the 'Study trial design' section of the methods (lines 146 to 165) could be merged into the other relevant sections in the methods.

Author response: As suggested, some details under the 'Study trial design' section of the methods have merged into the other relevant sections of the methods. See lines 362 – 376 (lines 151 -165 from the original manuscript).

Response to Reviewer 2 comments

1) Abstract: Could you have the aims at the end of the introduction and then methods being just methods. May be add in key eligibility criteria. Is it worth identifying primary and secondary outcome nearer each other?

Author response: Thank you for your feedback. The abstract has been amended (lines 43 - 50). As suggested, the primary aim has been moved to the introduction section, and key eligibility criteria added to the method section. Additionally, primary and secondary outcomes have been moved nearer to each other in the methods section.

2) Introduction: Do you want a definition of addictive eating upfront? Considering the readership of the journal?

Author response: In response to your suggestion, and that of Reviewer 1, a definition of addictive eating has now been added to the introduction on lines 89 - 91 and reads as follows: "Addictive eating, theorised as being on the severe end of a spectrum of overeating,[1] is a phenotype of eating behaviour marked by the chronic excessive and dysregulated consumption of food.[2, 3]"

3) Introduction: Don't quite get the sentence endorse symptoms for active eating – what symptoms?

Author response: To improve understanding of the endorsement of addictive symptoms, a description of addictive eating symptoms, and the YFAS tool that assesses these symptoms (previously included in the methods section), has now been incorporated into the introduction. See lines 96 – 107: "Addictive eating, not categorised as a distinct disorder in the Diagnostic and Statistical Manual of Mental Disorders (DSM)[9] or the International Classification of Disease[10] systems, is most commonly assessed using the Yale Food Addiction Scale (YFAS).[11] The YFAS adapts the DSM criteria for 'substance-related and addictive disorders' to specific foods.[9] Developed in 2009[2] and revised in 2016 (YFAS 2.0)[11] according to the DSM-5 criteria, this psychometric tool assesses the presence of 11 symptoms of addictive eating. Symptoms include craving, loss of control, tolerance and withdrawal associated with eating behaviours, the repeated unsuccessful attempts to reduce the consumption of specific foods and maintenance of these behaviours despite adverse physical/emotional/social/interpersonal consequences.[11] The YFAS 2.0 provides two scoring options: a continuous symptom score, reflecting the number of endorsed addiction-like symptoms; and a dichotomous diagnosis of 'food addiction'.[11]"

4) Introduction: How much higher prevalence in a 'higher weight status' and what is this and who is it compared to aged and gender matched control? When you say people with addictive eating have lower diet quality and high intakes – so what? – again as I progress I think I could make similar comments but please consider these sort of comments below

Author response: Thank you for your feedback. To improve clarity additional details have been added to the introduction on lines 111 – 120 : “Higher prevalence rates of addictive eating have been reported in individuals with higher Body Mass Indexes (BMI) classified as overweight or obese compared to lower BMIs.[13, 15] Although addictive eating is not exclusive to those with higher weight status.[16] It has been suggested that addictive eating in those with underweight may be related to dietary restriction practices (e.g., consuming more than intended that breaches self-imposed dietary rules, intense craving resulting from extreme dieting practices).[16, 17] Irrespective of weight status, results from recent research indicate that individuals with addictive eating have significantly lower diet quality and higher intakes of highly processed foods.[14, 18, 19] Poor diet is a significant contributor to early death globally[20] and addressing addictive eating may contribute to the prevention or management of adverse health outcomes.”

5) Introduction: I think a clearer steer towards why MI and personality risk targeted coping skills needs to be considered – will the reader know what these are also?

Author response: In response to this suggestion, the following has been added to the introduction on lines 165 - 172: “The traits that have been linked to addictive eating include impulsivity, sensation seeking, and anxiety and depression proneness.[44-48] Findings suggest that individuals with addictive eating may be highly aware of emotions, but lack the skills needed to cope with negative affect.[49] Using personalised coping skills for traits associated with personality and the risk of addictive behaviour in combination with MI, a communication approach used to identify and resolve ambivalence between desired behaviors and actual behaviors to increase motivation,[50] may be affective to facilitate behaviour change in individuals with addictive eating.”

6) Introduction: I am less interested in your pilot work and more interested in the novelty of this work, I would like to know explicitly what gap is being filled, what is not known, why do what you have proposed based on other studies? Is this a world first? if so state it, if not summarise across past research.

Author response: To provide more justification for our proposed work the following text has been added to the introduction:
Lines 150 - 156, “Since publication of this review, a further four intervention studies (a behavioural weight loss program, [35] a brief telephone-based cognitive behavioural therapy intervention,[36] a low carbohydrate dietary program,[37] and a probiotic supplement placebo-controlled trial[38]) have been trialled. All four studies reported an improvement in addictive eating symptomatology immediately following the intervention, but two studies[35, 36] noted this improvement was not sustained over the longer-term.”
Lines 158 - 161, “Given the limited number of treatment options for addictive eating, there is a clear need for services, and development and testing of interventions. It has been suggested that interventions based on substance use addiction models may be effective at facilitating changes in eating behaviour.[38]”
Lines 191 - 192, “To the authors knowledge, the TRACE program is the first MI-based telehealth intervention involving personalised coping skills training for the management of addictive eating in adults.”

7) Methods: Discrimination of content and consider repetition early on my preference would be for the design and checklist information only

Author response: Thank you for your feedback. Several areas of the manuscript have now been merged and content amended/removed to minimise repetition. For example, the discussion has been merged into the introduction.

8) Methods: I'm not clear on eligibility – this is mentioned in the setting section I think that is confusing – setting is where this will occur?

Author response: The 'Setting' section has been amended and now reads: "The active intervention will be delivered via telehealth sessions, conducted in Australia, and supported by a program workbook, and website containing materials relevant to the intervention." (Lines 224 - 226) Eligibility inclusion/exclusion criteria is listed under the 'Eligibility' section on lines 241 – 256.

9) Methods: In the setting section - When you say adults living in Australia – do you mean you can access potentially every adult?

Author response: The active intervention will be delivered via telehealth sessions to adults who respond to the recruitment material for the study, therefore any adult living in Australia meeting the eligibility criteria can participate in the study.

10) Methods: In recruitment section tell me about your sampling technique – is it probabilistic?

Author response: The following has been added to the manuscript (lines 232 -234) to describe the sampling technique used "A non-probability sampling technique (voluntary response sampling)[54] will be used, and recruitment will continue until the desired number of participants is achieved."

11) Methods: In your eligibility section you say a BMI of >18.5 – but in the introduction you say the diet of this population group is bad – so its bad but no excess weight problem in the group?

Author response: Based on past research, it is expected that participants will fall across a range of weight statuses i.e., BMIs in the healthy, overweight and obese ranges. However, the active/passive intervention is not a weight loss program. The intervention was designed to promote lifestyle and behaviour change including improved eating patterns, increased engagement in physical activity, and improved sleep.

12) Methods: Explain why endorse of >/= 3 on the Yale scale briefly

Author response: Three or more symptoms is the YFAS tool scoring instructions definition for mild to severe addictive eating behaviours. The following lines have been amended to reflect this reasoning:
Lines 243 -244, "Endorse ≥ 3 symptoms on the Yale Food Addiction Scale 2.0 (i.e. exhibiting mild to severe addictive eating)[8]"
Lines 531 - 532, "… severity of addictive eating will be classified in accordance with YFAS scoring instructions as follows: "mild" = 3 symptoms, "moderate" = 4-5 symptoms or "severe" ≥6 symptoms."

13) Methods: Can you provide a reference to support stratification by the selected groups

Author response: References have been added to lines 347 – 348 as follows "…in past cross-sectional research (for example, [12, 13, 15, 69])."
Further, the text relating to the randomisation procedure has been reordered and additional details provided so there is sufficient information to be reproducible. Lines 342 – 346 "…participants will be stratified into 4 groups by sex and mental health status (presence or absence, based on either depression, scale PHQ-8 scores ≥15 or below 15, or anxiety scale GAD-7 scores ≥11 or below 11). Participants within each of these four groups will be randomised to one of the three study groups in equal ratios using permuted block randomisation, with block sizes of six."

14) Methods: You mention Tidier – the Tidier article Table 1 gives sub-titles I like to use when describing different arms of a trial – could this be employed here?

Author response: Thank you for your suggestion. While the headings remain the same to align with SPIRIT guidelines, readers are now directed on line 189 to our published TIDieR checklist as follows (See [51] for the TIDieR checklist of the intervention)".

15) Methods: PPI = not sure why consumers weren't involved in the development of this study or why you don't plan to have a steering group? May have missed that.

Author response: While we do not have a formal steering group, a collaborative approach has been undertaken with consumers (i.e., individuals with lived experience of addictive eating and healthcare experts) and is ongoing. Consumers have been involved in a range of ways (summarised on lines 481 – 514 of the manuscript). This includes review of our previous pilot study and participation in the process evaluation procedure, design of program materials and review of program concepts. For example, in response to the participant feedback received from the process evaluation of our pilot study, priority was given to the review and enhancement of the TRACE program and program materials prior to this study's commencement.
We note in the manuscript "Consumers were not involved in the design of the current study, the selection of outcome measurements, research questions or the recruitment of additional participants". The following has been added to lines 509 - 514 to provide further clarification on consumer involvement: "However, consumers were involved in the overall concepts employed in the study and may be called upon at the dissemination stage. For example, to review plain language summaries of the results, provide advice on ways to communicate/translate our findings, or present our findings to the community."

16) Discussion: Broader discussion if there is other literature out there rather than just reference to your feasibility work

Author response: In response to Reviewer 1's comment: "It is unusual to see a discussion in a protocol, and not expected with the SPIRIT checklist. Consider merging the important points in the discussion section into the introduction to reduce overall length of manuscript and avoid unnecessary repetition" we have merged the discussion section into the introduction.

To our knowledge, only 12 intervention studies for addictive eating have been conducted to date. This includes 4 studies published after our initial manuscript submission. This literature is discussed in the introduction on lines 145 – 156.

Response to Reviewer 3 comments

1) I just have on main issue, which relates to suicidality (especially as inclusion/exclusion criteria and as adverse event/serious adverse event): Line 477: Please provide the rational to exclude the question relating to suicidal ideation within the PHQ-9. The psychometric properties could be biased when items are excluded. The cut-offs for severity ratings for this instrument are not provided in the referenced publication. Where have you found the cut-offs?

Author response: Thank you for your feedback. In the current manuscript, the text and references have been updated to reflect that the eight-item Patient Health Questionnaire depression scale (PHQ-8) will be used rather than the PHQ-9, and the following sentence has been removed: "For this study, the question within the PQH-9 relating to suicide ideation was not included." Cut-offs for the PHQ-8 have been referenced (Line 571).
The question relating specifically to suicide ideation was left out of the current study because it would be difficult to regularly monitor participant responses in real time, and there was a risk of someone reporting this and the team not being able to respond appropriately i.e. provide adequate and timely assessment or intervention.
The PHQ-8 has been found to be psychometrically sound. A 2020 meta-analysis of individual patient data (6,742 participants from 54 studies) found high correlations between the PHQ-8 and PHQ-9 scores (0·996) and almost identical scoring thresholds for depression with both tools. [see ref. 1 below] Further, research indicates that the deletion of the suicide ideation question has only a minor effect on scoring and the ninth item is by far the least frequently endorsed item on the PHQ-9." [see ref. 2 below]

References:

1) Wu Y, Levis B, Riehm KE, et al. Equivalency of the diagnostic accuracy of the PHQ-8 and PHQ-9: a systematic review and individual participant data meta-analysis. Psychological medicine. 2020;50(8):1368–1380. https://doi.org/10.1017/S0033291719001314
2) Kroenke K, Strine TW, Spitzer RL, Williams JB, Berry JT, Mokdad AH. The PHQ-8 as a measure of current depression in the general population. Journal of affective disorders. 2009;114(1-3):163-73. https://doi.org/10.1016/j.jad.2008.06.026

2) Exclusion criteria do not name suicidality. Is suicidality subsumed within severe mental illness? If so, please include it in the manuscript. Do you exclude patients who exhibit suicidality while participating in the trial? Please provide further information.

Author response: Participants are advised via the Participant Information Statement that while our survey measures some of the symptoms associated with stress, anxiety and depression, it is not designed to provide a medical diagnosis for any of these conditions. However, if their responses relate to their safety (i.e. they score in the severe range for stress, anxiety or depression), they will be asked if they would like to be informed/not informed about this on the consent form. With their consent, the relevant information will be sent to their nominated General Practitioner.
Unfortunately, the current intervention was not designed for complex health conditions, therefore individuals with severe mental illness will not be eligible to participate in the study. This is noted as a limitation on lines 799 - 803 of the manuscript. If a participant in the trial exhibits any signs of self-harm, the research team will use a method that is commensurate with the severity or urgency of the situation in accordance with the University of Newcastle's Standard Operating Procedures and the adverse event logged with the Human Research Ethics Committee. The study materials also include links to organisations in Australia that provide assistance.

3)	I read that you included analysis based on data from google analytics and facebook. Maybe this is an issue in Germany, but I was wondering how you handle the data safety aspect with the participants?

Author response: Thank you for your query.

Website: Participants will have password protected access to the study website, hosted by Wix.com. All data captured from the website will be encrypted during transit and at rest periods. During runtime the data will be initially stored on the web server back-end in the database in encrypted form. Daily archiving will occur in which the data are backed up to a number of secondary sources, again ensuring the data are secured through encryption and limited authentication and authorisation access protocols. Data used for analysis will be de-identified before entry into the statistical program.

Facebook: Participants will be informed that joining the Facebook group is voluntary. This is part of the study which aims to further support participants with behaviour change. Participants may use their standard Facebook login where other members of the group will be able to see their login name and profile picture. Alternatively, participants can create a pseudo account where they can choose a name that does not identify them if they wish to remain anonymous.

Privacy alert for using the Facebook forum: The forum is a Facebook “secret group” with the following restrictions: 1) Membership is by invitation only; 2) The group will not appear in search results or on participant’s Facebook profile page; and 3) Only group members can see the group information and group posts.

Participants will be advised via the Participant Information Statement of the following appropriate use of language and etiquette for using the social media/discussion forum:

- Members should post in a way that is respectful of other users. Using insulting or offensive language, trolling, or abusing users in any way will not be tolerated and will lead to a warning and removal from the group.
- This is a shared learning environment. Do not harass, insult, taunt, provoke, demean, or personally attack other members. Be friendly to each other.
- Members should be aware of the public nature of the social media platform and it is advised not to over share personal information or post picture of your children in the forum. For personal queries, please contact the researcher directly via contact details outlined in your participant information statement.
- Members shall not share information about the study website and Facebook forum on their personal social media page.
- If members note an issue which contravenes something in the above mentioned rules they are welcome to bring it to the attention of the researcher via contact details as above.
- The Facebook group will be monitored throughout the study period for appropriate use, language and etiquette by routine weekly checking by the administrator of the group who will be a member of the research team. Inappropriate use will be managed through the removal of any such posts as well a written email from the administrator to the offending Facebook member about appropriate use in future. If inappropriate use continues the member will be removed from the group.

VERSION 2 – REVIEW

**REVIEWER**	Soundy, Andrew<br>University of Birmingham
**REVIEW RETURNED**	14-Mar-2023

**GENERAL COMMENTS**	Generally, for me this looks like a well considered trail. Minor points below.<br>Abstract<br>Wasn't sure about the choice of word endorse in first sentence? wonder in the introduction you say MI is effective for behaviour change – then say you want to do a feasibility study? Wonder if you need to be clearer on the gap? so why feasibility and why telehealth? Is the uniqueness combining MI and coping? Methods should start with design?<br><br>Introduction<br>The first paragraph I think could be reworded to get the points in a more efficient way – think it take a while to get to the definition e.g., could you define by the YFAS for the purpose of the study acknowledging that it is not currently identified in either ICD or DSM?<br>I wonder also if the focus on BED distracts the reader from getting to why do the intervention – so in a similar way could this be shortened<br>Are food addicts anonymous and overeaters anonymous representing global organisations or local to a country – context needed<br>For me a little too much on other treatments - whereas I want to know why MI and why combine it with coping? And what is the past literature on this<br>Just to be clear is coping skills training a separate intervention and combined with MI? I am not sure this is clear as coping has its own body of literature? Or is it a sub-form of MI?<br>You identify what this study builds on but I wasn't clear – has any other related work been done? If not can you state this? So the reader is clear what is known, not known and further needs to be considered past your own research groups work? Does the reader need to know a bit about telehealth interventions in this group? More than is given? Information about telehealth seems to be dropped in at the end whereas I think it should finish with the gap and then aim<br>Methods<br>Sample size calculation needed for 50 per group? (I see it below – is it needed here too?) And does the primary outcome need to be mentioned here under trial design? May be arrangement place elsewhere and focus on design?<br>Good to see SPIRIT and CONSORT<br>Can you report interventions details according to Tidier (Hoffman et al., 2014) or something similar – although your details is good<br>Sampling methods non probabilistic – implications and limitations considered? Thinking about statistical analysis and sampling error<br>Bit confused about recruitment being August 2021 and April 2022 ?<br>Can you check your procedures against a tool like ROB2 to ensure bias is accounted for and document this within the methods?<br>Discussion<br>May be worth considering discussion sections of other protocols in BMJ open – thought it could be expanded.

**REVIEWER**	Hammerle, Florian<br>Universitätsmedizin Mainz, Klinik und Poliklinik für Kinder- und Jugendpsychiatrie und -psychotherapie
**REVIEW RETURNED**	17-Mar-2023

GENERAL COMMENTS	Thank you for the opportunity to review the modified manuscript: "Examining the efficacy of a telehealth intervention targeting addictive eating in Australian adults (the TRACE program): a randomised controlled trial protocol“.<br><br>The authors have been responsive and I think the manuscript was improved-thank you. I have no further concerns and am looking forward to a paper describing the results of the study.

VERSION 2 – AUTHOR RESPONSE

Response to Reviewer 2 comments

Abstract

Reviewer comment: Wasn't sure about the choice of word endorse in first sentence? wonder in the introduction you say MI is effective for behaviour change – then say you want to do a feasibility study? Wonder if you need to be clearer on the gap? so why feasibility and why telehealth? Is the uniqueness combining MI and coping? Methods should start with design?

Author response: Thank you for your feedback. The word 'endorse' has been replaced with 'self-report' on line 39 as suggested.

A feasibility study has already been conducted and the current study will build on this work. [see Burrows T, Collins R, Rollo M, et al. The feasibility of a personality targeted intervention for addictive overeating: FoodFix. Appetite. 2021; 156(104974). https://doi.org/10.1016/j.appet.2020.104974].

To make this clearer for the reader, lines 42 – 44 have been amended and now read: "This project builds upon foundations of an addictive eating feasibility study previously conducted, and co-design process involving consumers."

The wording around "Motivational interviewing" has been updated on lines 40 - 42 to more clearly reflect the work and now reads: "Motivational interviewing-based interventions, containing personalised coping skills training, have been found to be effective for behaviour change in addictive disorders (e.g. alcohol use)."

Telehealth was the chosen delivery mode because of the ease of access for participants and to reduce stigma. Due to the imposed word limit, reasons for this mode of delivery have not been described in the abstract but are described in the introduction on lines 158 - 167.

The study design is now mentioned at the start of the methods on lines 46 - 47 as follows "This three-arm randomised controlled trial will recruit….".

Introduction

Reviewer comment: The first paragraph I think could be reworded to get the points in a more efficient way – think it take a while to get to the definition e.g., could you define by the YFAS for the purpose of the study acknowledging that it is not currently identified in either ICD or DSM?

Author response: Thank you for this suggestion, lines 91 - 96 were removed to be more succinct.

Reviewer comment: I wonder also if the focus on BED distracts the reader from getting to why do the intervention – so in a similar way could this be shortened

Author response: In response to a previous reviewer comment, further information was included in the manuscript to describe the key differences and overlap between addictive eating and BED, and to justify why addictive eating behaviours were targeted, rather than binge eating behaviours. However, this section has now been shortened, and lines 130 to 137 have been removed from the manuscript.

Reviewer comment: Are food addicts anonymous and overeaters anonymous representing global organisations or local to a country – context needed

Author response: To provide context, lines 130 - 132 have been amended to read: "Originating in the United States, they now have 10 000+ members worldwide and have been in existence for many years, demonstrating a need for services."

Reviewer comment: For me a little too much on other treatments - whereas I want to know why MI and why combine it with coping? And what is the past literature on this. Just to be clear is coping skills training a separate intervention and combined with MI? I am not sure this is clear as coping has its own body of literature? Or is it a sub-form of MI?

Author response: More information relating to current treatment options was added to the introduction to align with the previous reviewer comments. However, we have reduced the amount of wording pertaining to treatment in the current manuscript on lines 132 – 140 and highlighted that the intervention combines MI with coping skills training on line 148.

Reviewer comment: You identify what this study builds on but I wasn't clear – has any other related work been done? If not can you state this? So the reader is clear what is known, not known and further needs to be considered past your own research groups work? Does the reader need to know a bit about telehealth interventions in this group? More than is given? Information about telehealth seems to be dropped in at the end whereas I think it should finish with the gap and then aim

Author response: The information relating to telehealth has been moved to lines 158 – 167. To provide more information about telehealth in this population group, the following has been added on lines 164 – 167 "Although the effectiveness of telehealth has not been explicitly explored in populations with addictive eating, recent research demonstrates that telehealth can be as effective as in-person care for the management of mental health conditions,[46] including substance use disorders[47] and eating disorders.[48]

As suggested, the introduction now finishes with the gap followed by the aim.

Methods

Reviewer comment: Sample size calculation needed for 50 per group? (I see it below – is it needed here too?) And does the primary outcome need to be mentioned here under trial design? May be arrangement place elsewhere and focus on design?

Author response: Thank you for the suggestion, "(n=50 per group)" has been removed from line 210 of the manuscript.

The sentence relating to the primary outcome has been revised on lines 199 – 201 and now reads "Assessments will be carried out at three timepoints: 1) baseline (pre-intervention), 2) 3-month post-baseline (primary time point), and 3) 6-month post-baseline follow-up assessment."

Reviewer comment: Good to see SPIRIT and CONSORT Can you report interventions details according to Tidier (Hoffman et al., 2014) or something similar – although your details is good

Author response: A TIDieR checklist of the intervention protocol (active intervention group) has previously been published (Please see https://pubmed.ncbi.nlm.nih.gov/35672064/). Readers are directed to this on line 185 - 186 of the manuscript as follows "(See [49] for the TIDieR checklist of the intervention)."

Reviewer comment: Sampling methods non probabilistic – implications and limitations considered? Thinking about statistical analysis and sampling error Can you check your procedures against a tool like ROB2 to ensure bias is accounted for and document this within the methods?

Author response: Thank you for your query. The use of non-probabilistic sampling in the current study was decided in consultation with a statistician. No changes have been made to the manuscript, however biases have been considered and the following steps taken to minimise bias:

- The sample being recruited is appropriate for the hypothesis being tested.
- The recruitment methods being used will encourage Australia wide participation (e.g., advertisements on national radio) and minimise selection errors.
- Stratified randomisation is being used to promote group balance on potential confounding factors, and eliminate selection bias, so that results are suitably generalisable.
- The study will recruit sufficient participants to allow it to have a high probability of detecting a clinically important difference between intervention and control groups if a difference truly exists.
- All three groups will be treated identically in all respects except for the interventions being tested.
- The investigator assessing outcomes will be blinded to treatment allocation.
- Participants will be analysed within the group to which they were allocated, irrespective of whether they experienced the intended intervention (intention-to-treat analysis).
- The analysis focuses on testing the research question according to the a priori hypothesis.

Reviewer comment: Bit confused about recruitment being August 2021 and April 2022?

Author response: At the time of submission to BMJ (25 April 2022) recruitment for the study had just been completed. To maintain transparency this has been stated in the manuscript. However, prior to

recruitment commencing, the trial was submitted for registration with ANZCTR on 6 July 2021 and the protocol information made public.

Discussion

Reviewer comment: May be worth considering discussion sections of other protocols in BMJ open – thought it could be expanded

Author response: Although this contradicts previous reviewer comments, we have added additional text to lines 792 - 794. We leave the inclusion of a more expansive discussion to the editor's discretion.
